# LLMB: AI Agent for Lithium Metal Battery Research Using Large Language Model

**DOI:** 10.1021/acscentsci.5c02433

**Published:** 2026-03-17

**Authors:** Jaewoong Lee, Junhee Woo, Younghun Kim, Sejin Kim, Cinthya Paulina, Hyunmin Park, Hee-Tak Kim, Steve Park, Jihan Kim

**Affiliations:** † Department of Chemical and Biomolecular Engineering, 34968 Korea Advanced Institute of Science and Technology, Daejeon, 34141, Republic of Korea; ‡ Department of Materials Science and Engineering, 34968 Korea Advanced Institute of Science and Technology, Daejeon, 34141, Republic of Korea

## Abstract

Recent advances in data-driven research have shown great potential in understanding the intricate relationships between materials and their performances. Herein, we introduce LLMB, an AI agent for lithium metal battery research that integrates a large language model (LLM) for hierarchical text mining with an automatic graph mining tool, Material Graph Digitizer (MatGD). This agent enables state-of-the-art accurate extraction of battery material data and cyclability performance metrics from diverse sources. Through text mining, we extracted composition and operating condition information for 15,398 battery cells, and graph mining yielded cyclability data for 10,242 cells. By aligning and merging these, we constructed a comprehensive database of 8,074 cells, containing component specifics and capacity. Utilizing the comprehensive database constructed through the LLMB agent, we developed the first machine learning model to predict capacities of LMBs using material information from battery components. Furthermore, molecular simulations and material analyses were performed to elucidate how the identified predictive features influence the physicochemical properties governing the battery performance. Based on these models and material analysis, we experimentally validated that the weakly solvating electrolyte, induced from low EState VSA6 solvents, facilitates the formation of an anion-derived solid-electrolyte interphase (SEI) and promotes highly crystallized Li plating, thereby confirming the reliability of our models.

## Graphical abstract

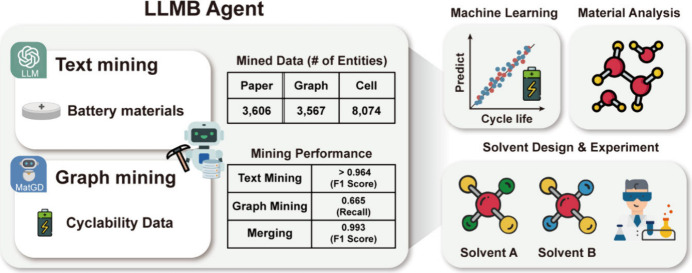

## Introduction

Lithium metal batteries (LMBs) are promising next-generation devices that can achieve high capacity using lithium metal as an anode due to its exceptionally low density (0.534 g cm^–3^), high theoretical capacity (∼3860 mAh g^–1^), and low electric potential (−3.04 V compared to the standard hydrogen electrode). Unfortunately, the high reactivity of lithium metal poses an obstacle to the long-term cyclability of LMBs and research is being conducted in various fields to overcome this issue.^1–4^ Nevertheless, due to delayed performance feedback caused by the extended time required to measure the cycle life of batteries, an early prediction of cycle life from initial performance remains a key challenge that impedes the development-validation cycle of materials and manufacturing processes.^5–8^ The conventional trial-and-error approach is time-consuming and inefficient,^9–14^ especially when dealing with diverse material selection for each component, which can be risky. In addition, these approaches have controlled for only one or two variables^15–17^ (e.g., electrolytes and charge/discharge conditions). Therefore, these fail to provide sufficient insight into discerning comprehensive material–property relationships.

Notably, such information may already exist within the vast body of the published literature. The key challenge lies in how to efficiently extract relevant data and what to analyze. With the advancement of AI, efforts have been made to extract experimental information from battery literature using natural language processing (NLP)^18^ and transformer models.^19,20^ However, previous studies that did not incorporate domain knowledge focused not on entire battery cells but rather on the characteristics of individual battery components. Moreover, these studies were limited by the small number of entities considered and failed to extract quantitative information, such as concentrations or ratios. Furthermore, the absence of automatic graph mining tools made it difficult to obtain performance data from graphs such as specific capacity and cycle stability. These limitations hinder full exploration of the material–property relationship and the utilization of it for machine learning studies. Therefore, unlocking the full potential of innovative battery technologies requires a new approach that leverages comprehensive data to understand the battery cycle. Our goal is to directly provide material insights from decades of accumulated research to guide future studies and innovation.

Here, we present a novel multimodal data-driven approach utilizing a Large Language Model for Battery (LLMB) agent consisting of a large language model^21–23^ (LLM) and an automatic graph mining tool^24^ (Material Graph Digitizer, MatGD). We automatically extracted comprehensive information regarding battery materials with 96.4% accuracy, which is the state-of-the-art performance known to date, encompassing a total of 29 entities, the largest number of entities ever reported. Additionally, for the first time, we successfully mined cyclability performance data directly extracted from cycle graphs. Using this uniquely extensive data set, we developed novel machine learning models capable of predicting battery capacity. The machine learning models not only predict the capacities at the initial and 50th cycles but also provide insights into how the molecular and solvation structures of electrolytes affect cycle performance through the SHapley Additive exPlanations (SHAP) method, which quantitatively identifies how each chemical feature contributes to the predicted value. Furthermore, experimental validation demonstrated that the use of weak solvation solvents (i.e., diethyl ether) improved cycle performance, as evidenced by the formation of an anion-derived thin SEI layer with crystalline Li deposition.

## Results

### Overall Workflow

The overall workflow of our research is schematically illustrated in Figure 1. First, we employed the LLM-based AI agent, LLMB, to automatically perform multimodal mining by integrating text and graph mining on 3,606 papers related to LMBs. Battery research papers were collected using the Scopus API, with a focus on Elsevier journals. This approach was chosen to minimize copyright and cost issues while ensuring diversity in publication formats. This approach enabled the comprehensive extraction of material–property information for individual battery cells. Through text mining, we extracted material-related information, such as cell components, material compositions, and operating conditions. In the graph mining process, we focused on extracting battery property data, particularly cycle performance data. By integrating the material information obtained from text mining with the property data derived from graph mining, we constructed a material–property database encompassing 8,074 battery cells. Further details of this process are described in the subsequent AI Agent: Large Language Model for Battery section.

**Figure 1:**
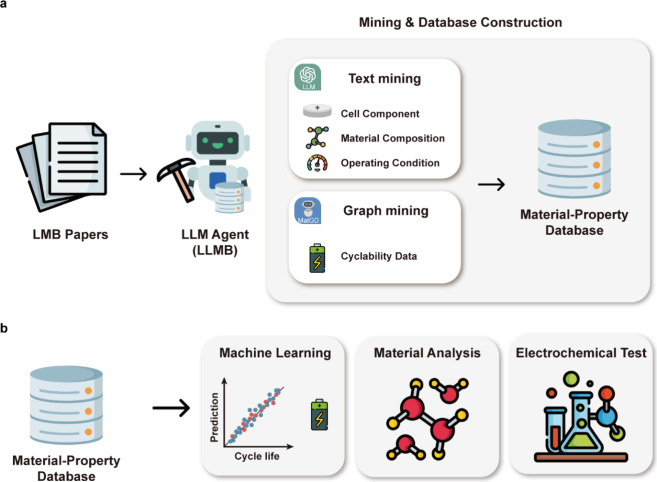
**Machine-learning-assisted data-driven approach in battery science**. A schematic image of the entire process in our research. (a) The LLMB agent automatically extracts text and graph data from the literature. Each stage collects data related to battery materials and properties, including cell components, material compositions, operating conditions, and cyclability. (b) The constructed database was utilized for machine learning, molecular simulation, and material analysis.

To analyze material–property relationships, we developed a machine learning model to predict cycle life based on material information, using the constructed database. We conducted feature importance and SHAP analysis to identify the key material factors influencing the cycle performance. The model’s predictive performance was confirmed through experimental validation. Additionally, molecular simulations were performed to investigate the electrolyte behavior at the atomic scale based on the mined data. Through this integrative approach (Figure 1b), we gained a deeper understanding of the relationship between battery components and cycle performance. Based on insights gained from machine learning analysis and molecular simulations, we designed a new solvent molecule optimized for an enhanced cycle life. Its performance was validated through experimental testing, confirming its effectiveness in improving battery performance.

### AI Agent: Large Language Model for Battery

We developed an automated LLM-based AI agent, LLMB, that extracts comprehensive battery information from both text and graphs in battery research papers and integrates them into a structured database. The LLMB agent operates through three main stages: (1) text mining, (2) graph mining, and (3) database construction. To efficiently carry out these tasks, the LLMB agent is designed with a modular architecture comprising multiple LLMs (GPT-4 model without any fine-tuning), each specialized for a specific task within the overall pipeline (Figure S1).

To identify individual battery cells, the **cell name extraction language model** first identifies the battery cell used in the cycling test, along with the corresponding figure indices, from the captions of the experimental figures (Figure 2a). The extracted battery cell names and figure indices are then used as core identifiers to distinguish the target cells for subsequent mining processes. To further ensure traceability, metadata collected from XML files, such as DOIs, are also incorporated as tags to help identify each cell.

**Figure 2:**
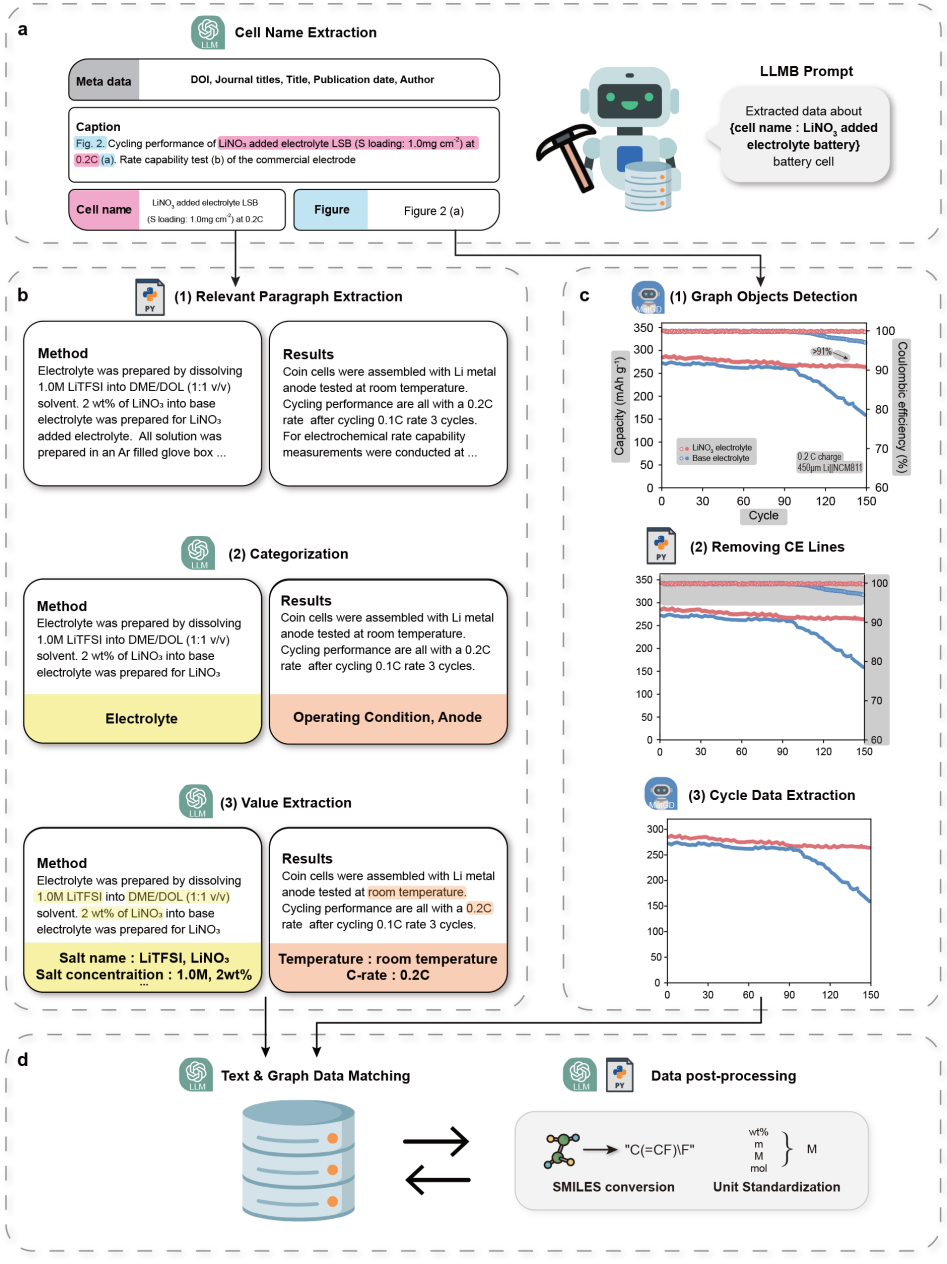
**Schematic illustration of the LLMB agent**. (a) Example of the cell name extraction language model, which extracted cell names and figure indices. (b) Example of the text mining stage. Material composition data were extracted through a stepwise language model consisting of text parsing, categorization, and value extraction. (c) Stepwise process for extracting cyclability data points from graphs using MatGD. (d) Schematic representation of the text and graph data matching language model annotated with metadata, cell name, figure index, and material name. To facilitate effective downstream analyses, postprocessing was performed, including SMILES conversion of the collected material names and standardization of units.

Information related to battery components (e.g., cell components, material compositions, and operating conditions) is widely dispersed throughout a paper, appearing in the captions, results, and methods paragraphs. To efficiently extract relevant information from the text, we adopted a hierarchical architecture that integrates multiple language models that are specialized for specific tasks. To begin the text mining process, a Python script was used to extract relevant paragraphs from the methods and results sections that were associated with the target battery cell (Figure 2b). These extracted paragraphs were then sequentially passed as inputs to a series of language models for categorization and value extraction. In the **categorization model**, each paragraph is classified according to the type of information it contains. This model involves both major categorization and subcategorization (Figure S2, Supplementary Note 1). The categorized paragraphs are passed to the corresponding **value extraction language models**. For each category, a specialized language model is prompt-engineered to extract relevant properties and units specific to that component type, enabling targeted and accurate information retrieval. Table S1 presents the robustness of our prompt structures (Figure S3). The resulting data include a total of 29 entities, such as material names, property values, and units (Figure S4). A complete list of the extracted properties is provided in Table S2.

In the graph mining stage (Figure 2c), we utilized MatGD, an **automatic graph digitizer model** developed in our lab. Cycle graphs were processed using MatGD, which removes text labels, markers, arrows, and other nondata elements, isolating only the data lines. When Coulombic efficiency (CE) curves were present in the graphs, they were removed using a Python-based algorithm that utilized the characteristic behavior of CE, which is typically positioned at the top of the cycle graph and therefore can be readily identified. This filtering allowed us to extract only the cyclability data corresponding to each battery cell. Each extracted data line was saved, together with the corresponding legend label. Additional details regarding the MatGD graph mining tool are provided in the Methods section.

Finally, we integrated the text mining and the graph mining results using a **data matching language model** (Figure 2d) where the inputs comprised the names of battery components and labels of data lines extracted respectively from the two methods. Distinguishing features, such as materials and operating conditions, which are pivotal for identifying different battery cells, are implicitly included within the labels. This approach ensures a high merging accuracy when aligned with names. Note that it also refines hallucinated cells generated during the preceding processes (Table 1). To facilitate effective downstream analyses, including material analysis and machine learning modeling, we applied a **postprocessing model** (Figure 2d). This model was essential to address the diversity of naming conventions and units across thousands of published papers. For components composed of molecules and polymers (e.g., binders, conductive materials, and electrolytes), their names were converted into the Simplified Molecular Input Line Entry System (SMILES) format, which encodes the molecular structure as a text string. Lastly, to unify units involving different dimensions (i.e., concentration and volume ratio of electrolyte), molecular weight and density were used for the conversions (Table S2).

**Table 1:** Summary of the Number of Extracted Papers, Graphs, and Battery Cells, along with the Accuracy, Precision, Recall, and F1 Score at Each Stage of the Mining Process

				**Evaluation metrics**				
	**# of Paper**	**# of Graph**	**# of Cell**	**Accuracy**	**Precision**	**Recall**	**F1 score**	
**Text Mining**	3,606	6,682	15,398	-	0.986	0.942	0.964	Cell
				0.959	0.963	0.989	0.976	Cathode
				0.983	0.985	0.991	0.988	Electrolyte
				0.995	1.000	0.980	0.990	Anode
				0.968	0.958	1.000	0.979	Separator
				0.987	0.986	0.994	0.990	Current collector
				0.979	0.943	1.000	0.971	Operating condition
**Graph Mining**	3,044	5,047	10,242	-	-	0.665	-	
**Final Database**	2,549	3,567	8,074	0.989	0.986	1.000	0.993	Merging

### Data Mining Performance

The statistics and evaluation metrics of the LLMB agent’s performance are summarized in Table 1. To assess the accuracy of each stage (i.e., text mining, graph mining, and database integration), we quantified the number of extracted papers, graphs, and battery cells and evaluated each step using standard metrics: accuracy, precision, recall, and F1 score. In total, 15,398 battery cells were extracted from 3,606 lithium metal battery (LMB) papers obtained from Elsevier journals. To evaluate the performance of the text mining process, a random subset of 384 battery cells from 100 papers was manually analyzed. The extraction of each component showed strong performance with the F1 score reaching 0.990 for current collector identification. Even the lowest-performing category, cell name extraction, achieved a robust F1 score of 0.964. Detailed results of the evaluation metrics are provided in Table S3. Due to the high accuracy of the text mining agent, the number of battery cells extracted through the text mining process was used as the ground truth for evaluating the recall value of the graph mining results. Among the 15,398 identified cells, cyclability data for 10,242 cells were successfully extracted using MatGD, resulting in a recall of 0.655. This recall value reflects a limitation of MatGD’s color-based graph separation method, which may restrict the number of extractable data points (see the Methods section for details). All extracted cyclability data were manually verified to ensure accuracy, contributing to a reliable database. In the evaluation of 265 text–graph matching results from selected papers, we achieved a high F1 score of 0.993. Taken together, these evaluation results demonstrate the efficiency and robustness of our combined text and graph mining strategy. Using the LLMB agent, we successfully constructed a validated database comprising component and cyclability information for 8,074 battery cells. Detailed distributions of extracted information can be found in Figures S5–S9.

### Quantitative Analysis of Extract Data

Through LLMB, we collected various topics in lithium metal battery research papers. First, we used Latent Dirichlet Allocation (LDA), an unsupervised model, to identify the main topics of the LMB papers using their titles and abstracts. As a result, we identified 25 distinct topics, which were grouped by the type of material being modified (Figure 3a). Electrolyte-related topics were the most prevalent, with frequently observed keywords such as “lithium metal”, “high performance”, “stability”, and “ionic conductivity”, highlighting a focus on improving cycling stability of the anode (Figure S10). These results suggest that research on LMBs has been most active in electrolytes for improving cycling stability, followed by cathode materials aimed at increasing capacity.

**Figure 3:**
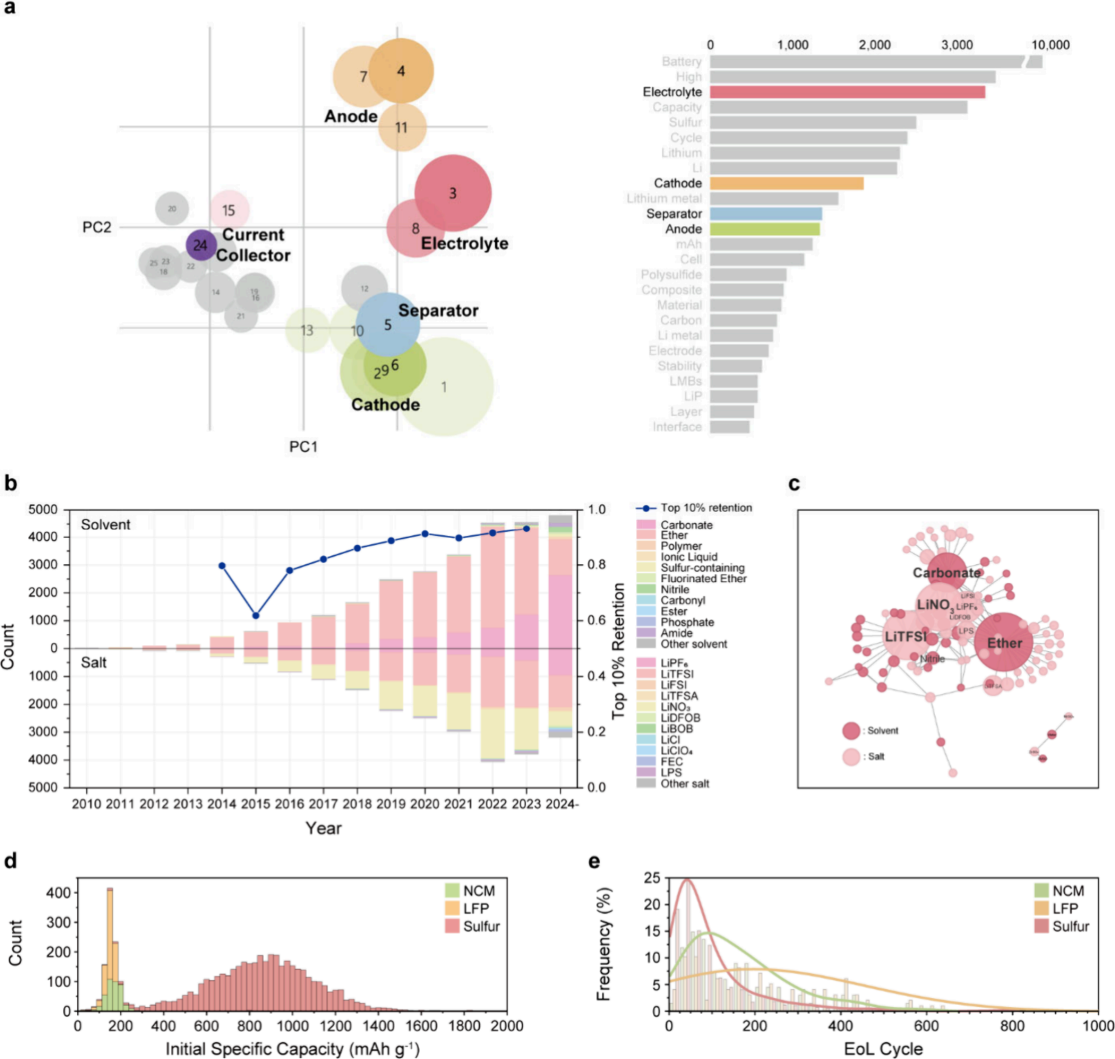
**Trend and explorations of lithium metal batteries**. (a) Intertopic distance map of LMB paper topics, which was obtained by LDA analysis, and distribution of the topics. (b) Stacked histogram showing the distribution of various electrolyte solvent and salt materials published in 3,606 papers. The line graph represents the top 10% capacity retention for different years. (c) Force-directed network graph between the solvents and salts. Molecules that appear more frequently and are used together appear larger and closer to each other. (d) Histogram plot of initial capacity (8,687 cells) and (e) EOL data in the merged database (1,736 cells), with NCM, LFP, and sulfur cathode cells represented in green, orange, and red, respectively.

Figure 3b shows the yearly trends in the electrolyte materials and their corresponding capacity retention. Over time, the range of investigated solvents and salts has expanded, and the capacity retention performance has improved. Ether- and carbonate-based solvents and salts, such as LiTFSI, LiNO_3_, and LiPF_6_, were the most frequently used. The network graph in Figure 3c further illustrates this trend through the observed combinations of solvents and salts. We investigated the relationship between solvents and salts in the context of cathode compatibility, focusing on oxidation stability and operational voltage requirements (Figure S11).

Merging text and cycle graph data enabled the identification of cathode-specific capacity trends. The initial capacities of NCM and LFP cathodes are significantly lower (∼150–200 mAh g^–1^) than that of the sulfur cathode (Figure 3d), which has a much higher theoretical specific capacity of 1675 mAh g^–1^ (Figure S12). The end-of-life (EOL) performance of the three cathodes is also compared, showing that LFP and NCM generally demonstrate better long-term stability than sulfur-based LMBs (Figure 3e). This aligns with the well-known limitation of the lithium–sulfur battery (LSB), which exhibits low cycling stability despite operating at a relatively low C-rate (Figure S13). This is attributed to polysulfide dissolution and shuttle effects, which lead to active material loss and side reactions over prolonged cycling.

### Machine Learning Results

From the material–property database obtained through our mining procedure, we quantified the material composition, operating conditions, and solvation structures to generate input features. Additionally, we constructed 579-dimensional input vectors by incorporating molecular characteristics with physicochemical descriptors obtained from RDKit.^25^ We employed machine learning to predict the initial capacity and the capacity at the 50th cycle and validated the predictions through experimental testing.

#### Prediction of Initial Capacity

The Random Forest (RF) model achieved the highest *R* ^2^ score of 0.75 and MAE of 10.68 mAh g^–1^ for predicting the initial capacity of NCM cathodes (Figure 4a, Table S5). A total of 150 data points was used, which were split into training and test sets at a 70:30 ratio. We performed SHAP analysis to interpret the model and investigate the material–property–performance relationship. Figure 4b shows the top 10 features ranked in terms of importance. Higher feature values (red dot) with a positive SHAP value indicate a positive correlation with initial capacity, and vice versa for a negative SHAP value.

**Figure 4:**
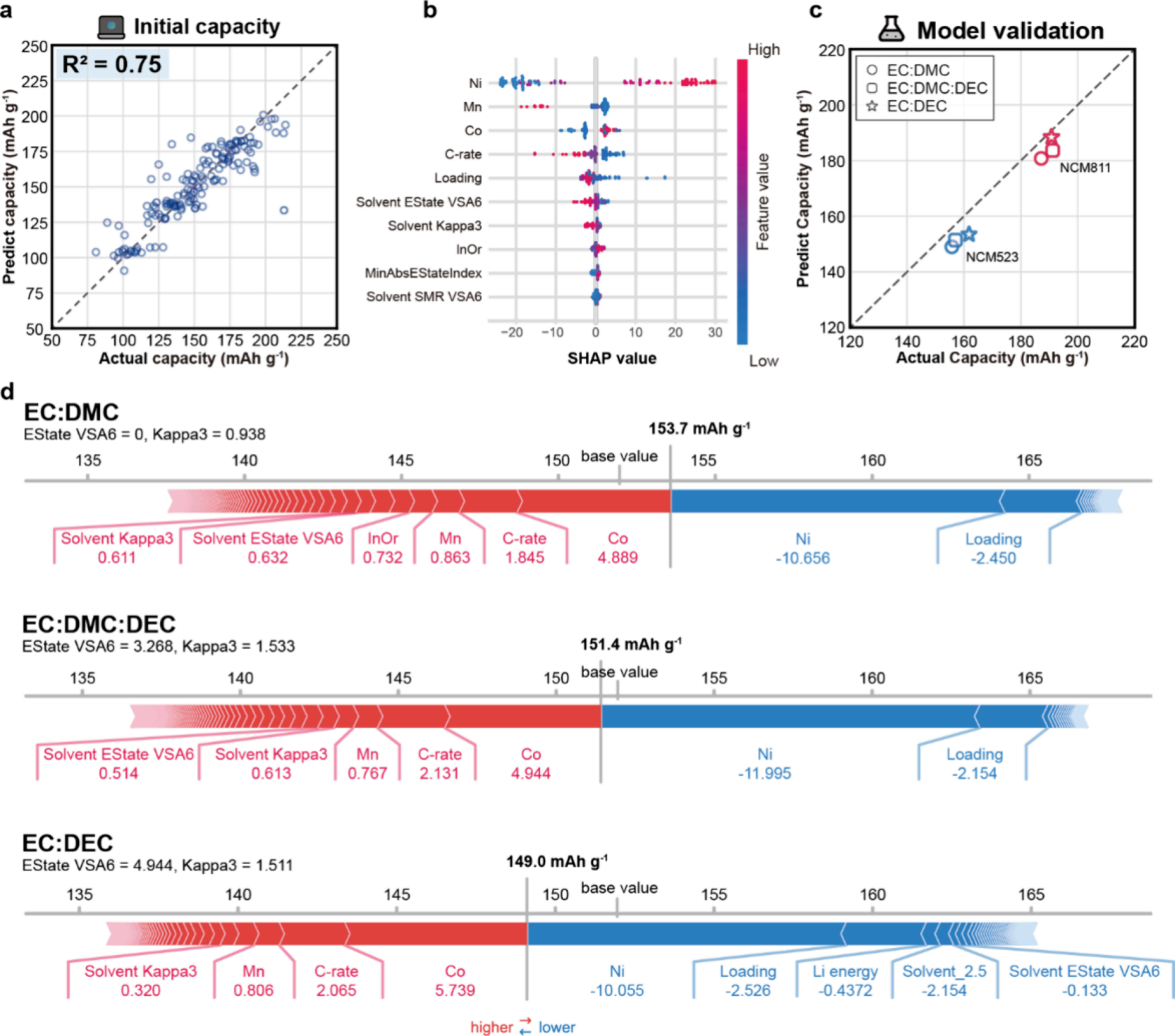
**Prediction of initial capacity and molecule structure analysis**. Result of initial capacities predicted from the random forest model with (a) the mined database and (b) SHAP analysis. (c) Experimental validation of the initial capacity of NCM523 and NCM811 cathodes with 1C-rate. Circle, square, and star symbols denote 1 M LiFSI in EC:DMC, EC:DMC:DEC, and EC:DEC, respectively. (d) Analysis of the system parameters for each different electrolyte system of the NCM 523 cathode (in part c). Red and blue arrows indicate that the feature positively and negatively contributes with initial capacity prediction. The value below each feature indicates the SHAP value.

From the SHAP analysis, the stoichiometric ratio of Ni, Mn, and Co of the cathode is the most influential input feature in the model. Redox reactions of transition metals directly involve electron transfer and primarily contribute to the capacity. Mn^4+^ is electrochemically inactive and therefore shows a negative correlation with capacity.^26^ This relationship is also observed in the ternary scatter plot of extracted NCM compositions with initial capacities (Figure S14), showing that Ni composition greater than 0.8 is associated with higher initial capacity. On the other hand, C-rate and cathode loading have a similar correlation because both influence overpotential. As the C-rate increases, overpotential arises from greater voltage loss due to internal resistance, increased Li^+^ concentration gradients in the electrolyte, and limitations in charge transfer kinetics. Increasing cathode loading, which increases its thickness, can lead to higher capacity because of an increasing amount of active material per unit area. However, this primarily leads to longer lithium diffusion paths, reduced electrolyte penetration into the cathode, and active material cracking, all of which hinder Li^+^ transport and contribute to additional overpotential. When the overpotential becomes severe, the cell may reach the cutoff voltage before sufficient charge transfer occurs, resulting in a reduction in initial capacity.

Beyond these features, the molecular characteristics of electrolytes also play a role in determining the initial capacity. Among them, EState VSA6 and Kappa3 show more pronounced dispersions in SHAP values. EStateVSA6 refers to the surface area of atoms that have EState values (representing electronic and topological atom-level information, Supporting Information, Note 4) between 1.54 and 1.81. Figure S15 shows that atoms within the EState VSA6 range tend to have near-zero partial charges, which arises from electron donation to neighboring oxygen atoms. This electron transfer results in enhanced polarity of the molecule (Figure S16). Kappa3 represents the structural complexity of the molecule and shows a positive correlation with EState VSA6 (Figure S17). In our SHAP analysis, low values of EState VSA6 and Kappa3 showed a positive correlation with initial capacity. Similar trends in SHAP analysis were observed under high C-rate and high Ni-cathode conditions as well (Figure S18).

To validate and analyze the model, we compared the experimental initial capacity results of battery cells with different cathode and electrolyte compositions (Tables S6 and S7). In Figure 4c, the experimental data show good agreement with the machine learning predictions. We compared the force plots of NCM523 cells with different electrolytes (1 M LiFSI in EC:DMC, EC:DMC:DEC, and EC:DEC, respectively) to investigate the effect of the electrolyte molecular structure (Figure 4d). The force plot shows how each feature influences the prediction for a specific example, including whether its effect is positive or negative. Specifically, the solvent EState VSA6 feature in the EC:DEC system, which has a high value (4.944), was found to exert a negative effect on the initial capacity. In contrast, other solvents show a positive effect, with high SHAP values observed as the EState VSA6 decreases. These results indicate that the polarity of the solvent affects Li^+^ transport and SEI formation during the initial stage of charge–discharge cycles.

#### Prediction of 50th Cycle Capacity

To overcome the difficulty of prediction beyond the initial cycle in lithium metal batteries, we developed a capacity prediction model for the 50th cycle (Figure 5a, Table S8). The Random Forest model achieved an *R* ^2^ score of 0.69 and MAE of 12.60 mAhg^–1^ in predicting the 50th cycle capacity of NCM cathodes. A total of 144 data points were used, which were split into training and test sets in a 70:30 ratio. As shown in Figure 5b, SHAP analysis revealed that key features such as cathode composition ratio, C-rate, and areal loading were consistently important, aligning with those identified in the initial capacity prediction. Interestingly, Li^+^ solvation features, such as Li^+^ cluster size and the fraction of solvent molecules within 1.9 Å of a Li^+^ (“solvent 1.9”), were also important in the prediction of the capacity. As the size of Li^+^ clusters increased or the proportion of solvent molecules surrounding Li decreased, which indicates an increased anion ratio, the predicted 50th cycle capacity also increased. This suggests that weakly solvated cells, characterized by larger Li^+^ clusters and fewer nearby solvent molecules, tend to exhibit higher 50th cycle capacities.

**Figure 5:**
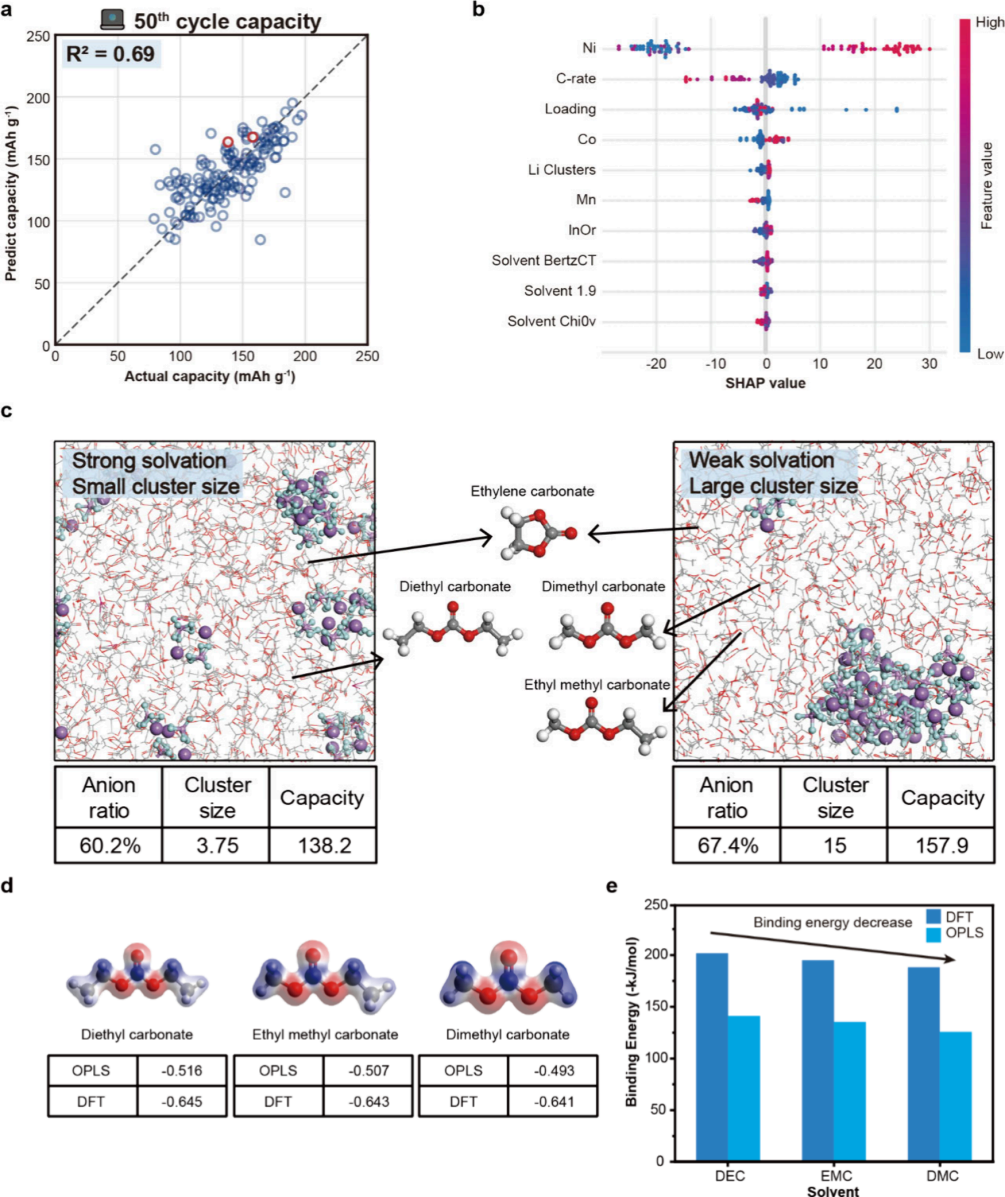
**Prediction of 50th capacity and solvation structure analysis**. Result of 50th cycle capacities predicted from the random forest model with the mined database (a) and (b) SHAP analysis. (c) Solvation structures from MD simulations of NCM811 cells with different solvents; oxygen, carbon, hydrogen, fluorine, phosphorus, and lithium atoms are shown in red, gray, white, cyan, purple, and violet, respectively. (d) Atomic charges of DEC, EMC, and DMC molecules from OPLS and DFT. (e) Li^+^–solvent binding energies for DEC, EMC, and DMC calculated via MD and DFT (red and blue indicate electron accumulation and depletion, respectively; the isosurfaces are shown at an isovalue of 0.05 e Å^–3^).

As illustrated in Figure 5c, we observed representative atomic configurations from molecular dynamics simulations in NCM811 cells prepared under identical measurement conditions but with different solvents (see the red data points in Figure 5a). In the cell using ethylene carbonate (EC) and diethyl carbonate (DEC), strong solvation was observed with small Li^+^ cluster sizes. In contrast, the cell containing ethylene carbonate along with dimethyl carbonate (DMC) and ethyl methyl carbonate (EMC) exhibited larger Li^+^ clusters and showed an improved electrochemical performance. To further investigate the origin of the observed solvation strength, we conducted additional calculations and analyses focusing on the intrinsic properties of the solvent molecules (Figure 5d,e). At the MD scale, we examined the Li^+^ binding energy and the partial charge of the O atoms at the binding sites for each solvent. Among the solvents, DEC exhibited the strongest binding energy and the most negatively charged O atoms, followed by EMC and DMC. These trends were also confirmed by DFT calculations, which showed consistent binding energies and charge distributions. The weaker Li^+^–solvent binding and more negatively charged O atoms in EMC and DMC indicate weak solvation interactions.

#### Validation of Electrolyte Design Insights

Building upon the insights obtained from the earlier stages of model development and material property analysis, we designed and evaluated new solvent systems exhibiting distinct molecular characteristics to validate our predictions experimentally. Specifically, three nonfluorinated ether solvents, diethyl ether (DEE), dipropyl ether (DPE), and diethylene glycol dimethyl ether (DEGDME), which were not part of the original training data set, were selected to explore the relationship between solvent properties and electrochemical performance. Compared to DEGDME, DEE and DPE exhibited substantially lower values for the polarity-related descriptors EState VSA6 and Kappa3. Specifically, DEE measured at 0 and 3.96, while DPE showed 0 and 5.96, respectively, in contrast to DEGDME’s 14.22 and 7.88. These results indicate that DEE and DPE have polarities significantly lower than that of DEGDME (Figure 6a). According to DFT calculations, the Li^+^ binding energy in DEE (−188 kJ/mol) and DPE (−196 kJ/mol) was significantly weaker than that in DEGDME (−356 kJ/mol), which is attributed to DEGDME’s higher density of O atoms capable of coordinating with Li^+^ (Figure 6b). This indicates the solvation power follows the trend DEE ≈ DPE < DEGDME.

**Figure 6:**
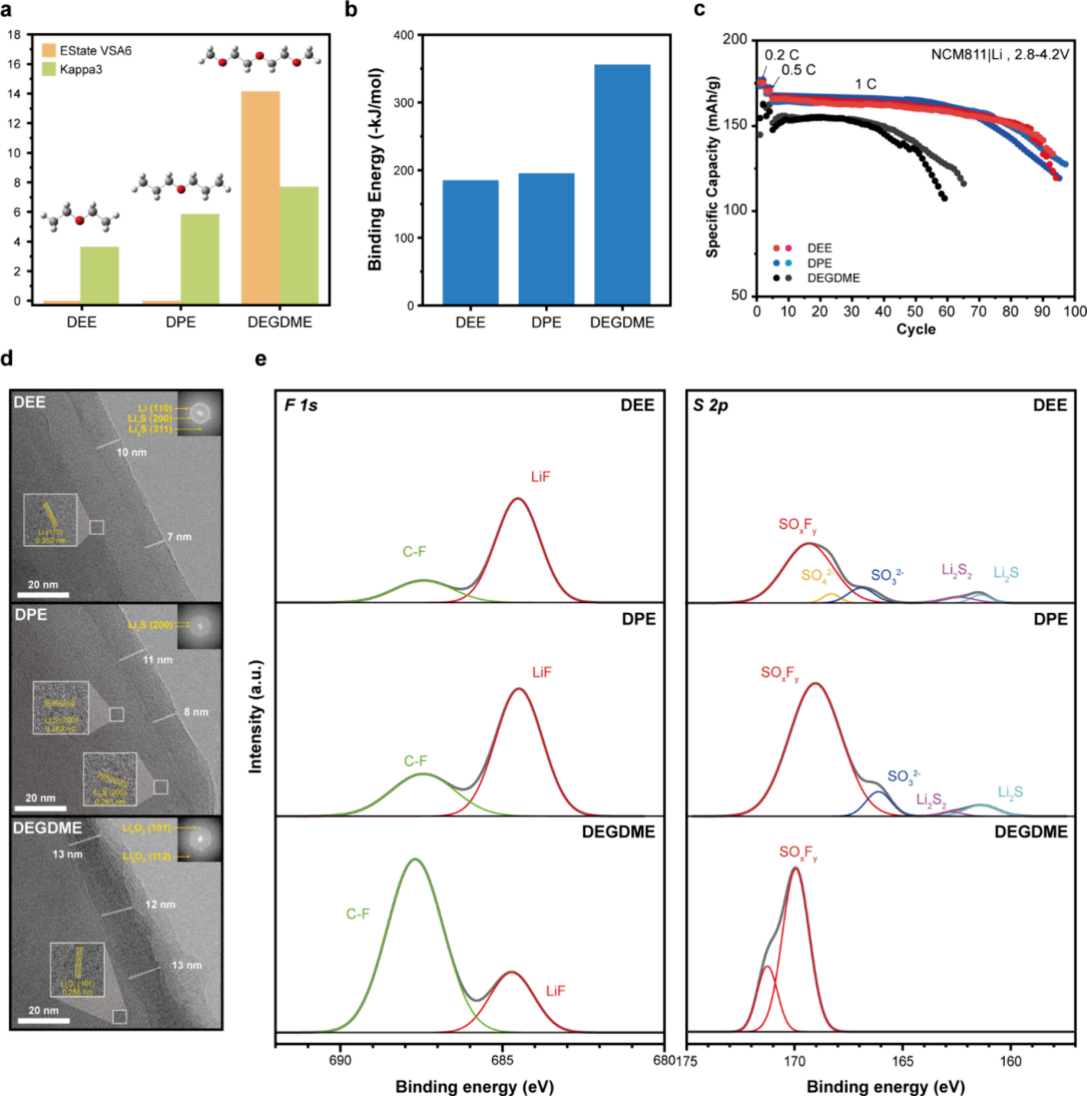
**Solvation effects on the cyclability and SEI morphology**. (a) The molecular features (EState VSA6 and Kappa3) and structures of the solvents. (b) The Li^+^ binding energies of the solvents. (c) Cycling performance of Li||NCM811 coin cells at 1C-rate with 2 M LiFSI DEE, 2 M LiFSI DPE, and 2 M LiFSI DEGDME. Two precycles at 0.2 and 0.5C were performed. (d) Cryo-TEM images obtained from SEI layers on Li-metal deposit (1C), with the corresponding fast Fourier transform (FFT) images shown in the insets. (e) Deconvoluted XPS spectra of F 1s and S 2p signals from the SEI layers of cells cycled with DEE, DPE, and DEGDME electrolytes.

Given the lower polarity and weaker solvation ability of DEE and DPE compared to DEGDME, we hypothesized that DEE and DPE would exhibit superior electrochemical performance. To validate this prediction, Li||NCM811 coin cells were prepared to evaluate the electrochemical performance of each electrolyte, all formulated with 2 M LiFSI. Electrochemical testing revealed that the DEE and DPE electrolyte delivered a higher initial capacity and more stable cycling performance at a current density of 1C. In contrast, cells with the DEGDME exhibited a rapid capacity fade, reaching 80% of the initial capacity within a relatively short cycle life (Figure 6c). This trend was also observed at a higher current density of 5C (Figure S19).

Cryogenic transmission electron microscopy (Cryo-TEM) was used to investigate the SEI morphology of each electrolyte battery cell. In Figure 6d, the DEE and DPE electrolytes facilitated the formation of uniform and compact SEI layers containing Li_2_S species with a thickness of approximately 8 and 9 nm, respectively. In particular, the homogeneous SEI formed in DEE electrolyte enables well crystallized Li plating to be observed. In contrast, the DEGDME electrolyte produced a rough and thicker (∼13 nm) SEI layer containing Li_2_O_2_ species. X-ray photoelectron spectroscopy (XPS) was performed to characterize the chemical species of the SEI layer in each cell. In the DEE and DPE electrolytes, the full reduction of LiFSI resulted in higher LiF and Li_2_S/Li_2_S_2_ peaks in the F 1s and S 2p spectra, which are well passivating layers, respectively (Figure 6e). Combined with the FFT analysis (Figure 6d), the LiF phase was identified as amorphous, which promotes high Li^+^ conductivity and stable lithium plating.^27,28^ In contrast, the DEGDME electrolyte exhibited pronounced C–F peaks in the F 1s associated with the partial reduction of LiFSI. These results support the idea that low EState VSA6 and weakly solvating electrolyte induced the formation of anion derived SEI components. This indicates that polar (high EState VSA6) and strongly solvating electrolytes, due to the presence of numerous organic solvent molecules coordinated with Li^+^, are ineffective in passivating the Li anode. Consequently, uniform Li plating and stripping are hindered. EState VSA6, a molecular property, was found to influence both the solvation ability of the electrolyte and the formation of the SEI, thereby enabling a stable performance in LMBs. These findings not only demonstrate the material–performance correlations from the LLMB agent but also provide insight into the underlying phenomena.

### Extension to Lithium Sulfur Batteries

To extend our data-driven methodology to LSB using mined data, we considered the unique structural complexity of these systems. In LSB, sulfur is incorporated into host materials, and the structure and properties of these hosts critically influence the capacity performance. However, representing the diverse composite structures of host materials as machine learning inputs remains a significant challenge. As with previous approaches, excluding host material information and relying solely on other battery components proved to be insufficient for accurately predicting specific capacities in LSBs. Therefore, we included the initial capacity of the LSB cell as an input feature, offering a meaningful proxy of the host material characteristics. This approach enabled accurate prediction of long-term specific capacities at the 100th, 200th, and 300th cycles (Figure 7a, S21). For the 200th cycle prediction, we used a data set of 752 LSB battery entries, split into training and test sets at a 70:30 ratio. The Gradient Boosting Regressor (GBR) model achieved an *R* ^2^ score of 0.71 and an MAE of 84.59 mAhg^–1^ for the 200th cycle. The model also demonstrated a strong performance at the 100th and 300th cycles (Figure S21). The superior predictive performance of LSBs at specific cycles can be attributed to the model’s ability to learn the degradation behavior associated with the shuttle effect. To mitigate the shuttle effect,^29^ lithium polysulfide migration is typically suppressed by modifying the battery separator.^30–32^ However, the lack of a method for representing modified separators limited their direct inclusion in the model. Instead, we used only cells with standard separators, which are known to exhibit a dominant shuttle effect that the model appears to have effectively captured.

**Figure 7:**
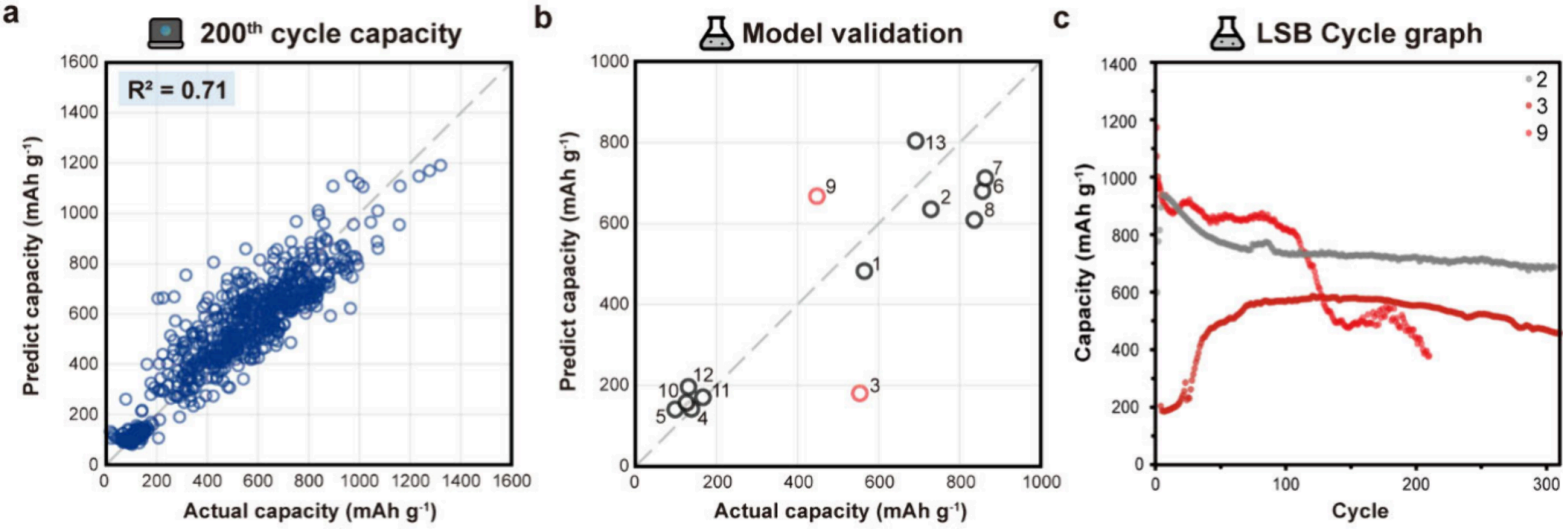
**Prediction and experimental validation of the 200th cycle capacity in LSB batteries**. (a) Result of 200th capacity prediction from the GBR model using LSB batteries. (b) Experimental validation results comparing predict and actual capacities; red data points indicate outliers. (c) Cycle performance graph of representative batteries, highlighting the outlier cells (red) and normal cell (gray).

To evaluate the model, we conducted cycle tests on 13 different LSB cells with varying cathodes, electrolyte amounts, and C-rates, which were not included in the model training data (Table S9). As shown in Figure 7b, the predictions of our models show good agreement with the experimental results. Interestingly, cell indices 3 and 9 (marked with red circles) exhibit a lower prediction accuracy. Upon examining their cycle profiles (Figures 7c and S22), we observed unstable behavior such as abrupt changes in slope and prolonged activation periods, suggesting that assembly related issues may have contributed to these anomalies.^33^ It is worth noting that such irregularities frequently occur in real data but are rarely documented in the literature, which is why they are missing from the text-mined data set.

## Discussion

In this study, we developed an LLMB agent to automatically extract battery information from research articles. We overcame key challenges in device-level data extraction, including low accuracy, difficulty in extracting performance data from graphs, and the lack of a device-level mining framework, by integrating text and graph mining models through a hierarchical LLM architecture. Using the extracted data, we developed an early prediction machine learning model capable of predicting battery capacity based on material composition and operating conditions prior to cycling. Importantly, LLMB enabled the transformation of raw data into insights regarding the relationships among electrolyte properties, capacity, and Li plating behavior. We found that low-polarity, weakly solvating solvents increased the initial capacity and promoted stable and crystalline Li plating, which was also confirmed through experimental validation. Our workflow from data extraction to electrochemical analysis demonstrates that the LLMB agent functions not merely as a data mining tool but as a foundation for data-driven battery material discovery. Moreover, the predictive capability of this framework can be further strengthened by increasing the availability of well-curated and comprehensive data sets. Several features extracted by LLMB were too sparsely reported to be incorporated into the machine learning model; for example, Li metal anode-related parameters were identified but were insufficient in number of robust models which are known to critically affect battery performance.^34^ More standardized reporting and complementary data generation through self-driving laboratories would enable the acceleration of materials innovation across a broad range of scientific domains.

## Methods

### Elsevier Journal Paper Crawling

This study focused on scientific journals from Elsevier publisher. Using the Elsevier Scopus API, we initially retrieved a total of 7,342 lithium metal battery (LMB)-related papers through keyword-based searches using ‘TITLE-ABS-KEY (“Li metal battery” OR “Lithium metal battery” OR “LMB” OR “Lithium metal anode” OR “LSB” OR “Lithium sulfur battery”) AND (LIMIT-TO (DOCTYPE, “ar”))’ keyword. From this data set, 898 papers that were unrelated to our target topics, namely, NCM, LFP, sulfur cathodes, and liquid electrolytes, were excluded. Additionally, 2,838 papers that did not include cycling tests of battery cells were also removed. As a result, 3,606 relevant papers were selected and used as input for the LLMB agent. Each paper was downloaded in XML format, in compliance with the data usage policies set by Elsevier journals.

We note that restricting the corpus to Elsevier journals may introduce publisher related stylistic or formatting characteristics that are not necessarily representative of the broader battery literature. Therefore, interpretations of the model performance and generalizability should be understood within the context of the selected corpus.

### Prompt Engineering

LLM is a trained artificial intelligence (AI) system capable of understanding the context in which language is used and completing natural language processing tasks by applying learned patterns. Prompt engineering has emerged as a crucial strategy for tailoring language models to specific objectives and enhancing their performance.^35,36^ This process involves the creation of advanced prompts designed to extract highly accurate battery information from research articles. We have developed an innovative set of prompts to decompose and reconstruct battery components, which are integrated with the LLM via Python code.

All prompts are constructed using an objective–question–explanation–exemplar structure, enabling precise problem-solving and ensuring the output format meets specific requirements. During all extraction stages, the LLM uses detailed explanations in the prompts to gain a comprehensive understanding of the battery device and accurately identify the relevant classes, properties, or materials from the target paragraph. In the merging stage, the process involves using text mining and graph mining results to identify battery cell information within the cycle graphs. A key aspect of our prompt engineering is that the outcomes from each sequential stage are passed on to the next stage, facilitating the rapid and efficient generation of progressive mining results. Each prompt is reinforced with two to four examples to enhance the accuracy of the LLM in generating responses.

### Graph Mining: Material Graph Digitizer

MatGD is designed to identify and eliminate objects such as text, legends, and arrows from graphs, based on the YOLOv8 model architecture. It employs the DBSCAN algorithm to segregate the remaining data lines based on their RGB color vector. However, this approach imposes limitations on MatGD’s capability to digitize certain types of graphs accurately. For example, inaccuracies occur when a marker from a legend, which should be removed as part of the graph objects, is not deleted and is mistakenly extracted as a data point. Additionally, challenges arise when different battery cells are depicted in the same color but are differentiated by scatter point shapes, or when the RGB values of the original graph’s data lines are similar, leading to improper separation. As a result, such graphs are excluded to ensure that only accurate data is extracted.

To eliminate Coulombic efficiency (CE) data and isolate the cyclability data, specific identification methods were used. When the label for CE was present in the graph legend and detected through text recognition, the corresponding CE data were removed directly. In cases where the CE label was absent, tick labels or axis labels on the right side of the graph box were used as indicators to determine the presence of CE data. A threshold was set based on the observation that CE values typically positioned above the cycle graph line. By using this threshold, CE data could be effectively identified and eliminated, allowing for isolation of the capacity data line.

### Feature Standardization

Despite the identical chemical compositions or materials, authors in various studies often describe them differently, making a standardized process essential. During this standardization process, materials amenable to SMILES format conversion, including binders, conductive materials, electrolyte salts, electrolyte solvents, and separators, were transformed.

Subsequently, the units of extracted features, including the thickness of lithium anodes, temperature, current density, and loading of active materials, were standardized using rule-based Python code to harmonize these units and convert associated values. This unit standardization was also applied to the concentration of the electrolyte salt and the ratio of the electrolyte solvent. For consistent representation of electrolyte concentrations, different metrics like molarity, molality, and mass percentage were converted, with additional information such as molecular weight and density extracted using SMILES.

### Computational Details

All quantum chemical calculations were performed by using the Gaussian 09 software package. Geometry optimizations and binding energy calculations were carried out at the B3LYP level of theory with the 6-311+G­(d,p) basis set. Natural population analysis (NPA) was performed to evaluate the atomic charges and electronic distributions.

Classical molecular dynamics simulations were carried out using LAMMPS software with the OPLS-AA force field to describe interatomic interactions. Each system was constructed with electrolyte species at concentrations extracted through the data mining of experimental systems. The systems were relaxed via energy minimization to remove unfavorable contacts, followed by equilibration in the NPT ensemble at 300 K to stabilize the temperature and pressure. They were then annealed by heating to 450 K, held at that temperature, and subsequently cooled back to 300 K to ensure structural relaxation. After equilibration, production runs were performed in the NVT ensemble at 300 K for 10 ns with a time step of 1 fs and key structural and dynamical properties were analyzed.

### Feature Generation and Machine Learning

For our machine learning analyses, we utilized the merged database previously described. The active materials, conductive additives, binders, salts, and solvent materials were all converted into SMILES notation and encoded into one-hot vectors, with the presence or absence of these materials indicated using binary values (1 for presence and 0 for absence). These features are listed in Table S4. Additionally, molecular characteristics of electrolyte salts and solvents were extracted and transformed into features by using the RDKit module.

Our machine learning models included only pure Celgard separators in the data set due to the challenges of representing modified separators, such as doped or mixed Celgard, in SMILES format. Additionally, we restricted our analysis to battery cells operated at room temperature (RT cells), resulting in a data set that includes 1,736 battery cell data points.

Multiple machine learning models were evaluated using the PyCaret library to enable a systematic and fair comparison under identical conditions. Because the data set contains mixed numerical and categorical features with strong nonlinear interactions, tree-based models consistently demonstrated superior performance. Accordingly, Random Forest (RF), XGBoost, and Gradient Boosting Regressor (GBR) were selected as representative models for this study. All models were trained with a 10-fold cross-validation.

### Materials and Electrochemical Measurements

Lithium metal foils (250 μm) and carbon coated Al foils were purchased from MTI Corporation. DME, EC, DEC, DMC, DEE, DEGDME and dehydrated solvents and lithium bis­(trifluoromethanesulfonyl)­imide (LiTFSI, 99.99%), lithium bis­(fluorosulfonyl)­imide (LiFSI, >99.9%), lithium difluoro­(oxalato)­borate (LiDFOB), lithium nitrate (LiNO_3_, 99.9%), and lithium hexafluorophosphate (LiPF_6_, 99.9%) salts were purchased from Sigma-Aldrich. DPE and lithium bis­(pentafluoroethanesulfonyl)­imide (LiBETI, 98.0%) were purchased from Tokyo Chemical Industry Co. All electrolytes used in each machine learning model were prepared by dissolving specific amounts of salt and solvent in an Ar-filled glovebox (O_2_ < 0.1 ppm, H_2_O < 0.1 ppm). In initial capacity validation, we used NCM523 [(3.4 mAh/cm^2^) NCM523:Super P C65:polyvinylidene fluoride (PVDF) = 94:3:3] and NCM811 [(2.2 mAh/cm^2^) NCM811:Super P C65:PVDF = 96:2:2] cathodes, which were purchased from Welcos Ltd. Areal loadings of active materials for NCM 523 and 811 were 21.5 and 12 mg/cm^2^, respectively. The cathode used for long-term cycling and SEI analysis was NCM811 [(1.8 mAh/cm^2^) NCM811:Super P C65:PVDF = 96:2:2] with 11 mg/cm^2^ area loading, purchased from Welcos Ltd. To prepare sulfur cathodes, melt diffusion was employed using 70 wt % sulfur (Sigma-Aldrich) and 30 wt % carbon host, either Super P carbon (SP, Thermo Fisher) or carbon nanotube (CNT, Carbon Nanomaterial Technology Co.), at 155 °C for 12 h. Subsequently, we mixed a sulfur/carbon composite with SP conductive additive and PVDF (*M*_w_ ∼ 534,000, Sigma-Aldrich) binder in a specific mass ratio. This mixture was dispersed in *N*-methyl-2-pyttolidone solvent (Sigma-Aldrich) to form a slurry. We then bar-coated (Welcos Ltd.) slurry onto carbon coated Al foil, which was dried in a vacuum oven at 60 °C for 8 h.

All coin-type cells (CR2032) were assembled with a 14 mm diameter of cathode, PP separator (Celgard 2400, Welcos Ltd.) and a 16 mm diameter of Li anode in an Ar-filled glovebox (O_2_ < 0.1 ppm, H_2_O < 0.1 ppm). The amount of injected electrolyte was adjusted to a specific E/A ratio according to the experimental conditions. The galvanostatic charge/discharge cycle tests were conducted within a voltage window of 2.8–4.2 V (NCM) and 1.7–2.8 V (Sulfur) at room temperature using a WBCS3000L instrument (WonATech Ltd.).

### Cryogenic Transmission Electron Microscopy

To minimize damage during SEI morphology analysis on Li, cryo-TEM was performed using a Krios G4 (ThermoFisher Scientific, 300 kV) microscope. During cell assembly, a 400-mesh copper TEM grid (agar scientific) was placed on the top of the Li metal anode. Following lithium plating at 1C-rate, cells were disassembled in the Ar glovebox. The grid was rinsed with the respective solvent to remove excess salts and dried in the glovebox (∼120 s). For the cryo-TEM sampling, the TEM grid was sealed into the conical tube, immediately moved out of the Ar glovebox, and immersed in a liquid nitrogen filled cryo-autoloader. All of the TEM images were converted to the FFT images through Gatan Microscopy Suite software.

### X-ray Photoelectron Spectroscopy

XPS was performed to characterize the element spectra of the SEI layer. The XPS sample was prepared on the TEM grid in the same way as cryo-TEM. After disassembling the cells in the Ar glovebox and rinsing the grid with the selected solvent, the grid was sealed under an Ar atmosphere into the vials and transferred to the XPS vacuum holder. XPS measurement was performed using a Nexsa G2 instrument (ThermoFisher Scientific) equipped with a monochromatic Al Kα radiation source.

## Supplementary Material





## Data Availability

The LLMB model is available at https://github.com/skyljw0714/LLMB.

## Notes

The LLMB data mining methodology; tables summarizing prompt robustness, extracted/standardized data, ML features and performance (including DoE and experimental validation), descriptor definitions, missing field statistics, DFT derived molecular data, and external validation; figures showing the LLMB architecture, extraction schematics, data set distributions, topic modeling and electrolyte analyses, descriptor correlations, SHAP results, cycling/prediction validation, and database curation/coverage (PDF)
Transparent Peer Review report available (PDF)
