# Supplementary material for: LLMB: AI Agent for Lithium Metal Battery Research Using Large Language Model
Source: ACS Cent Sci. 2026 Mar 17;12(4):484–96. doi: 10.1021/acscentsci.5c02433 (PMC13107216; doi:10.1021/acscentsci.5c02433)
Supplement: Supplementary file 1 [file oc5c02433_si_001.pdf]

Supplementary information

LLMB: AI agent for lithium metal battery research using large language model

Jaewoong Lee[1,†], Junhee Woo[2,†], Younghun Kim[1], Sejin Kim[1], Cinthya Paulina[1,2], Hyunmin Park[2], Hee-Tak Kim[1,], Steve Park[2,*] and Jihan Kim[1,*]*

[1] Department of Chemical and Biomolecular Engineering, Korea Advanced Institute of Science and Technology, Daejeon, 34141, Republic of Korea

[2] Department of Materials Science and Engineering, Korea Advanced Institute of Science and Technology, Daejeon, 34141, Republic of Korea

† These authors contributed equally: J. Lee, J. Woo

*: Corresponding author

Corresponding author Email: Jihankim@kaist.ac.kr (Jihan Kim), stevepark@kaist.ac.kr (Steve Park), heetak.kim@kaist.ac.kr (Hee-Tak Kim)

Table of Contents

Supplementary Note S1. Major and sub categorization in text mining

Supplementary Note S2. Data extraction matrix of graph mining

Supplementary Note S3. Interlayer extraction

Supplementary Note S4. Electrotopological State

Supplementary Note S5. Graph mining details

Supplementary Note S6. Relevant paragraph extraction

Supplementary Table S1. Evaluation of prompt robustness

Supplementary Table S2. Example result of text mining and unit, SMILES standardization

Supplementary Table S3. Evaluation metrics of text mining by LLMB

Supplementary Table S4. Features used for machine learning

Supplementary Table S5. Evaluation metrics of various machine learning models predicting initial capacity

Supplementary Table S6. DoE table in initial capacity prediciton model

Supplementary Table S7. Experimental initial capacity results

Supplementary Table S8. Evaluation metrics of various machine learning models predicting 50th cycle capacity

Supplementary Table S9. DoE table in capacity at target cycle prediciton model

Supplementary Table S10. Definitions of descriptors used in the SHAP analysis

Supplementary Table S11. Summary statistics of missing fields in text mining data

Supplementary Table S12. Natural atomic charges (NPA) and Cartesian coordinates of DEC, EMC, and DMC molecules

Supplementary Table S13. Comparison of values at 5, 50, 100, 200, and 300 cycles between experimental data and MatGD extracted data

Supplementary Table S14. Performance comparison of predicting models for initial capacity and 50th cycle capacity with and without inclusion of DFT derived descriptors

Supplementary Table S15. External validation of the text mining error across different publishers

Supplementary Figure S1. Schematic illustration of the LLMB architecture

Supplementary Figure S2. Schematic illustration of material extraction results using LLM with detailed Major- and Sub-categorization of method texts

Supplementary Figure S3. The prompt example for the cycle graph extraction model

Supplementary Figure S4. Schematic illustration of overall text mining results using LLMB

Supplementary Figure S5. Data distribution of cathode

Supplementary Figure S6. Histogram graphs of electrolyte

Supplementary Figure S7. Histogram graphs of Celgard separators

Supplementary Figure S8. Histogram graph of Li metal anode thickness

Supplementary Figure S9. Histogram graphs of operating conditions

Supplementary Figure S10. Top-20 most relevant terms for topic electrolyte, cathode, anode, separator, and current collector from Latent Dirichlet Allocation

Supplementary Figure S11. The heatmaps illustrate the relationship between salt and solvent pairs in the electrolytes of NCM, LFP, and LiS cathode cells

Supplementary Figure S12. Data distribution of each cycle capacity at different cathode type

Supplementary Figure S13. Data distribution of C-rate at different cathode type

Supplementary Figure S14. A ternary plot of the distribution of Ni, Co, and Mn stoichiometry ratio

Supplementary Figure S15. Partial charge distribution in solvent molecules

Supplementary Figure S16. Correlation between MolLogP and Estate VSA6 solvent molecular descriptors

Supplementary Figure S17. Scatter plot showing the relationship between Estate VSA6 and solvent Kappa3

Supplementary Figure S18. SHAP feature analysis of initial capacity prediction for NCM cathode with a high C rate, and high Ni content conditions

Supplementary Figure S19. Cycling performance of Li||NCM811 coin cells at 5C-rate

Supplementary Figure S20. Cycling performance of Li||NCM811 coin cells at 1C-rate with 2M LiFSI in DME

Supplementary Figure S21. Capacity prediction and experimental validation of the 100th and 300th cycle in LSB batteries

Supplementary Figure S22. Cycle graph of lithium sulfur batteries in various experimental conditions

Supplementary Figure S23. Comparison between experimental data and MatGD extracted data

Supplementary Figure S24. Data curation funnel for constructing the LLMB database

Supplementary Figure S25. Data distribution of published research papers about lithium metal battery over the last 20 years. Entries for 2024 included until January 2024

Supplementary Figure S26. Data coverage across the journals and chemistries in machine learning

Supplementary Figure S27. SHAP plot of 200th cycle capacity prediction model for lithium sulfur battery

Supplementary Notes

Supplementary Note S1. Major and sub categorization in text mining

In the major categorization, paragraphs are grouped into one of four categories: material information, synthesis, operating condition, and other. Only the paragraphs classified as material information or operating condition, which contain relevant information on battery material components, are retained for further processing. In the subsequent sub-categorization, each remaining paragraph is further classified based on the specific battery cell component or operational condition it describes, thereby preparing the data for value extraction. The model assigns one or more of the following labels: cathode, anode, electrolyte, separator, current collector, or operating condition.

Supplementary Note S2. Data extraction matrix of graph mining

It is impractical to use the first data point from all cyclability graphs as the initial capacity due to inherent variability in the conditions under which the data was collected. Specifically, some graphs include an activation process conducted at low C rates, while others do not. Distinguishing between these conditions based solely on the graph's shape proved challenging. Instead, we utilized the capacity value from the fifth cycle as the initial capacity. This approximation is warranted as the activation process typically concludes within the first five cycles, resulting in a saturated initial capacity value. Moreover, for graphs visualizing cyclability after activation, the capacity values at the first cycle and the fifth cycle are nearly identical, affirming the reliability of this approach.

Supplementary Note S3. Interlayer extraction

Additionally, we also extracted interlayer information using the same text mining strategy. If interlayer is in battery cell, it was extracted as material name, and stated in the prompt not to be confused with other materials. However, in the accuracy test set, the only 44 out of 384 battery cells contain interlayer in the test set, which is very small data. Among them, 8 cells were extracted wrong interlayer, resulting in a relatively low F1 score of 0.900 (Supplementary Table 7). Even though several examples have been given in the prompt engineering, there is still multilayer separator, solid-electrolyte interlayer (SEI), doping layer, and other components referred to as interlayer was believed to be caused by the broad definition of ‘Interlayer’.

Supplementary Note S4. Electrotopological State (EState)

EState is a molecular descriptor, which quantitatively represent the electronic and topological environment of each atom within a molecule. It captures how the intrinsic electronic character of an atom in modified by its bonding context and molecular connectivity. The EState, denoted as S_i, index for atom i is defined as follow Equation (1).

$$\mathbf{S}_i = \mathbf{I}_i + \sum i{\neq}j \frac{I_i - I_j}{r_{ij}^2} \quad \textbf{(1)}$$

I_i is the intrinsic state of atom i, and r_{ij} represents the topological distance between atoms i and j (in number of bonds). This relationship implies that the degree of electronic asymmetry of atom i with respect to the surrounding atoms in the molecule.

The intrinsic state I_i is the inherent electronic configuration of an atom, as defined by Equation (2).

$$I_i = \frac{\left(\frac{2}{L_i}\right)^2 \times \delta_i^v + 1}{\delta_i} \quad \textbf{(2)}$$

δ_i is the σ-bond order, the total number of sigma bonds formed by atom i, δ_i^v is the number of valence electrons of the atom i, and L_i is the shortest bond distance from the atom i to the most distant atom within the molecule.

The EState value is combined both local intrinsic state (through I_i) and global molecular topology into a single numerical measure. A larger S_i atom indicates electronically richer and tends to be located near the molecular center, implying higher chemical reactivity and strongly influenced by electronegative or conjugated environments. On the other hands, a lower S_i atom is more electron deficient of the molecule.

Supplementary Note S5. Graph mining details

To quantitatively evaluate the accuracy of the graph mining procedure, we performed a direct validation using cyclability data generated in-house during the lithium metal battery experiments reported in this study. Cyclability graphs obtained from these experiments were processed using the same graph mining workflow (MatGD) applied to the literature data. The extracted values were compared with the corresponding ground-truth experimental capacities at selected cycle numbers. A representative comparison between the experimental curves and the graph mining data is shown in Supplementary Figure 23.

For three independent cells, capacity values at the 5th, 50th, 100th, 200th, and 300th cycles were extracted from the graphs and directly compared with the original experimental data. As summarized in Supplementary Table 13, the graph mining data showed excellent agreement with the ground-truth data across all tested cycle numbers and cells, with only minor deviations, typically within a few mAh/g.

Supplementary Note S6. Relevant paragraph extraction

Relevant paragraphs are extracted using section specific procedures. In the Methods section, paragraphs are directly retrieved from structured Elsevier XML files based on predefined section headers to obtain the corresponding methodological descriptions. In the Results section, a rule based paragraph selection procedure is applied to identify paragraphs that reference the figure labels associated with the target cell's cyclability graph, such as "Fig. 2," "Figure 2," or "Fig. 2a." Paragraphs containing these references are extracted as relevant textual information for subsequent analysis.

	Evaluation metrics			
	Accuracy	Precision	Recall	F1 score
**Prompt 1** (Information ○ / Example ○)	0.946	0.946	1	0.972
**Prompt 2** (Information ○ / Example ×)	0.794	0.931	0.931	0.931
**Prompt 3** (Information × / Example ○)	0.688	0.647	0.971	0.776
**Prompt 4** (Information × / Example ×)	0.679	0.905	0.679	0.776

Supplementary Table S1. Evaluation of prompt robustness across different prompt structures

To evaluate prompt robustness, we compared extraction accuracy under four prompts: (1) full prompts containing role specification, explanatory description, and examples, (2) prompts without examples, (3) prompts without battery specific explanatory text, and (4) prompts without both examples and explanatory text. We compared accuracy across a random set of 20 papers. The results showed a consistent decrease in performance as elements were removed, with full prompt structure yielding the highest accuracy. In Prompt 2 (without examples), frequent formatting errors led to a lower recall of 0.931. In Prompt 3 (without domain specific information), the model often extracted non-cycle graphs, resulting in a markedly low precision of 0.647. Prompt 4 (without both examples and information) suffered from both issues simultaneously. These demonstrate that while the framework remains functional with shorter prompts, including domain specific context and illustrative examples substantially improves reliability.

Figure 2a
1st cell

**Cell name**	$LiNO_3$ added electrolyte LSB coin battery (S loading: 1.0mg cm^{-2}) at 0.1C		
**Type**	**Property**	**Raw data**	**Preprocess data**
**Electrolyte**	name	LiNO3 added electrolyte	
	Salt	LiTFSI, $LiNO_3$	["[Li+].C(F)(F)(F)S(=O)(=O)[N-]S(=O)(=O)C(F)(F)F", "[Li+].[O-][N+]([O-])=O"]
	concentration	1M, 2wt%	value: [1, 0.363]<br>unit: ["M", "M"]
	solvent	DME, DOL	["COCCOC", "C1COCO1"]
	volume ratio	1:1	value: [0.5, 0.5]<br>unit: ["vol", "vol"]
	EA ratio	10	[10]
**Cathode**	name	sulfur cathode	
	active material	S, carbon black	["S", "carbon black"]
	ratio of active material	7:3	[0.7, 0.3]
	conductive additive	Super P	["[C]"]
	binder	PVDF	["C=C(F)F"]
	ratio of material weight	7:2:1	[0.7, 0.2, 0.1]
	area loading	1.0mg cm^{-2}	Value: [1.0],<br>unit: ["mg cm^{-2}"]
**Anode**	name	Li metal	
	thickness	0.25 mm	value: [250]<br>unit: ["µm"]
**Separator**	name	Celgard 2400	
	material	PP	["CC=C"]
**Current collector**	cathode collector	Al	["[Al]"]
	anode collector	Cu	["[Cu]"]
**Interlayer**	name	-	-

**Measurement condition**	C rate	0.1	[0.1]
	current density	-	-
	temperature	25°C	value: [25]<br>unit: ["°C"]

Supplementary Table S2. Example result of text mining and unit, SMILES standardization.

a

**Evaluation metrics**	**Cathode**						
	name	active material	ratio of active material	conductive additive	binder	ratio of cathode weight	area loading of active material
Accuracy	0.945	0.964	0.939	0.970	0.989	0.994	0.909
Precision	0.942	0.968	0.957	0.969	0.993	1.000	0.896
Recall	1.000	0.994	0.975	0.993	0.993	0.994	0.964
F1 Score	0.970	0.981	0.966	0.981	0.993	0.997	0.929

b

**Evaluation metrics**	**Electrolyte**							
	name	Salt material	Salt concentration	Salt concentration unit	solvent material	Solvent volume ratio	Solvent volume ratio unit	E/A ratio
Accuracy	0.992	0.992	0.992	1.000	0.992	0.981	0.981	0.930
Precision	0.991	0.990	0.990	1.000	0.990	0.989	0.989	0.791
Recall	1.000	1.000	1.000	1.000	1.000	0.986	0.986	0.815
F1 Score	0.995	0.995	0.995	1.000	0.995	0.988	0.988	0.803

c

**Evaluation metrics**	**Anode**			
	name	thickness	unit	additional layer
Accuracy	1.000	0.995	0.995	0.989
Precision	1.000	1.000	1.000	1.000
Recall	1.000	0.952	0.952	0.600
F1 Score	1.000	0.976	0.976	0.750

d **e**

Evaluation metrics	Separator
	material
Accuracy	0.968
Precision	0.958
Recall	1.000
F1 Score	0.979

Evaluation metrics	Current Collector	
	cathode CC	Anode cc
Accuracy	0.922	0.981
Precision	1.000	0.947
Recall	0.992	1.000
F1 Score	0.996	0.973

f

Evaluation metrics	Operating Condition				
	C rate	Current density	Current density unit	temperature	temperature unit
Accuracy	0.984	1.000	0.995	0.957	0.957
Precision	0.983	1.000	0.950	0.882	0.882
Recall	1.000	1.000	1.000	1.000	1.000
F1 Score	0.991	1.000	0.974	0.938	0.938

Supplementary Table S3. Accuracy, precision, recall, and F1 score of text mining by in **a** cathode, **b** electrolyte, **c** anode, **d** separator, **e** current collector, and **f** operating condition by LLM.

**Weight ratio of cathode active material**	**Weight ratio of cathode**	**Conductive additive (SMILES)**
**Binder (SMILES)**	**Loading amount**	**Salt (SMILES)**
**Solvent (SMILES)**	**Concentration of Salt**	**Volume ratio of solvent**
**Separator (SMILES)**	**C rate**	

Supplementary Table S4. Features used for machine learning.

Initial Capacity Prediction (150 NCM cathode cell)

Matrix	Initial capacity prediction		
	RF	GBR	XGBoost
MAE	10.22	10.31	9.64
R^2 Scores	0.78	0.75	0.78

Supplementary Table S5. MAE value and R^2 scores of various machine learning models (RF: Random Forest, XGBoost: Extreme Gradient Boosting, GBR: Gradient Boosting Regressor) for predicting initial capacity of NCM cathode cell. The reported values are averaged over models trained with three different random seeds.

Index	Cathode						Electrolyte					Anode	Separator	Condition	
	active material	ratio of active material	conductive additive	binder	ratio of cathode weight	area loading of active material	salt	salt concentration	solvent	solvent volume ratio	Electrolyte amount	thickness	separator	C rate	Temperature
1	NCM811	8:1:1	SP	PVDF	96:2:2	12 mg/cm^2	$LiPF_6$	1M	EC, DEC	1:1 v/v	50 µL	250 µm	Celgard 2400	0.5 C	25°C
2	NCM811	8:1:1	SP	PVDF	96:2:2	12 mg/cm^2	$LiPF_6$	1M	EC, DEC, DMC	1:1:1 v/v	50 µL	250 µm	Celgard 2400	0.5 C	25°C
3	NCM811	8:1:1	SP	PVDF	96:2:2	12 mg/cm^2	$LiPF_6$	1M	EC, DMC	1:1 v/v	50 µL	250 µm	Celgard 2400	0.5 C	25°C
4	NCM523	5:2:3	SP	PVDF	94:3:3	21.5 mg/cm^2	$LiPF_6$	1M	EC, DEC	1:1 v/v	50 µL	250 µm	Celgard 2400	0.5 C	25°C
5	NCM523	5:2:3	SP	PVDF	94:3:3	21.5 mg/cm^2	$LiPF_6$	1M	EC, DEC, DMC	1:1:1 v/v	50 µL	250 µm	Celgard 2400	0.5 C	25°C
6	NCM523	5:2:3	SP	PVDF	94:3:3	21.5 mg/cm^2	$LiPF_6$	1M	EC, DMC	1:1 v/v	50 µL	250 µm	Celgard 2400	0.5 C	25°C

Supplementary Table S6. Design of Experiments (DoE) table with experimental conditions in initial specific capacity prediction model.

Index	Initial specific capacity (mAh g^{-1})
1	187.8628
2	191.2006
3	191.3230
4	156.4594
5	157.4303
6	161.0640

Supplementary Table S7. Experimental actual values each condition in initial specific capacity prediction model.

50th Cycle Capacity Prediction (144 NCM cathode cell)

**Matrix**	**Initial capacity prediction**		
	RF	GBR	XGBoost
MAE	12.80	13.19	14.23
R^2 Scores	0.59	0.59	0.54

Supplementary Table S8. MAE value and R^2 scores of various machine learning models (RF: Random Forest, XGBoost: Extreme Gradient Boosting, GBR: Gradient Boosting Regressor) for predicting 50th cycle capacity of NCM cathode cell. The reported values are averaged over models trained with three different random seeds.

Index	Cathode						Electrolyte					Anode	Separator	Condition	
	active material	ratio of active material	conductive additive	binder	ratio of cathode weight	area loading of active material	salt	salt concentration	solvent	solvent volume ratio	EA ratio	thickness	separator	C rate	temperature
1	S, SP	7:3	-	PVDF	9:0:1	1.547 mg/cm^2	LiTFSI, $LiNO_3$	1M, 2wt%	DME, DOL	1:1 v/v	20	250 µm	Celgard 2400	0.2 C	25°C
2	S, SP	7:3	-	PVDF	9:0:1	1.371 mg/cm^2	LiTFSI, $LiNO_3$	1M, 2wt%	DME, DOL	1:1 v/v	20	250 µm	Celgard 2400	0.2 C	25°C
3	S, SP	7:3	-	PVDF	9:0:1	1.732 mg/cm^2	LiTFSI, $LiNO_3$	1M, 2wt%	DME, DOL	1:1 v/v	20	250 µm	Celgard 2400	1 C	25°C
4	S, CNT	7:3	SP	PVDF	7:2:1	2.737 mg/cm^2	LiTFSI, $LiNO_3$	1M, 2wt%	DME, DOL	1:1 v/v	10	250 µm	Celgard 2400	1 C	25°C
5	S, CNT	7:3	SP	PVDF	7:2:1	2.317 mg/cm^2	LiTFSI, $LiNO_3$	1M, 2wt%	DME, DOL	1:1 v/v	10	250 µm	Celgard 2400	0.2 C	25°C
6	S, CNT	7:3	SP	PVDF	7:2:1	3.242 mg/cm^2	LiTFSI, $LiNO_3$	1M, 2wt%	DME, DOL	1:1 v/v	10	250 µm	Celgard 2400	0.2 C	25°C
7	S, CNT	7:3	SP	PVDF	7:2:1	2.906 mg/cm^2	LiTFSI, $LiNO_3$	1M, 2wt%	DME, DOL	1:1 v/v	10	250 µm	Celgard 2400	0.2 C	25°C
8	S, CNT	7:3	SP	PVDF	7:2:1	3.622 mg/cm^2	LiTFSI, $LiNO_3$	1M, 2wt%	DME, DOL	1:1 v/v	7	250 µm	Celgard 2400	0.2 C	25°C
9	S, CNT	7:3	SP	PVDF	7:2:1	4.169 mg/cm^2	LiTFSI, $LiNO_3$	1M, 2wt%	DME, DOL	1:1 v/v	5	250 µm	Celgard 2400	0.2 C	25°C
10	S, CNT	7:3	SP	PVDF	7:2:1	5.136 mg/cm^2	LiTFSI, $LiNO_3$	1M, 2wt%	DME, DOL	1:1 v/v	3.5	250 µm	Celgard 2400	0.2 C	25°C
11	S, CNT	7:3	SP	PVDF	7:2:1	4.631 mg/cm^2	LiTFSI, $LiNO_3$	1M, 2wt%	DME, DOL	1:1 v/v	5	250 µm	Celgard 2400	0.2 C	25°C
12	S, CNT	7:3	SP	PVDF	7:2:1	4.758 mg/cm^2	LiTFSI, $LiNO_3$	1M, 2wt%	DME, DOL	1:1 v/v	3.5	250 µm	Celgard 2400	0.2 C	25°C
13	S, CNT	7:3	SP	PVDF	7:2:1	1.434 mg/cm^2	LiTFSI, $LiNO_3$	1M, 2wt%	DME, DOL	1:1 v/v	10	250 µm	Celgard 2400	1 C	25°C

Supplementary Table S9. Design of Experiments (DoE) table with experimental conditions in target cycles' specific capacity prediction model.

**Descriptor**	Explanation
**Ni**	The proportion of nickel within the cathode active material.
**Mn**	The proportion of manganese within the cathode active material.
**Co**	The proportion of cobalt within the cathode active material.
**C rate**	A measure of the charge or discharge current relative to the nominal capacity of a battery.
**Loading**	The amount of electrochemically active material coated on the electrode per unit area (mg/cm^2).
**Solvent EState VSA6**	MOE-type descriptors based on electrotopological state indices combined with surface area contributions as reported in J. Chem. Inf. Comput. Sci., 31, 76–81 (1991). The Estate VSA6 descriptor corresponds to the summed surface area of atoms whose EState values fall within the range $1.54 \leq \text{EState} < 1.81$.
**Solvent Kappa3**	A descriptor of molecular shape and branching complexity of solvent molecules, quantified by the Hall-Kier kappa3 value calculated using equations (58), (61), and (62) in Reviews in Computational Chemistry, Vol. 2, pp. 367-422 (1991).
**lnOr**	The inorganic-to-organic ratio, which quantifies the balance between inorganic species and organic solvent molecules in the solvation structure.
**Solvent MinAbsEStateIndex**	The minimum value of EState indices for the solvent molecules.
**Solvent SMR VSA6**	MOE-type descriptor based on molar refractivity and surface area contributions (6th bin, $2.75 \leq x < 3.05$), as reported in J. Mol. Graph. Mod., 18, 464–477 (2000).
**Li Clusters**	The average number of lithium atoms within lithium clusters in the solvation structure. A lithium cluster is defined as an aggregate of lithium atoms and surrounding anions or solvent molecules connected through Li-X interactions within a distance cutoff of 2.5Å..
**Solvent BertzCT**	A topological index of solvents that quantifies the structural complexity of solvent molecules. It consists of the sum of two terms: one representing bonding complexity and the other representing the complexity of heteroatom distribution.
**Solvent 1.9**	The fraction of solvent molecules within a 1.9 Å cutoff distance from a Li atom in the solvation structure.
**Solvent Chi0v**	A valence molecular connectivity index of solvent molecules calculated using equations (1), (9), and (10) from Reviews in Computational Chemistry, Vol. 2, pp. 367-422 (1991).

Supplementary Table S10. Definitions of descriptors used in the SHAP analysis.

**Field**	**Cathode type**	**Active material ratio**	**Cathode weight ratio**	**Conductive additive**	**Binder**	**Loading**
**# of missing entries**	2951	2951	1983	1891	2025	3664
**Field**	Li salt	Solvent	Li solvent concentration	Solvent volume ratio	Separator	C-rate
**# of missing entries**	2951	2951	1983	1891	2025	3664

Supplementary Table S11. Summary statistics of missing fields in text mining data.

Diethyl carbonate					
Atom	No	Atomic charge	X (Å)	Y (Å)	Z (Å)
O	1	-0.55437	1.083413	-0.406678	-0.000015
O	2	-0.55437	-1.083414	-0.406678	-0.000013
O	3	-0.64478	0	1.586134	0.000013
C	4	-0.03298	2.358872	0.28195	-0.000006
C	5	-0.03298	-2.358869	0.281949	-0.000001
C	6	-0.59434	3.443039	-0.775191	0.000016
C	7	-0.59434	-3.44304	-0.77519	0.000011
C	8	1.02589	0.000001	0.378253	-0.000013
H	9	0.18466	2.410454	0.922232	-0.883544
H	10	0.18466	2.410434	0.92225	0.88352
H	11	0.18466	-2.410435	0.922241	0.88353
H	12	0.18466	-2.410452	0.922239	-0.883533
H	13	0.20753	4.424225	-0.292815	0.000019
H	14	0.20715	3.371411	-1.40904	0.886413
H	15	0.20715	3.371428	-1.409059	-0.88637
H	16	0.20753	-4.424224	-0.29281	0.000015
H	17	0.20714	-3.371428	-1.409054	-0.886377
H	18	0.20714	-3.371419	-1.409044	0.886405
**Ethyl methyl carbonate**					
Atom	No	Atomic charge	X (Å)	Y (Å)	Z (Å)
O	1	-0.553	-0.645398	-0.441624	-0.000009
O	2	-0.54212	1.494817	-0.776633	-0.000004
O	3	-0.64299	0.734476	1.359117	0.000002
C	4	-0.03313	-1.800359	0.434878	0.000003
C	5	-0.59452	-3.033799	-0.443394	0.000008
C	6	1.02285	0.54422	0.166767	-0.000019
C	7	-0.20896	2.846619	-0.284957	0.000011
H	8	0.18497	-1.752568	1.075214	0.883658
H	9	0.18497	-1.75258	1.075221	-0.883648
H	10	0.20789	-3.929084	0.184102	0.000016
H	11	0.2075	-3.060467	-1.08072	-0.886378
H	12	0.2075	-3.060455	-1.080727	0.886388
H	13	0.18562	3.035255	0.317013	-0.889908
H	14	0.1878	3.473422	-1.173765	0.000027
H	15	0.18562	3.03523	0.317027	0.889925
**Dimethyl carbonate**					

Atom	No	Atomic charge	X (Å)	Y (Å)	Z (Å)
O	1	-0.54062	1.082852	-0.709587	-0.000074
O	2	-0.54062	-1.082853	-0.709586	-0.000072
O	3	-0.64109	0	1.28266	0.000133
C	4	1.01973	0	0.075672	0.000005
C	5	-0.20913	2.343383	-0.01621	-0.000003
C	6	-0.20913	-2.343383	-0.01621	0.000001
H	7	0.18598	2.437057	0.607365	-0.890055
H	8	0.18848	3.099045	-0.798331	-0.000033
H	9	0.18598	2.437016	0.607258	0.890128
H	10	0.18848	-3.099046	-0.79833	-0.000016
H	11	0.18598	-2.437064	0.607356	-0.890057
H	12	0.18598	-2.43701	0.607269	0.890125

Supplementary Table S12. Natural atomic charges (NPA) and Cartesian coordinates of DEC, EMC, and DMC molecules.

Cell 1	Capacity of 5th cycle	Capacity of 50th cycle	Capacity of 100th cycle	Capacity of 200th cycle	Capacity of 300th cycle
**Experimental Value (mAh/g)**	724.70	512.22	495.07	559.78	562.05
**MatGD Mining value (mAh/g)**	722.50	514.56	497.54	563.40	566.36
**Error (mAh/g)**	-2.2	2.34	2.47	3.62	4.31

Cell 2	Capacity of 5th cycle	Capacity of 50th cycle	Capacity of 100th cycle	Capacity of 200th cycle	Capacity of 300th cycle
**Experimental Value (mAh/g)**	178.36	128.87	119.55	130.72	122.33
**MatGD Mining value (mAh/g)**	175.64	131.24	122.36	133.46	124.58
**Error (mAh/g)**	-2.72	2.37	2.81	2.74	2.25

Cell 3	Capacity of 5th cycle	Capacity of 50th cycle	Capacity of 100th cycle	Capacity of 200th cycle	Capacity of 300th cycle
**Experimental Value (mAh/g)**	946.16	779.80	743.54	728.48	697.16
**MatGD Mining value (mAh/g)**	943.76	781.70	743.96	725.46	698.08
**Error (mAh/g)**	2.40	-1.90	-0.42	3.02	-0.92

Supplementary Table S13. Comparison of values at 5, 50, 100, 200, and 300 cycles between experimental data and MatGD extracted data.

Initial Capacity Prediction	Without DFT data	With DFT data
Model	Random Forest	Random Forest
R^2 Score	0.7530	0.7536
MAE(mAh/g)	10.6831	10.7177
Top 10 SHAP features	Ni, Mn, Co, C rate, Loading, Solvent Estate VSA6, Solvent Kappa3, InOr, Solvent MinAbsIndex, Solvent SMR VSA6	Ni, Co, Mn, C rate, Loading ,Solvent Estate VSA6, Solvent Kappa3, Diffusion Coefficient, Solvnet SlogP VSA2, Solvent MinAbsIndex

50th Capacity Prediction	Without DFT data	With DFT data
Model	Random Forest	Random Forest
R^2 Score	0.6866	0.6891
MAE(mAh/g)	12.6029	12.4843
Top 10 SHAP features	Ni, C rate, Loading, Co, Li Clusters, Mn, InOr, Solvent BertzCT, Solvent 1.9, Solvent Chi0v	Ni, C rate, Loading, Co, Li Clusters, Mn, InOr, Solvent BertzCT, ratio of active material

Supplementary Table S14. Performance comparison of predicting models for initial capacity and 50th cycle capacity with and without inclusion of DFT derived descriptors.

		ACS Central Science	Science Advances	Joule
Number of cells		0	**1**	0
Cathode	Name cathode	0	0	0
	Active material	0	0	0
	ratio_of active_material	0	0	0
	Conductive carbon	0	0	0
	Binder	0	0	0
	ratio of cathode weight	0	0	0
	area loading of active_material	0	0	0
Electrolyte	Name electrolyte	0	0	0
	Li_salt material	0	0	0
	Li_salt concentration	0	0	0
	Li_salt concentration unit	0	0	0
	Solvent material	0	0	0
	Solvent volume ratio	0	0	0
	Solvent volume ratio_unit	0	0	0
	EA ratio	0	0	0
Anode	Name anode	0	0	0
	thickness	0	0	0
	unit	0	0	0
	additional layer	0	0	0
Separator	Separator material	0	0	0
Current collector	collector1 (cathode)	0	0	0
	collector2 (anode)	0	0	0

Measurement condition	C-rate	**1**	0	0
	Current density	0	0	0
	Current density unit	0	0	0
	Temperature	0	0	0
	Temperature unit	0	0	0

Supplementary Table S15. External validation of the text mining error across different publishers. We manually verified accuracy of the proposed mining workflow applied to selected articles ACS Central Science[1], Science Advances[2], and Joule[3]. The table summarizes the number of error counts for each article. One cell was not identified in the Science Advances article due to insufficient reference cell information in both the result and caption texts. In the ACS Central Science article, one C-rate value was not extracted because the information was provided only within the cycle graph and was not accessible through text mining.

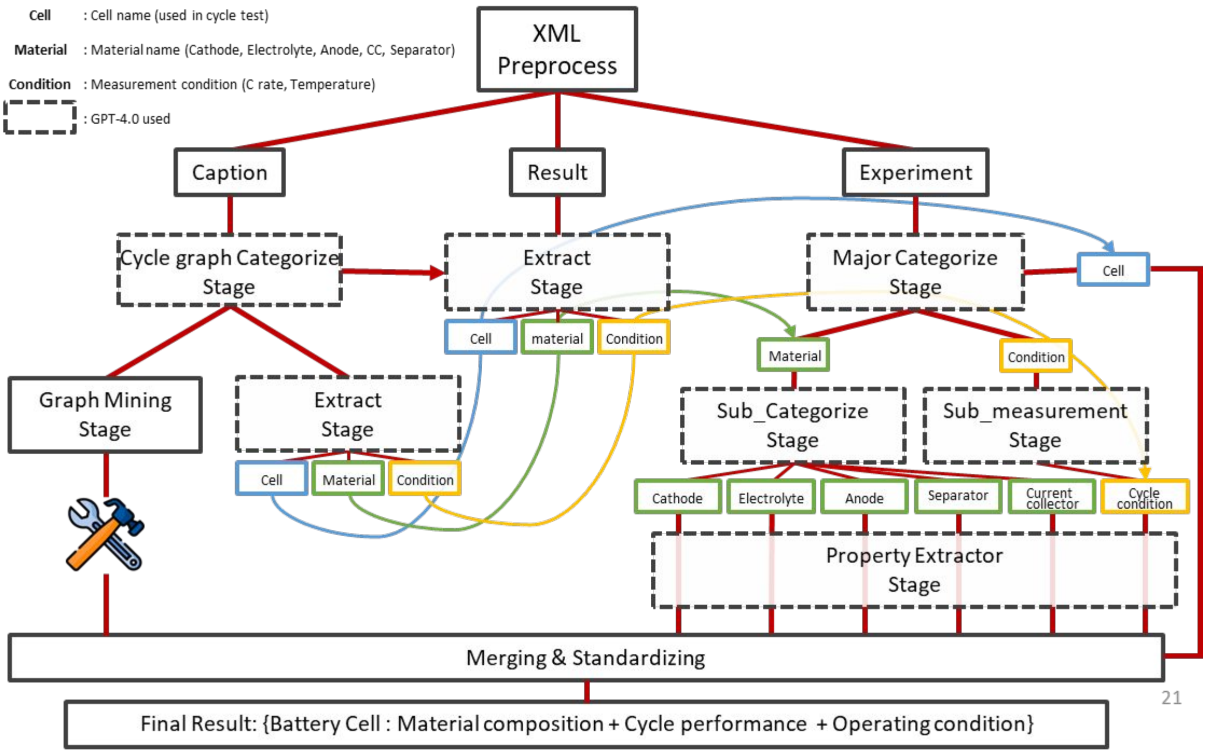

Supplementary Figure S1. Schematic illustration of ABC (Automatic Battery data Collector) working process. Dashed box is where GPT-4.0 is used. Red arrows show how the data is interchanged.

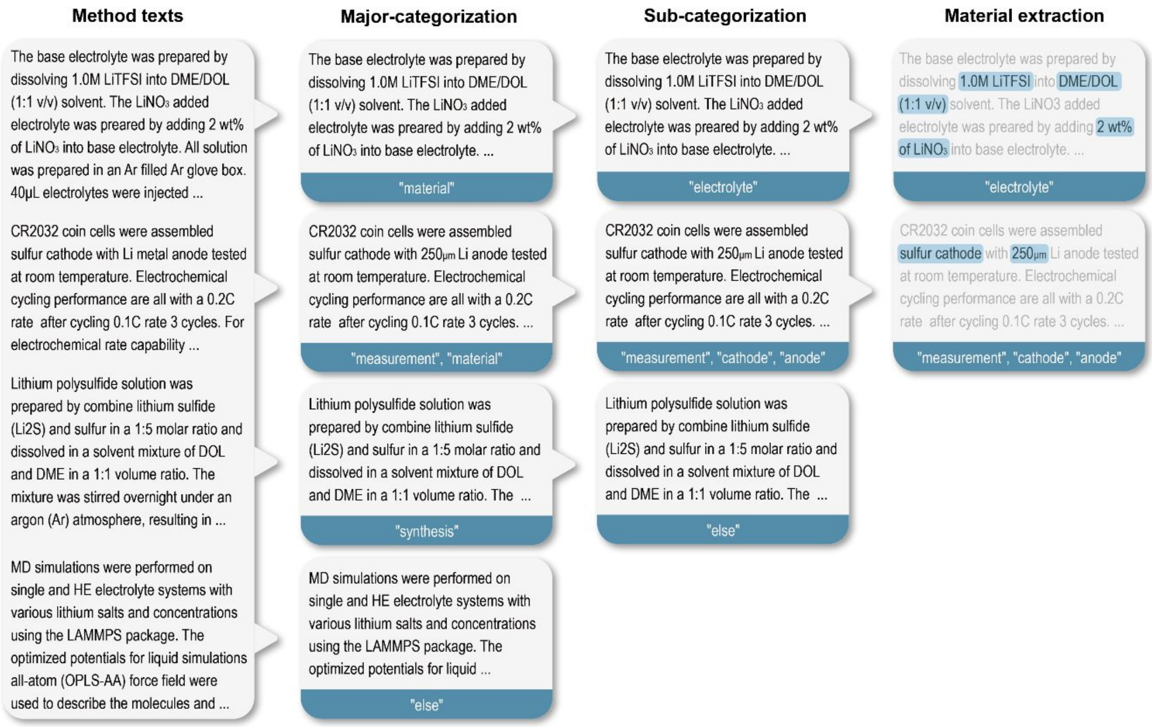

Supplementary Figure S2. An illustration of material extraction results using LLM with detailed Major- and Sub-categorization of method texts. Each categorization result is recorded in the blue area. Major-categorization consisted of “material”, “measurement”, “synthesis”, and “else”. The Sub-categorization categorized according to Major-categorization (“material”: cathode/ electrolyte/anode/separate/current collector/else, “measurement”: measurement/else). “Synthesis” has significance when it is paired with “material” but is Sub-categorized as “else” when it is without “material”.

PROMPT_FIGURE

Role

Extract the graph label from given caption which graph corresponds to capacity vs. cycle number.

Format

```
There are guildlines for output format:
- You must write like
[
    {{
    "graph_data" : {{
        "figure" : int,
        "graph_label" : str
        }}
    }}, ...
]
```

Information

You must follow rules below.
- Capacity vs. cycle number graphs are often accompanied by Coulombic efficiency. If only Coulombic efficiency exist without capacity, please don't extract it.
- Capacity vs. cycle number graph is a cycle test graph that measures the charge or discharge capacity of a battery cell in each cycle. This test is also called by various terms, such as electrochemical cycling, cycling performance, longterm cycling performance, cycle-capacity performance, cycle stability test, galvanostatically cycle, galvanostatic charge discharge test, etc.
- Do not include another electrochemical measurement(Coulombic efficiency graph, rate capability test, Electrochemical Impedance Spectroscopy, Cycle Voltametry test, Li stripping/plating measurement, galvanostatic Li plating/stripping test, charge-discharge profiles, profile, ...) that are not cycle test.
- You must return empty list when the graph is not cycle test.
- For unknown information, use the value null.

Begin!

Example

caption : fig. 1. (a,b) cycling performances of LFP Li metal battery was measured at 0.1C, and 1.0C at 40°C. (c) Rate capability of the AF–LMB with PI@Au nanofibers examined during the rate increase from 0.1 to 1 C. (d) GCD profile of the AF-LMB with PI@Au nanofibers at 1st, 50th, 100th and 150th cycles

output :

```
[
    {{
    "graph_data" : {{
        "figure" : 1,
        "graph_label" : "a"
        }}
    }},
    {{
    "graph_data" : {{
        "figure" : 1,
        "graph_label" : "b"
        }}
    }}
]
```

caption: {caption}
output:

Supplementary Figure S3. The prompt example for the cycle graph extraction model from captions. Each component is highlighted with a different colored box corresponding to its Role, Format, Information, and Example.

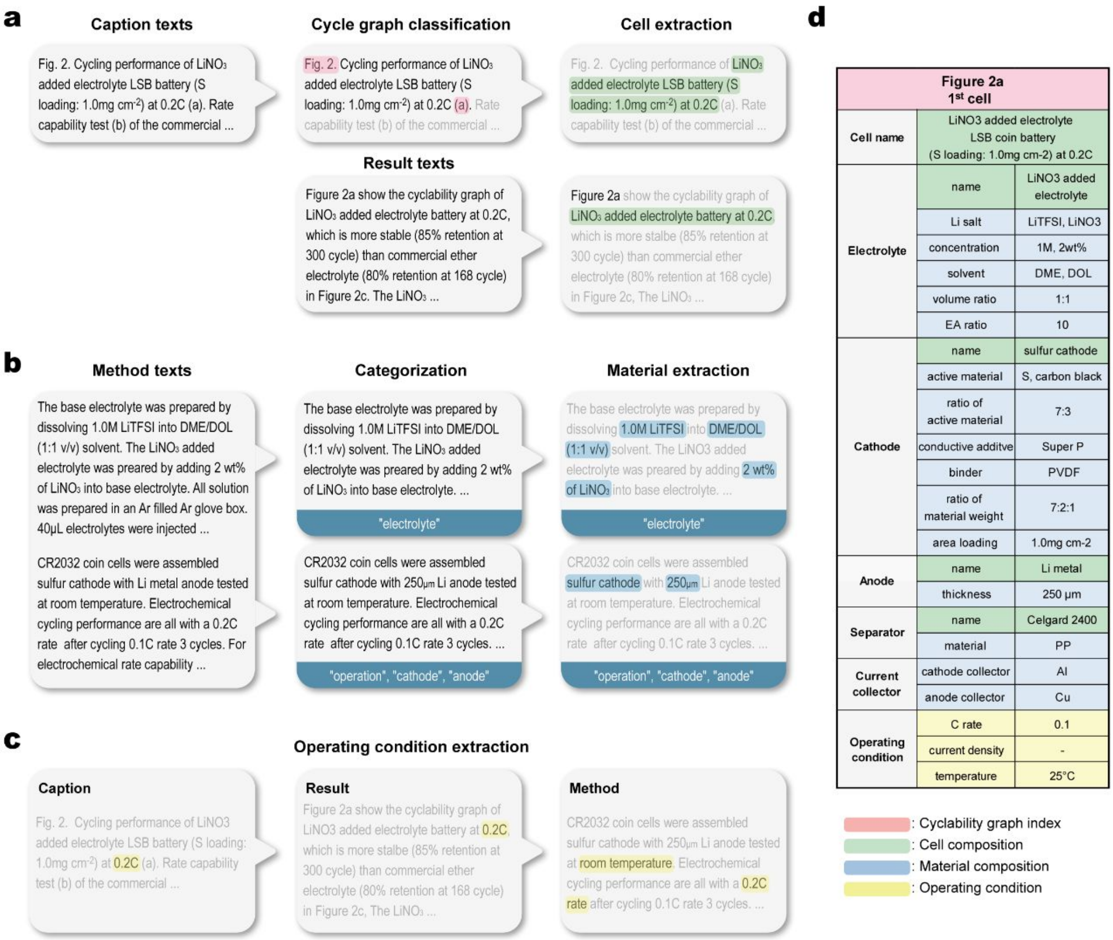

Supplementary Figure S4. Example of the text mining procedure. Overall process of each text extraction process is illustrated: **a** cycle graph metadata and cell composition, **b** material composition, and **c** operating condition. The extraction results are highlighted and the categorization results are written within the gray boxes. **d** An example of the text mining result, with relevant content colorized for each extraction.

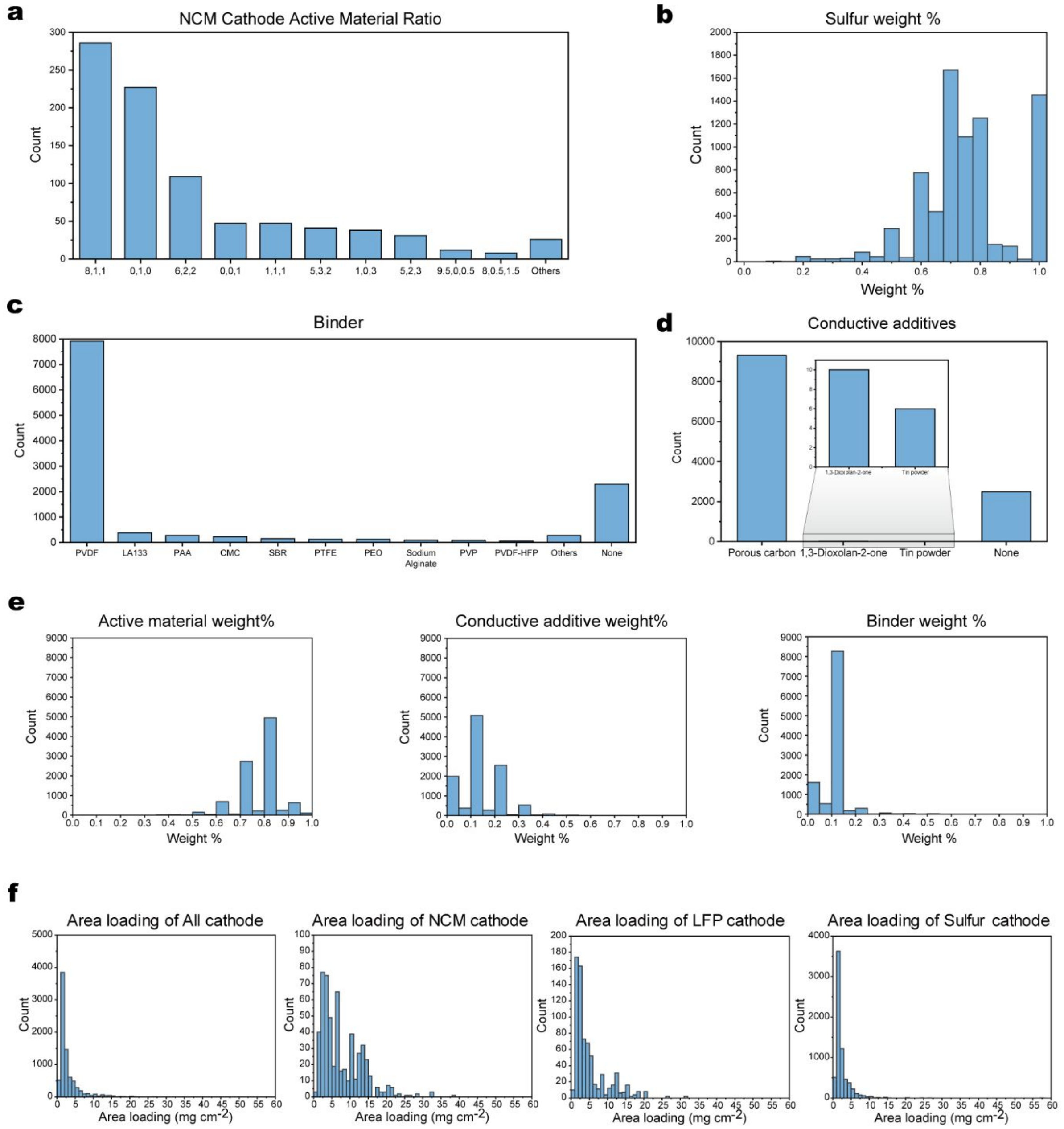

Supplementary Figure S5. Histogram graphs for **a** active material ratio of NCM cathodes, **b** weight percentage of LSB cathodes, **c** binder types, **d** types of conductive additives, **e** weight percent of active material, conductive additive, and binder, and **f** area loading of different cathode type LMBs.

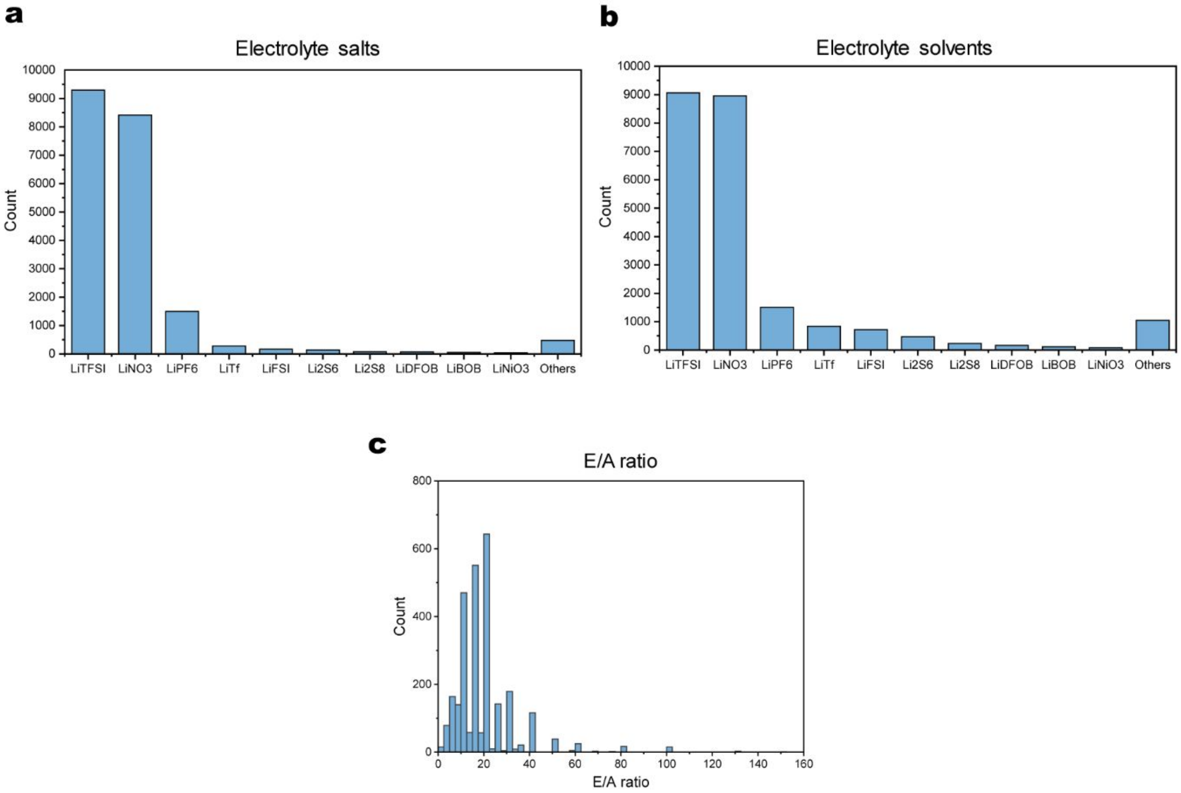

Supplementary Figure S6. Histogram graphs for Electrolyte **a** salts, **b** solvents and **c** E/A (electrolyte/active material) ratio.

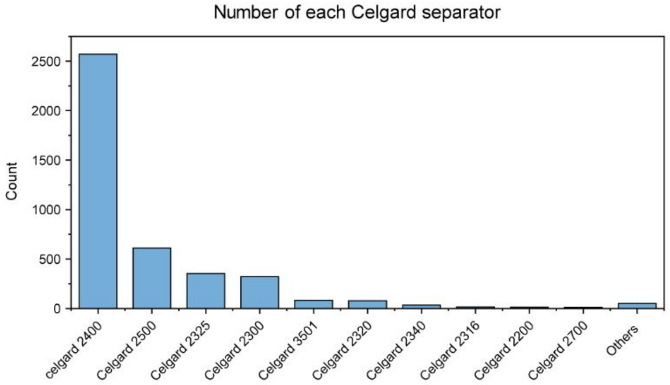

Supplementary Figure S7. Histogram graphs for types of Celgard separators.

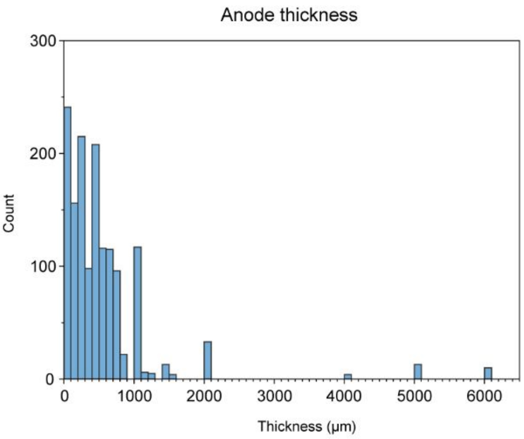

Supplementary Figure S8. Histogram graphs for thickness of anodes.

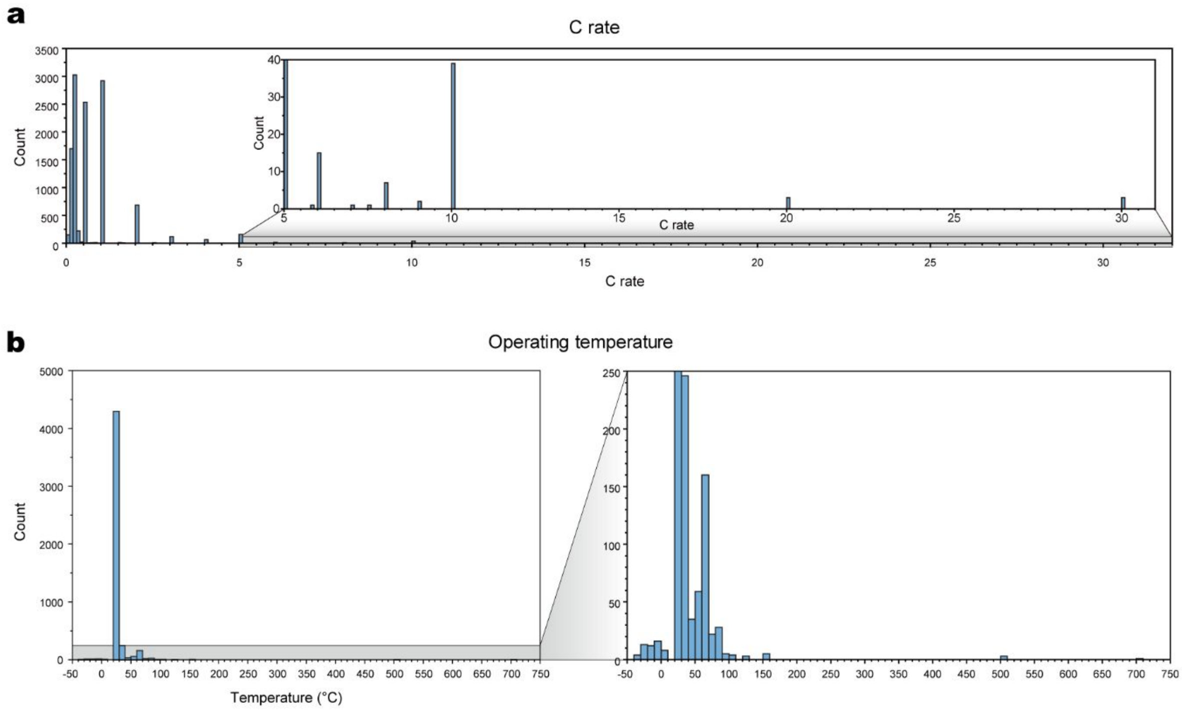

Supplementary Figure S9. Histogram graphs for operating conditions of **a** C rate, and **b** temperature.

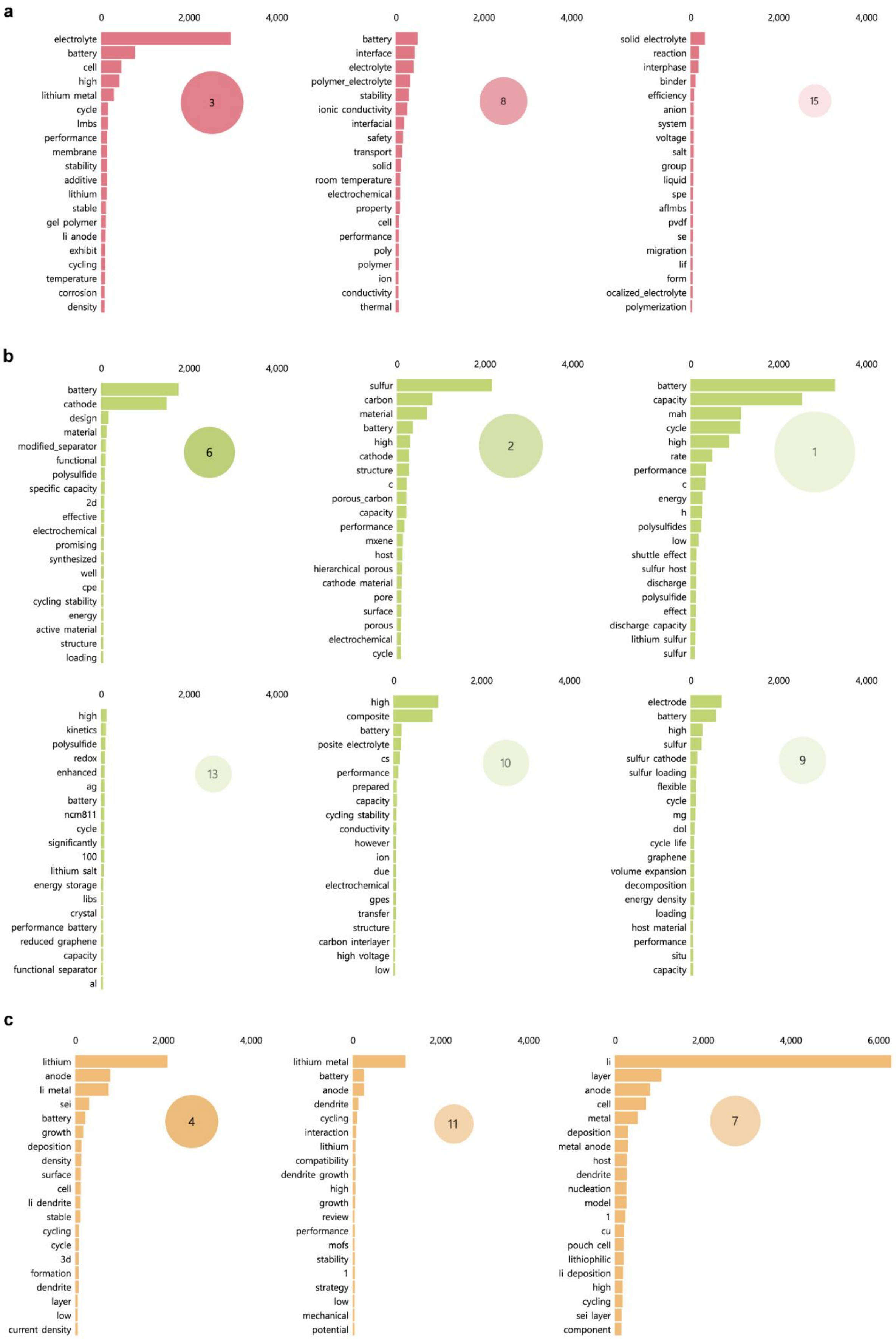

a
0
2,000
4,000
electrolyte
battery
cell
high
lithium metal
cycle
lmbs
performance
membrane
stability
additive
lithium
stable
gel polymer
li anode
exhibit
cycling
temperature
corrosion
density
3
battery
interface
electrolyte
polymer_electrolyte
stability
ionic conductivity
interfacial
safety
transport
solid
room temperature
electrochemical
property
cell
performance
poly
polymer
ion
conductivity
thermal
8
solid electrolyte
reaction
interphase
binder
efficiency
anion
system
voltage
salt
group
liquid
spe
aflmbs
pvdf
se
migration
lif
form
ocalized_electrolyte
polymerization
15
b
battery
cathode
design
material
modified_separator
functional
polysulfide
specific capacity
2d
effective
electrochemical
promising
synthesized
well
cpe
cycling stability
energy
active material
structure
loading
6
sulfur
carbon
material
battery
high
cathode
structure
c
porous_carbon
capacity
performance
mxene
host
hierarchical porous
cathode material
pore
surface
porous
electrochemical
cycle
2
battery
capacity
mah
cycle
high
rate
performance
c
energy
h
polysulfides
low
shuttle effect
sulfur host
discharge
polysulfide
effect
discharge capacity
lithium sulfur
sulfur
1
high
kinetics
polysulfide
redox
enhanced
ag
battery
ncm811
cycle
significantly
100
lithium salt
energy storage
libs
crystal
performance battery
reduced graphene
capacity
functional separator
al
13
high
composite
battery
posite electrolyte
cs
performance
prepared
capacity
cycling stability
conductivity
however
ion
due
electrochemical
gpes
transfer
structure
carbon interlayer
high voltage
low
10
electrode
battery
high
sulfur
sulfur cathode
sulfur loading
flexible
cycle
mg
dol
cycle life
graphene
volume expansion
decomposition
energy density
loading
host material
performance
situ
capacity
9
c
lithium
anode
li metal
sei
battery
growth
deposition
density
surface
cell
li dendrite
stable
cycling
cycle
3d
formation
dendrite
layer
low
current density
4
lithium metal
battery
anode
dendrite
cycling
interaction
lithium
compatibility
dendrite growth
high
growth
review
performance
mofs
stability
1
strategy
low
mechanical
potential
11
6,000
li
layer
anode
cell
metal
deposition
metal anode
host
dendrite
nucleation
model
1
cu
pouch cell
lithiophilic
li deposition
high
cycling
sei layer
component
7

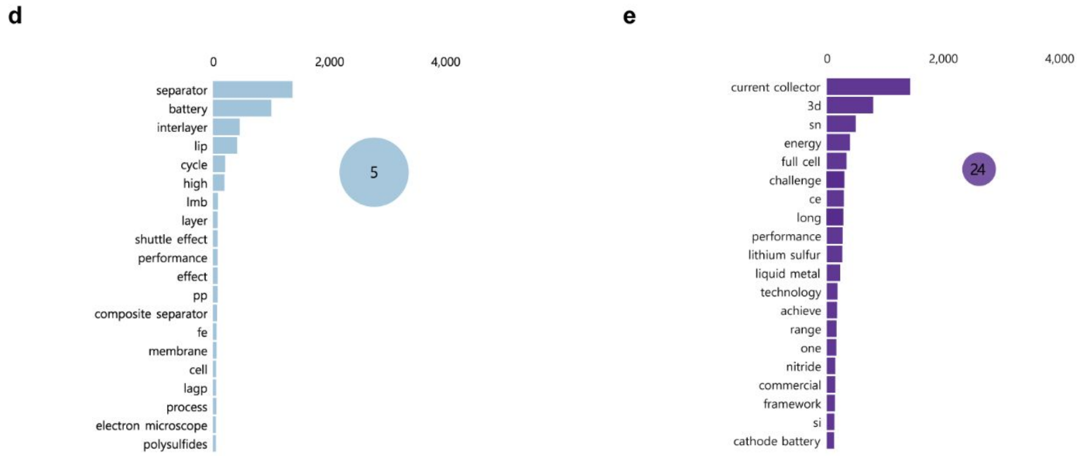

Supplementary Figure S10. Top-20 most relevant terms for topic **a** electrolyte, **b** cathode, **c** anode, **d** separator, and **e** current collector. From the LDA (Latent Dirichlet Allocation) results, the most frequently appearing keywords associated with each topic provide insight into the corresponding research trends.

In the topic of the electrolyte, keywords such as ‘lithium metal’, ‘high performance’, ‘stability’ and ‘ionic conductivity’ were frequently observed, In the topic of the cathode, keywords such as ‘sulfur’, ‘carbon’, ‘high capacity’, and ‘structure’ appeared frequently, reflecting interest in material design and capacity enhancement. In the topic of anode, keywords included ‘SEI’, ‘dendrite’, and ‘deposition’. The separator topics involved ‘interlayer’ and ‘lithium polysulfide’, while the current collector topics included ‘3D structure’ and ‘Sn’.

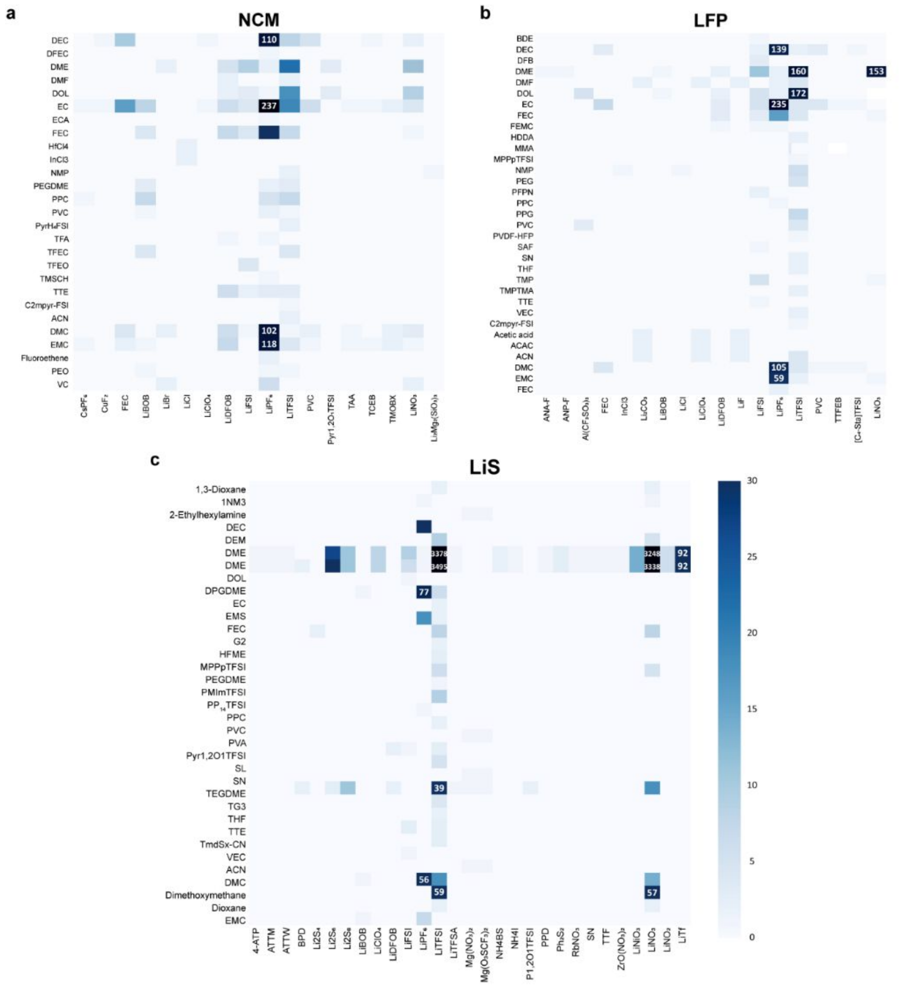

Supplementary Figure S11. The heatmaps illustrate the relationship between salt (x axis) and solvent (y axis) pairs in the electrolytes of **a** NCM, **b** LFP, and **c** LiS cathode cells. To clearly visualize relationships with low frequency pairs, occurrences of under 30 are represented using a color bar, while frequencies above 30 are indicated with numerical labels.

For NCM cathodes, carbonate-based solvents such as EC, DEC, and DMC were predominantly used due to their high oxidation stability, allowing operation at voltages up to ~4.2V. Additionally, $LiPF_6$ salt is well-suited for use with carbonate-based solvents, facilitating the formation of a stable SEI/CEI even in high-voltage conditions. In contrast, LFP operates at a relatively low voltage of ~3.4V, making both carbonate-based and ether-based solvents (e.g., DME and DOL) viable options. For LSB systems, ether-based solvents were used almost exclusively due to their compatibility with lithium polysulfide species and their ability to stabilize intermediate phases. This preference arises from the superior solubility of lithium salts in ether solvents and their effectiveness in suppressing polysulfide shuttling, which enhances the overall stability of LSB cells. Solvent selection was also closely linked to salt compatibility. Carbonate solvents were typically paired with $LiPF_6$, while ether solvents were commonly combined with LiTFSI and $LiNO_3$. $LiPF_6$ dissolves well in carbonate solvents but readily decomposes in ether solvents, generating HF and other byproducts. In contrast, LiTFSI exhibits excellent thermal and chemical stability in ether solvents but can corrode the aluminum current collector in carbonate-based electrolytes.

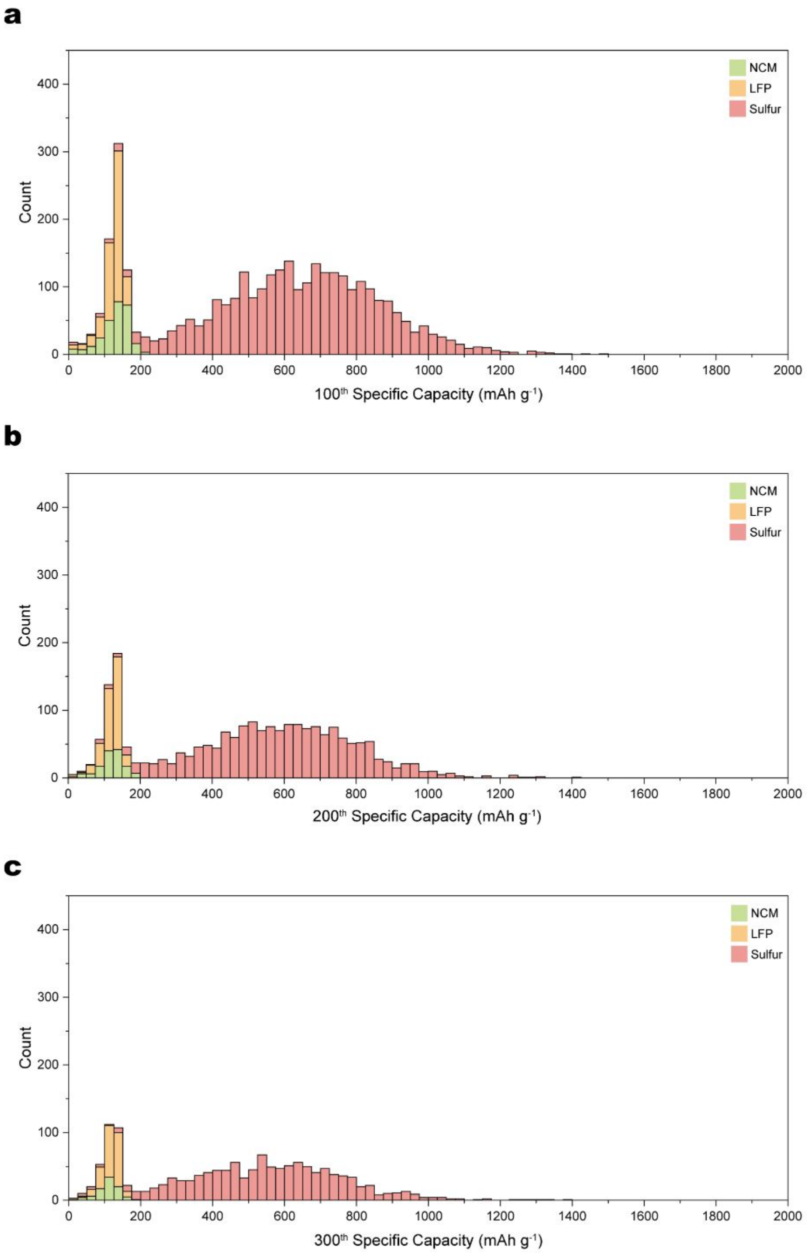

Supplementary Figure S12. Data distribution for the **a** 100th, **b** 200th, **c** 300th cycle capacity for different cathode types.

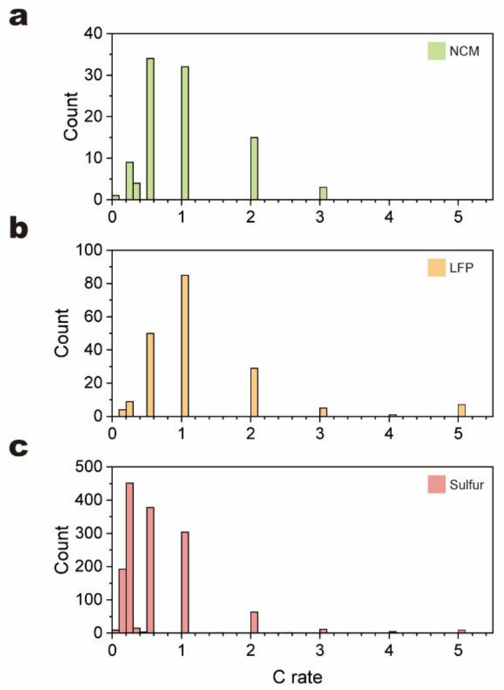

Supplementary Figure S13. C-rate data distributions for **a** NCM, **b** LFP, and **c** Sulfur cathode LMBs in our database.

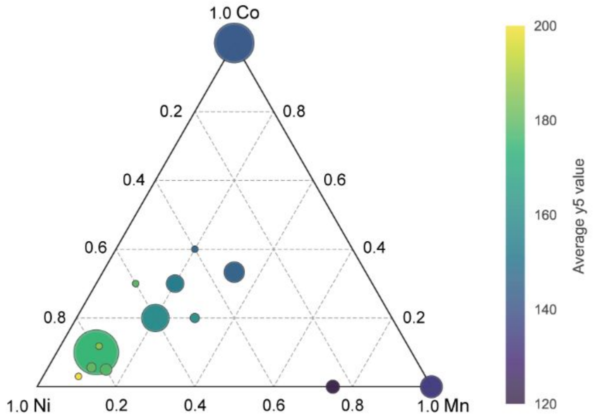

Supplementary Figure S14. A ternary plot of the distribution of Ni, Co, and Mn stoichiometry ratio. Higher initial capacities are indicated by colors shifting from purple to yellow, and the circle size means the frequency of the composition.

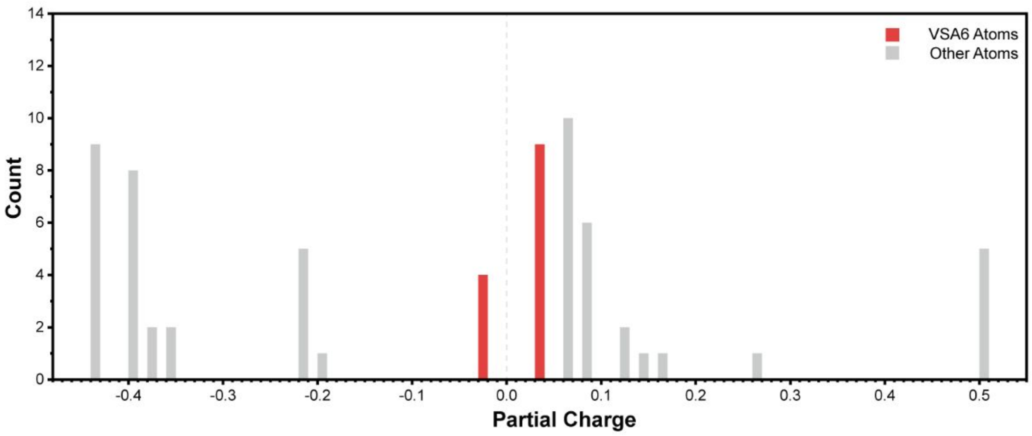

Supplementary Figure S15. Partial charge distribution in solvent molecules. Atoms within the EState VSA6 range for each solvent molecules are highlighted in red. All atoms with near-zero partial charges are found within the VSA6 range.

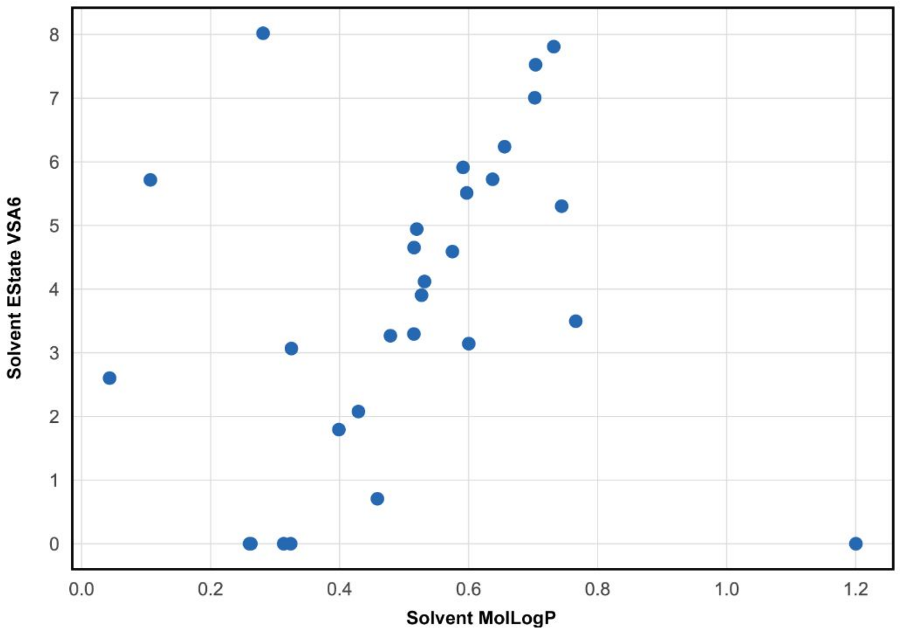

Supplementary Figure S16. Correlation between MolLogP and Estate VSA6 solvent molecular descriptors. MolLogP represents the octanol-water partition coefficient of a molecule, which reflects its polarity.

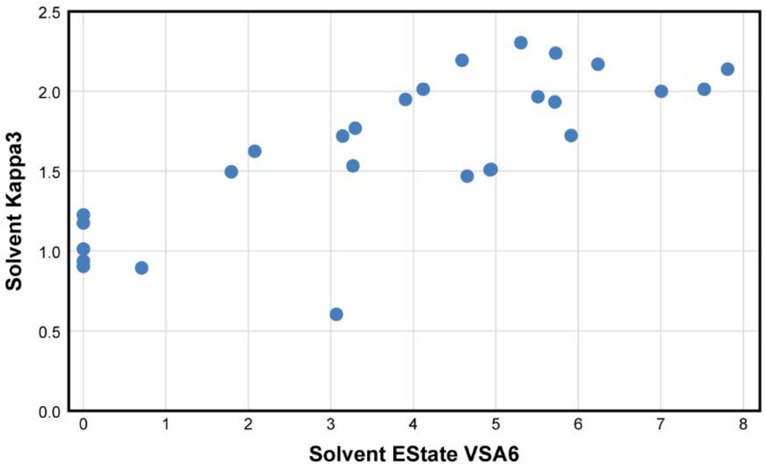

Supplementary Figure S17. Scatter plot illustrating the relationship between Estate VSA6 and Kappa3 values of solvent molecules which are used in NCM cathode cells.

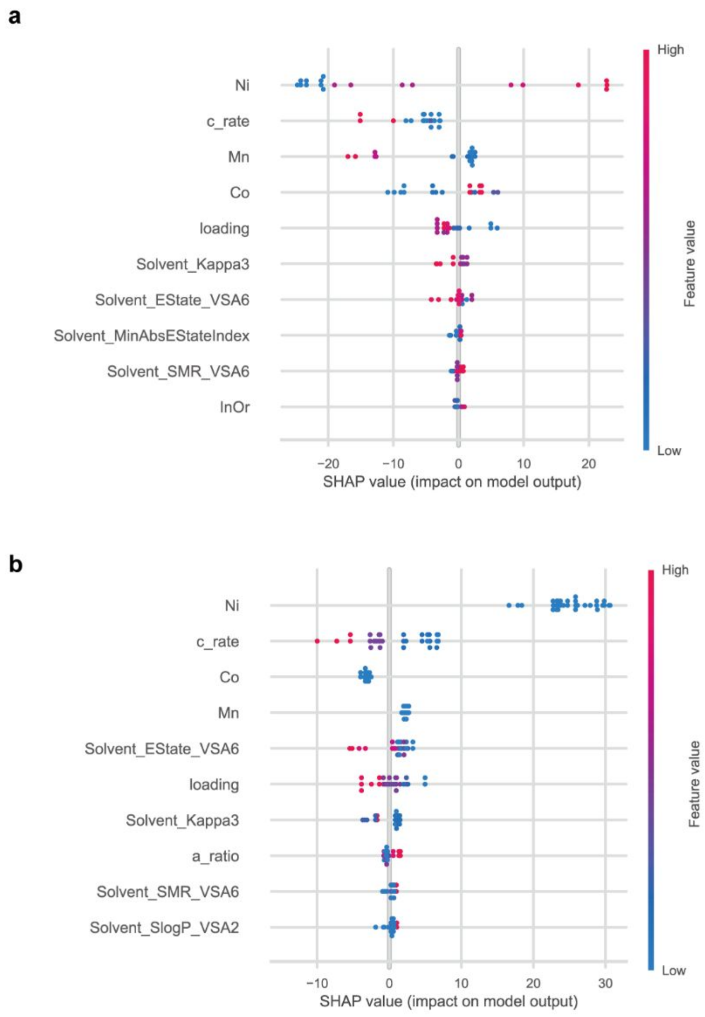

Supplementary Figure S18. SHAP feature analysis of initial capacity prediction for NCM cathode with a high C rate (greater than 1 C), and high Ni content (80% of Ni) conditions.

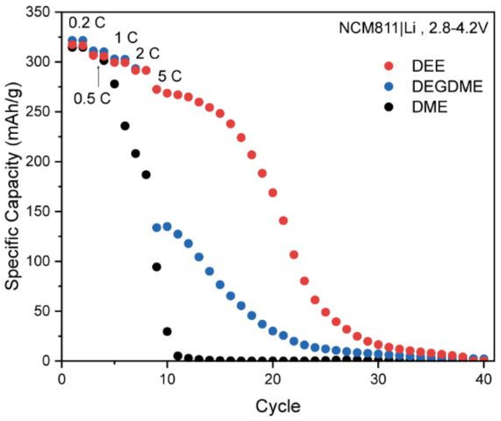

Supplementary Figure S19. Cycling performance of Li||NCM811 coin cells at 5C-rate with 1M LiFSI in DEE, DEGDME and DME. A rapid capacity decline was observed for DME because of its reaction with the electrode. Two precycles at 0.2 and 0.5C were performed.

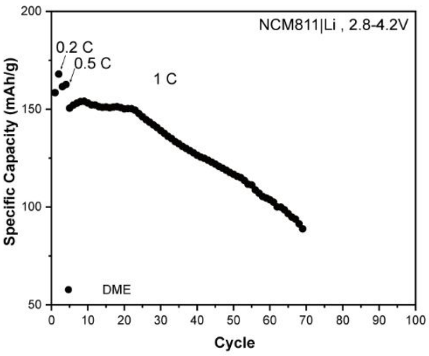

Supplementary Figure S20. Cycling performance of Li||NCM811 coin cells at 1C-rate with 2M LiFSI in DME. Two precycles at 0.2 and 0.5C were performed.

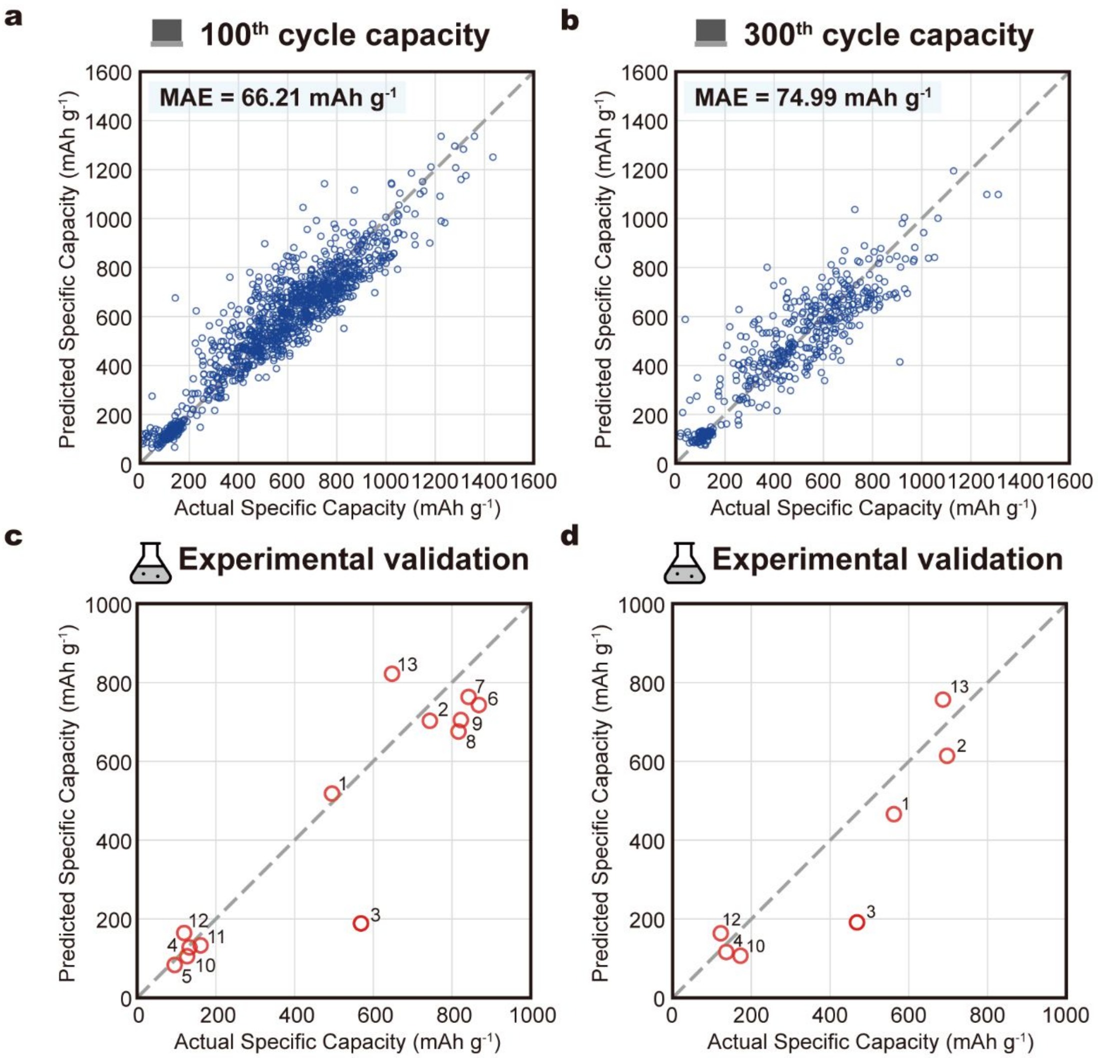

Supplementary Figure S21. Capacity prediction and experimental validation of the 100th and 300th cycles in LSBs. Gradient boosting regression (GBR) models were employed to predict capacity.

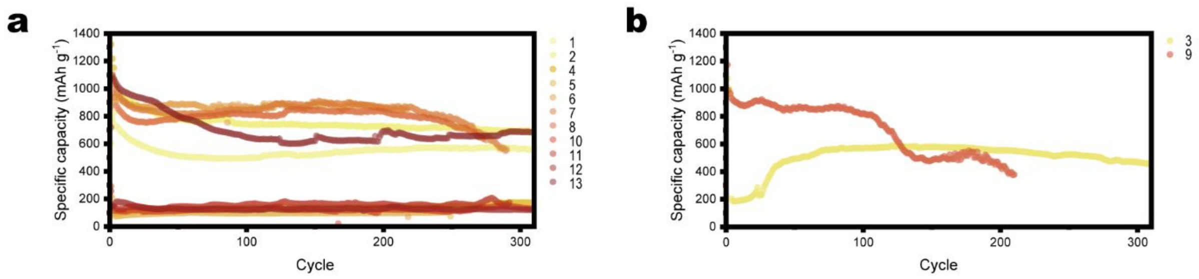

Supplementary Figure S22. a Cycle graph of lithium sulfur batteries in various experimental conditions. **b** Cycle graph of processively unstable assembled cells. The indexes 1-13 correspond to **Supplementary Table 8**.

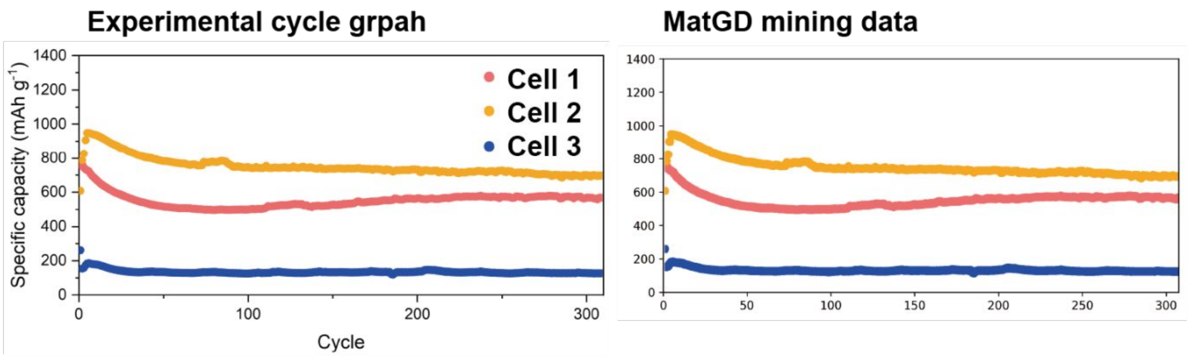

Supplementary Figure S23. Comparison between experimental data and MatGD extracted data.

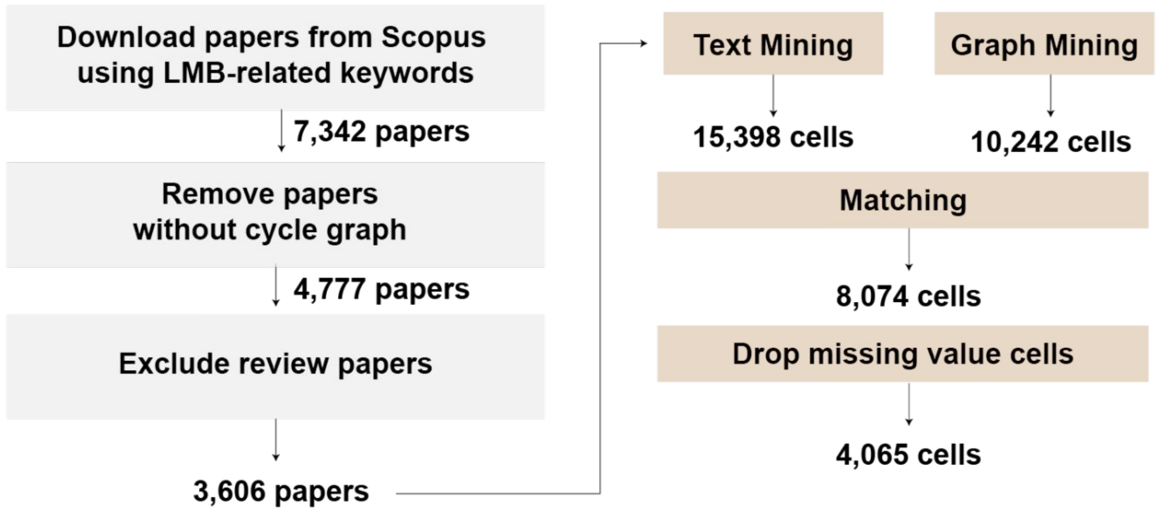

Supplementary Figure S24. Data curation funnel for constructing the LLMB database

A total of 7,342 papers were initially retrieved from Scopus using LMB-related keywords. Papers without cycling graphs were removed, yielding 4,777 papers. After excluding review articles, 3,606 research papers remained. From the 3,606 selected papers, text mining extracted 15,398 cells, while graph mining extracted 10,242 cells These two sets of extracted cells were then matched, resulting in 8,074 cells with paired information. For machine learning analysis, cells containing missing values were excluded, leading to a final dataset of 4,065 cells used for model training and evaluation.

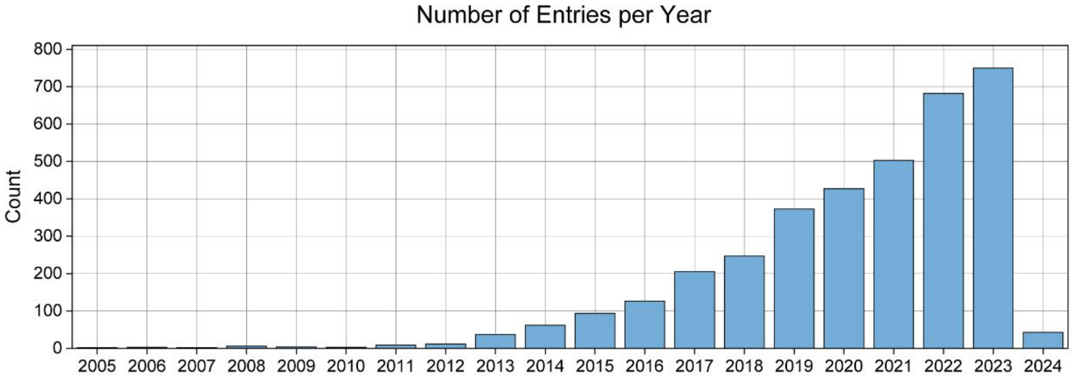

Supplementary Figure S25. Data distribution of published research papers about lithium metal batteries over the last 20 years. Entries for 2024 included until January 2024.

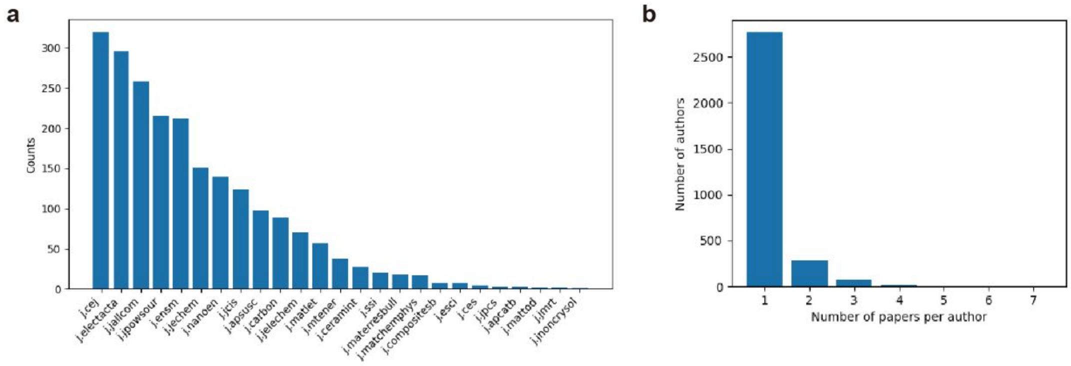

Supplementary Figure S26. Data coverage across the journals and chemistries in machine learning dataset. a Distribution of publications included in the machine learning dataset across journals (top 25 shown). The dataset is broadly distributed among electrochemistry and energy-related journals, with no single journal dominating the entries. All journals not shown in the figure contribute fewer than 10 records each. b Author frequency distribution of publication groups defined by combined first and corresponding authors. The x-axis represents the number of repeated occurrences per group, and the y-axis represents the number of groups with that frequency, indicating no strong concentration within a limited number of research groups.

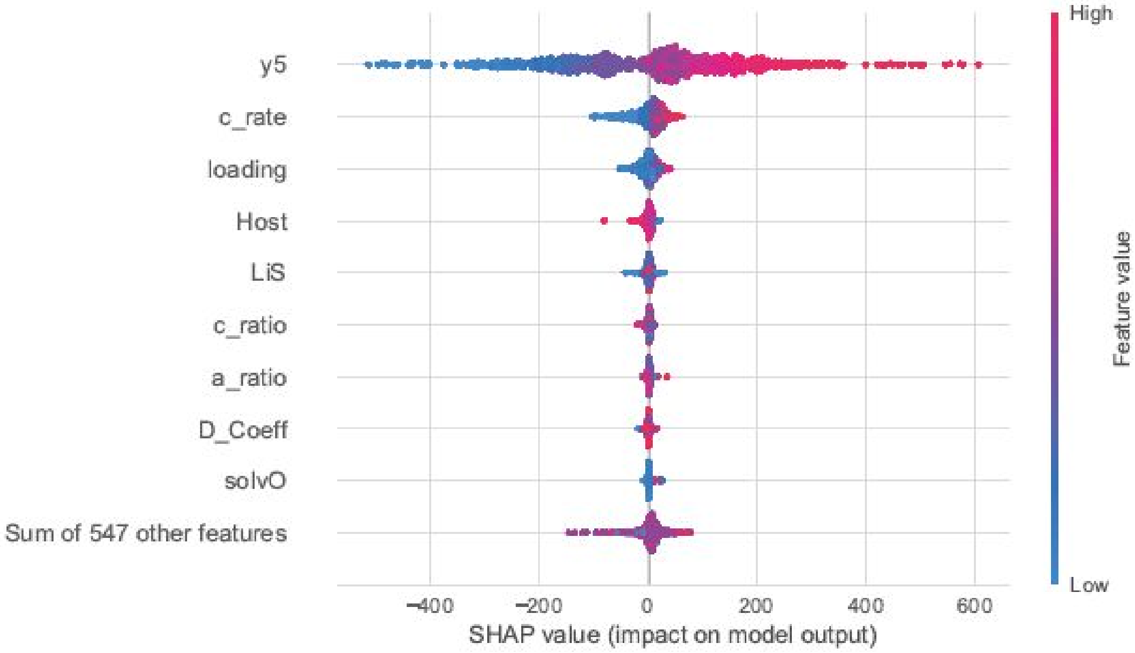

Supplementary Figure S27. SHAP plot of 200th cycle capacity prediction model for lithium sulfur battery

References

1. Ma P, Mirmira P, Amanchukwu CV. Effect of building block connectivity and ion solvation on electrochemical stability and ionic conductivity in novel fluoroether electrolytes. *ACS Central Science* **7**, 1232-1244 (2021).

2. Xiong Q, *et al*. A practical 4.8-V Li||$LiCoO_2$ battery. *Science Advances* **11**, eadx5020 (2025).

3. Chang Z, *et al*. A liquid electrolyte with de-solvated lithium ions for lithium-metal battery. *Joule* **4**, 1776-1789 (2020).
