# Supplementary material for: LLMB: AI Agent for Lithium Metal Battery Research Using Large Language Model
Source: ACS Cent Sci. 2026 Mar 17;12(4):484–96. doi: 10.1021/acscentsci.5c02433 (PMC13107216; doi:10.1021/acscentsci.5c02433)
Supplement: Supplementary file 2 [file oc5c02433_si_002.pdf]

oc-2025-024339.R1

Name: Peer Review Information for "LLMB: AI agent for lithium metal battery research using large language model"

First Round of Reviewer Comments

Reviewer: 1

Comments to the Author

The manuscript presents an ambitious and potentially impactful LLM-based agent (LLMB) that integrates text mining and cycling-curve graph mining to construct a battery database and demonstrate ML-driven insights and validation. The manuscript is generally clear and well-organized. With targeted revisions to improve methodological clarity, bias control, reproducibility, and figure readability, the work should be suitable for publication in ACS Central Science. The following issues should be addressed before publication.

1. The manuscript describes filtering from 7,342 to 3,606 papers but does not clearly specify a robust protocol for dataset cleaning and bias control (e.g., duplicate cells from the same DOI, missing-field patterns, outlier handling, and coverage across years/journals/chemistries). Please provide a concise “data curation funnel” and key dataset statistics to support the credibility of the downstream ML results.

2. Fig. 2b labels the first step as “Relevant Text Parsing,” while the described operation appears to be paragraph extraction/retrieval/selection using Python scripts, followed by LLM-based classification/extraction. Because “parsing” commonly implies semantic or syntactic analysis, this terminology may overstate what the script does. Please revise the label and briefly describe the extraction heuristic to enhance reproducibility.

3. The manuscript limits the literature corpus to Elsevier sources but suggests this ensures diversity in publication formats. This is not well supported and may introduce publisher-specific style/format biases. Please rephrase the statement more conservatively and discuss potential publisher-format bias and its implications for generalization (or provide a small external validation to demonstrate transferability).

4. The workflow assumes coulombic efficiency (CE) “stabilizes around 100% after a few cycles,” and uses this assumption to remove CE curves during graph mining. This may not hold for many lithium-metal battery reports where CE can remain below 100% or fluctuate. Please provide a quantitative check or an ablation study showing how CE removal affects mined curve yield and key dataset statistics.

5. Figs. 4 (overall), 4b, and 5b: Several labels are too light and/or too small, and font type/size are not fully consistent within Fig. 4. Please standardize font family, font size hierarchy, and contrast across these panels. Fig. 5c: Please add a clear legend indicating which element corresponds to each sphere color in the ball-and-stick model. Fig. 5d: Please include a colorbar and specify the isosurface value(s) and explicitly define whether red/blue represent positive/negative charge (or electron accumulation/depletion), to avoid ambiguity.

After these issues are satisfactorily addressed, I believe the manuscript will be substantially strengthened and can be recommended for publication in ACS Central Science.

Reviewer: 2

Comments to the Author

This manuscript presents a significant advancement in applying AI agents to the study of lithium-metal batteries. By leveraging a large language model to extract data from both textual and graphical sources, the work not only predicts initial battery capacity but also deepens the understanding of the relationships between electrochemical performance and battery components. The inclusion of experimental validation further strengthens the credibility of the proposed approach. Several key aspects could be clarified or expanded to improve the rigor, interpretability, and generalizability of the findings. Below are specific comments and suggestions intended to strengthen the manuscript.

1. In the graph mining stage, only cyclability data were extracted after removing Coulombic efficiency (CE) curves. Since CE is a critical indicator of the reversibility of Li plating/stripping and a strong predictor of cycle life in lithium-metal batteries, its exclusion may limit the feature set and model performance. Please justify the omission of CE or consider incorporating CE-related metrics (e.g., average CE or cycle number of CE declining rapidly) into the feature extraction process.

2. The accuracy of graph mining has not been evaluated (Table 1). Without validation, potential extraction errors could propagate into the model and affect conclusions. The authors should extract cyclability data (such as capacity retention) from the discussion part or tables in the papers and compare the data from graph mining to validate data credibility.

3. In Figures 4 and 5, many feature abbreviations remain unexplained beyond EState VSA6 and Kappa3. Additionally, the rationale for selecting these specific molecular or electrochemical descriptors is not discussed. Please provide a supplementary table for all feature abbreviations used in the SHAP plots. A brief discussion on why these features were chosen, linking their core physicochemical significance to battery performance, would greatly enhance the interpretability and domain relevance of the analysis.

4. The manuscript employs several machine learning models (RF, XGBoost, GBR, etc.), but the rationale for their selection and the impact of this choice on the results is not clarified.

5. Figure 7 provides a prediction and validation of the 200th-cycle capacity for Li–S batteries, but SHAP analysis is absent for this system. We also strongly suggest that the authors consider incorporating the chemical composition and thickness of the Li metal anode into the SHAP analysis, which could reveal important mechanistic differences among all LMBs. One should note that Li metal anodes are case-by-case divergent in existing literature. [https://doi.org/10.1002/adma.202511817.]

6. The machine learning models rely entirely on data mined from published papers, where very often only the best-performing results and the performance gap between experimental and control groups were exaggerated. This could introduce bias, especially for predicting cycle life. Please discuss this potential limitation.

Reviewer: 3

Comments to the Author

This work reports a multi-modal AI agent integrating LLM-based text mining and MatGD-based graph mining to construct a structured LMB materials-property database from literature, followed by machine learning analysis and experimental validation. This work provides a rich and comprehensive visualization analysis of materials reported in

previously published lithium metal battery studies, offering meaningful insights into the global landscape of lithium metal batteries. In addition, the proposed predictive framework innovatively accounts for the coupled effects of both electrode and electrolyte materials on battery performance.

1. In LMBs, additives play a pivotal role in electrolyte engineering. Does the database constructed in this work incorporate additive-level information, and can it predict how additives impact cell performance?

2. As the dataset is limited to Elsevier publications, whether known state-of-the-art electrolytes or electrodes are captured in the database should be checked (especially those from other publishers) to ensure the representativeness.

3. The descriptors used for solvents in this study do not incorporate quantum-level information (such as HOMO, LUMO), which is crucial for electrochemical stability. Could the inclusion of quantum-chemical descriptors lead to more accurate predictions of electrochemical stability?

4. The N/P ratio is an important operating condition. Is this captured in the database, and does it ensure consistency from this standpoint?

5. The search terms for the collection of papers should be provided.

Author's Response to Peer Review Comments:

Reviewer 1

Overall Comment:

The manuscript presents an ambitious and potentially impactful LLM-based agent (LLMB) that integrates text mining and cycling-curve graph mining to construct a battery database and demonstrate ML-driven insights and validation. The manuscript is generally clear and wellorganized. With targeted revisions to improve methodological clarity, bias control, reproducibility, and figure readability, the work should be suitable for publication in ACS Central Science. The following issues should be addressed before publication.

We appreciate the reviewer’s positive and constructive assessment of our manuscript. In response to the reviewer’s comments, we have carried out targeted revisions to improve methodological clarity, bias control, reproducibility, and figure readability. We have also

revised the manuscript to more explicitly discuss limitations and scope where appropriate. We are grateful for the detailed feedback, which has significantly strengthened the manuscript.

[Comment 1]

The manuscript describes filtering from 7,342 to 3,606 papers but does not clearly specify a robust protocol for dataset cleaning and bias control (e.g., duplicate cells from the same DOI, missing-field patterns, outlier handling, and coverage across years/journals/chemistries). Please provide a concise "data curation funnel" and key dataset statistics to support the credibility of the downstream ML results.

We thank the reviewer for the helpful comment. We agree that a more explicit description of the filtering procedure improves the transparency and reproducibility of our dataset.

In the revised manuscript and the corresponding figure, we now provide a step-by-step data curation funnel. Briefly, 7,342 papers were initially retrieved from Scopus using LMB-related keywords. Papers without cycling graphs were first removed (yielding 4,777 papers), and review articles were subsequently excluded, resulting in a curated set of 3,606 research papers. These papers were then subjected to both text mining and graph mining to extract battery materials and performance data. A total of 15,398 cells were extracted through text mining, while 10,242 cells were extracted through graph mining. The two independently extracted datasets were matched to retain only cells with consistent paired information, resulting in 8,074 cells. Finally, for machine learning analysis, cells with missing values were excluded, leading to a final dataset of 4,065 cells used for model training and evaluation.

[Revised Supplementary Information]

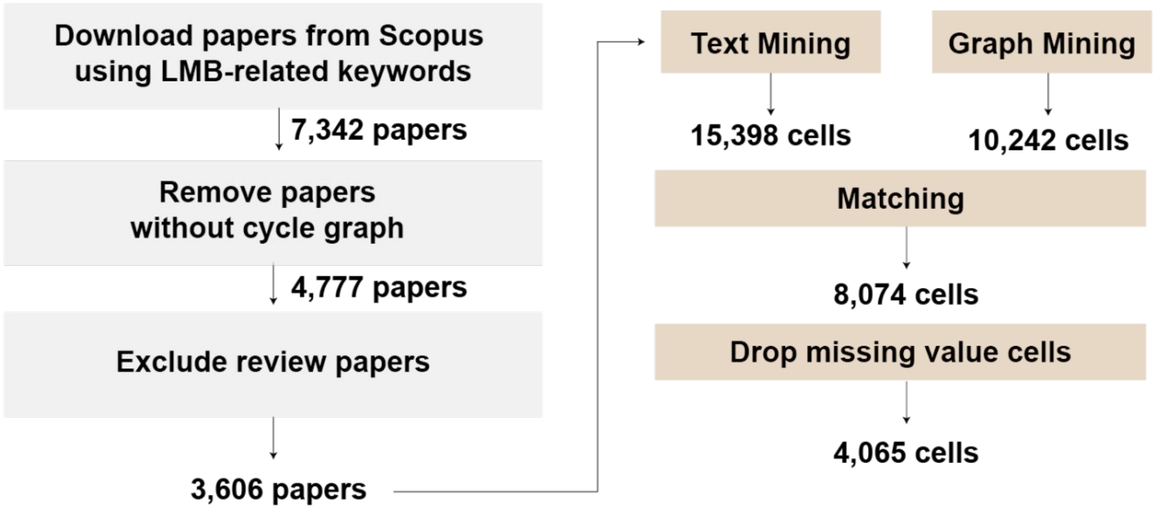

Supplementary Figure 24. Data curation funnel for constructing the LLMB database

A total of 7,342 papers were initially retrieved from Scopus using LMB-related keywords. Papers without cycling graphs were removed, yielding 4,777 papers. After excluding review articles, 3,606 research papers remained. From the 3,606 selected papers, text mining extracted 15,398 cells, while graph mining extracted 10,242 cells. These two sets of extracted cells were then matched, resulting in 8,074 cells with paired information. For machine learning analysis, cells containing missing values were excluded, leading to a final dataset of 4,065 cells used for model training and evaluation.

Regarding the possibility of duplicate cells from the same DOI, our extraction pipeline is designed to prevent such redundancy. Cyclability data are extracted based on explicit cell identifiers referenced in the figure captions and corresponding results paragraphs. Therefore, each dataset entry is anchored to a specific reported cell configuration, which precludes systematic duplication of identical cells within a single article. Rather than duplication, a more realistic risk arises from potential hallucinations. To mitigate this issue, as described in the manuscript, all cells are validated during the matching stage with graph mining data. Only cells for which corresponding cyclability graph are present in the figures are retained.

All extracted information from the text-mined data is saved in a DataFrame where missing entries are explicitly represented as NaN values. For downstream machine learning analysis, we restrict the training dataset to samples in which all required fields are populated, ensuring that models are trained only on complete records. In response to the reviewer's comment, we have added summary statistics of missing field patterns for the 8,074 text mining cells to the supplementary information.

[Revised Supplementary Information]

**Field**	Cathode type	Active material ratio	Cathode weight ratio	Conductive additive	Binder	Loading
**# of missing entries**	2951	2951	1983	1891	2025	3664
**Field**	Li salt	Solvent	Li solvent concentration	Solvent volume ratio	Separator	C-rate
**# of missing entries**	2951	2951	1983	1891	2025	3664

Supplementary Table 11. Summary statistics of missing fields in text mining data

Outlier handling is implicitly enforced through multiple filtering steps during data extraction and preprocessing. Molecular information extracted from text is converted into SMILES representations prior to descriptor generation. Entries for which SMILES conversion fails are automatically removed from the dataset. For numerical quantities such as lithium metal anode parameters and electrolyte composition ratios, the unit conversion procedures described in the manuscript are applied prior to outlier control. The extracted values lie within physically reasonable bounds, with no evident outliers.

Similarly, for cycling data obtained through graph mining, the extracted capacity and cycle number ranges are constrained by established electrochemical limits for lithium metal batteries. This enables manual verification of potential outliers based on known physical

bounds of capacity and cyclability. Although it is not feasible to manually inspect every individual data point, the high extraction accuracy reported in the manuscript, together with error handling during the conversion and extraction steps, supports the conclusion that outliers are effectively controlled.

Coverage across publication years, journals, and chemistries was examined to assess potential bias. As shown in Supplementary Figure 25, the number of LMB-related papers increases gradually over the years, reflecting the natural growth of the field rather than overrepresentation of specific periods. In Supplementary Figure 26, the journal distribution (xaxis: journal name; y-axis: frequency) shows that the dataset is primarily drawn from electrochemistry and energy-related journals. The top 25 journals account for the majority of entries, while the remaining journals each contribute fewer than 10 records. No single journal disproportionately dominates the dataset.

To assess chemistry-related representation, publications were grouped based on combined first and corresponding author pairs. The author frequency distribution (x-axis: number of repeated occurrences; y-axis: number of author groups) indicates that the dataset is not concentrated within a small number of research groups. Overall, no significant bias is observed across publication year, journal source, or authorship distributions.

[Revised Supplementary Information]

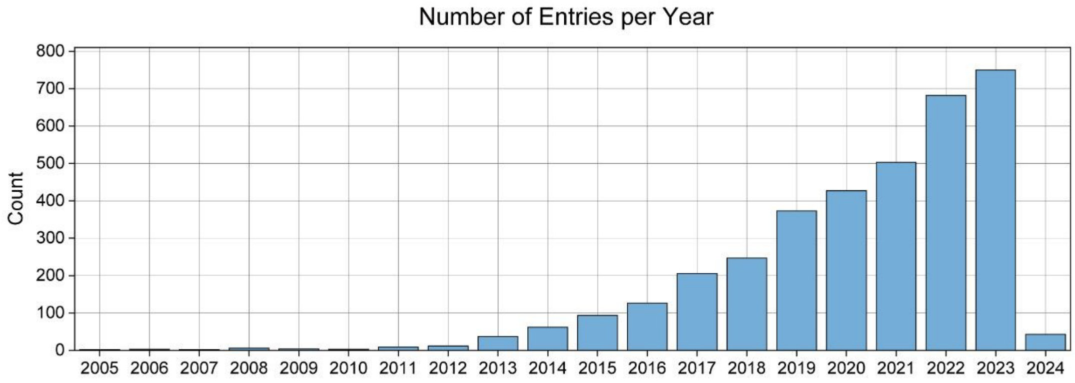

Supplementary Figure 25. Data distribution of published research papers about lithium metal batteries over the last 20 years. Entries for 2024 included until January 2024.

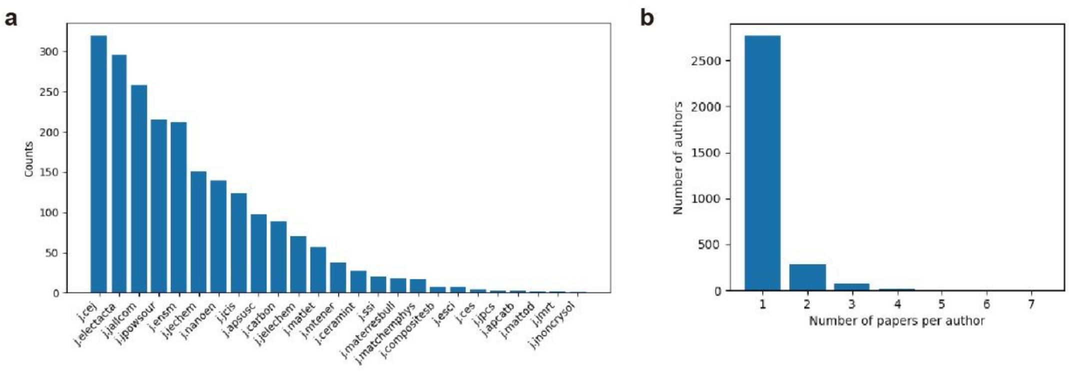

Supplementary Figure 26. Data coverage across the journals and chemistries in machine learning dataset. **a** Distribution of publications included in the machine learning dataset across journals (top 25 shown). The dataset is broadly distributed among electrochemistry and energyrelated journals, with no single journal dominating the entries. All journals not shown in the figure contribute fewer than 10 records each. **b** Author frequency distribution of publication groups defined by combined first and corresponding authors. The x-axis represents the number of repeated occurrences per group, and the y-axis represents the number of groups with that frequency, indicating no strong concentration within a limited number of research groups.

[Comment 2]

Fig. 2b labels the first step as "Relevant Text Parsing," while the described operation appears to be paragraph extraction/retrieval/selection using Python scripts, followed by LLM-based classification/extraction. Because "parsing" commonly implies semantic or syntactic analysis, this terminology may overstate what the script does. Please revise the label and briefly describe the extraction heuristic to enhance reproducibility.

We thank the reviewer for this insightful comment regarding the terminology and reproducibility of the text-processing step in Fig. 2b. We agree that the term *"Relevant Text Parsing"* could be misleading, as the operation does not involve full syntactic or semantic parsing. Accordingly, we have revised the label in Fig. 2b from "Relevant Text Parsing" to "Relevant Paragraph Extraction" to more accurately reflect the actual procedure.

To improve clarity and reproducibility, we have revised the supplementary information to explicitly describe how the relevant paragraphs are extracted. (1) In the Methods section,

paragraphs are extracted directly from structured Elsevier XML files using predefined section headers to retrieve the corresponding methodological text. (2) For the Results section, we apply a rule based paragraph selection procedure that identifies and extracts paragraphs containing references to the figure labels associated with the target cell's cyclability graph, such as "Fig.
2," "Figure 2," or "Fig. 2a."

[Revised manuscript, page 7]

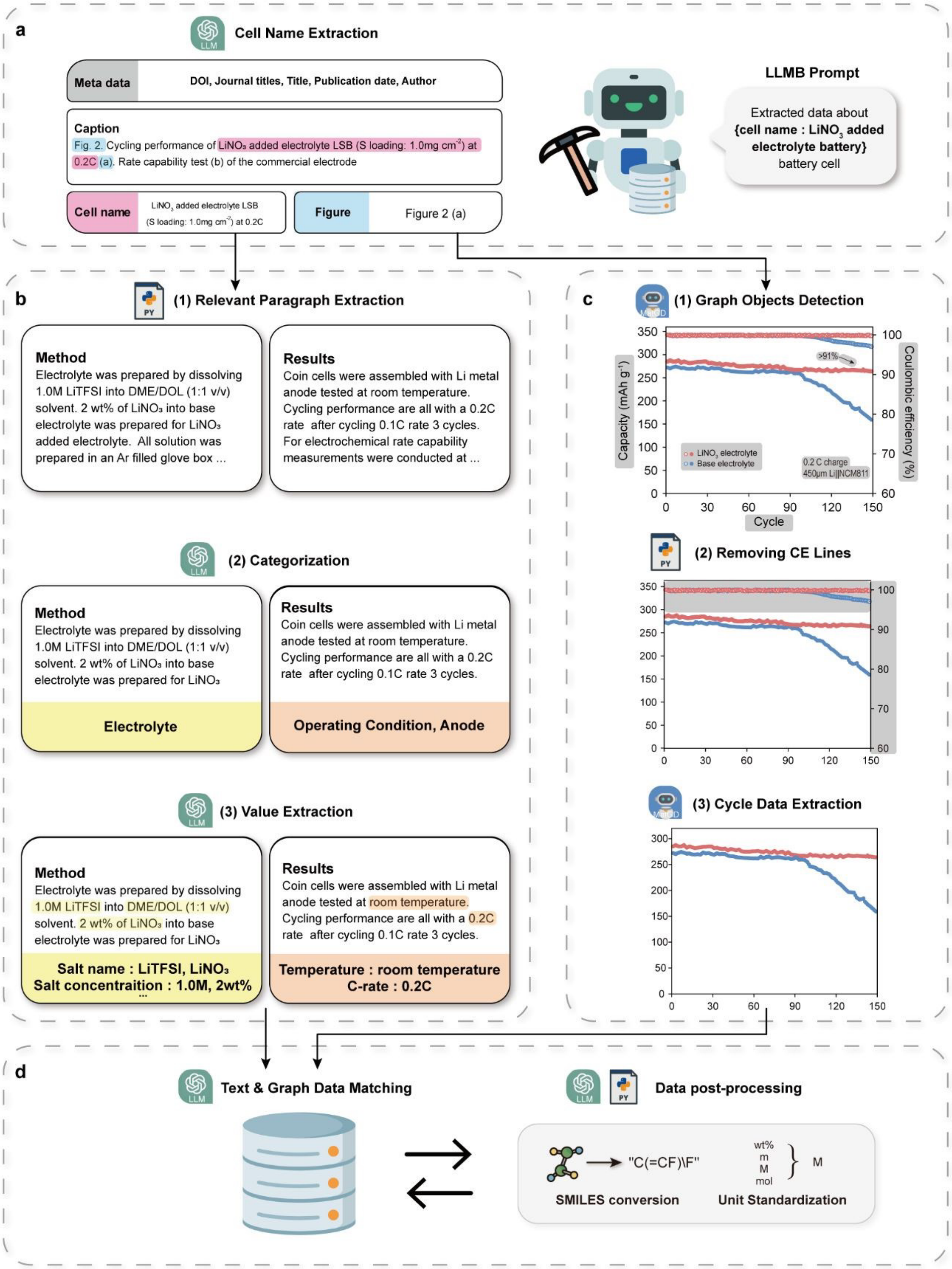

Fig. 2: Schematic illustration of LLMB agent

[Revised Supplementary Notes]

Supplementary Note 6. Relevant paragraph extraction

Relevant paragraphs are extracted using section specific procedures. In the Methods section, paragraphs are directly retrieved from structured Elsevier XML files based on predefined section headers to obtain the corresponding methodological descriptions. In the Results section, a rule based paragraph selection procedure is applied to identify paragraphs that reference the figure labels associated with the target cell's cyclability graph, such as "Fig. 2," "Figure 2," or "Fig. 2a." Paragraphs containing these references are extracted as relevant textual information for subsequent analysis.

[Comment 3]

The manuscript limits the literature corpus to Elsevier sources but suggests this ensures diversity in publication formats. This is not well supported and may introduce publisherspecific style/format biases. Please rephrase the statement more conservatively and discuss potential publisher-format bias and its implications for generalization (or provide a small external validation to demonstrate transferability).

We thank the reviewer for raising an important concern regarding potential bias that may arise from limiting the literature corpus to Elsevier sources. We agree that our original statement may have been overstated, and we have revised it to adopt a more conservative interpretation. The decision to focus on Elsevier journals was primarily driven by the need to ensure copyright compliant text extraction. We acknowledge that this choice may introduce publisher specific style or formatting biases, and we now explicitly discuss this limitation and its potential implications for model generalization in the revised manuscript.

At the same time, we note that the corpus spans a broad range of journals within Elsevier, covering diverse scopes, article structures, and writing styles, as well as substantial variability introduced by different authors. This diversity at the journal and author partially mitigates concerns related to uniform formatting.

To further address generalizability beyond a single publisher, we conducted a small external validation using openly accessible articles from non-Elsevier journals, including ACS Central Science, Science Advances, and Joule. Using these articles, we evaluated the transferability of the proposed extraction and analysis pipeline and confirmed that the workflow remains applicable and achieves consistently high accuracy. The detailed results of this external validation are provided in Supporting Table 15.

In addition, we performed experimental validation on battery material systems that were not present in the training dataset. Model predictions for these previously unseen systems were compared with experimentally measured performance, providing a concrete example of how a model trained exclusively on literature from Elsevier journals can be meaningfully extended to external datasets. Together, these results offer an indirect yet practical demonstration of transferability beyond the original training corpus.

[Revised Manuscript, page 24]

Elsevier Journal Paper Crawling

… We note that restricting the corpus to Elsevier journals may introduce publisher related stylistic or formatting characteristics that are not necessarily representative of the broader battery literature. Therefore, interpretations of model performance and generalizability should be understood within the context of the selected corpus.

[Revised Supplementary Information]

		**ACS Central Science**	**Science Advances**	**Joule**
Number of cells		0	**1**	0
Cathod	Name cathode	0	0	0
	Active material	0	0	0

	ratio_of active_material	0	0	0
	Conductive carbon	0	0	0
	Binder	0	0	0
	ratio of cathode weight	0	0	0
	area loading of active_material	0	0	0
Electrolyte	Name electrolyte	0	0	0
	Li_salt material	0	0	0
	Li_salt concentration	0	0	0
	Li_salt concentration unit	0	0	0
	Solvent material	0	0	0
	Solvent volume ratio	0	0	0
	Solvent volume ratio_unit	0	0	0
	EA ratio	0	0	0
Anode	Name anode	0	0	0
	thickness	0	0	0
	unit	0	0	0
	additional layer	0	0	0
Separator	Separator material	0	0	0

Current collecto	collector1 (cathode)	0	0	0
	collector2 (anode)	0	0	0
Measuremen condition	C-rate	**1**	0	0
	Current density	0	0	0
	Current density unit	0	0	0
	Temperature	0	0	0
	Temperature unit	0	0	0

Supplementary Table 15. External validation of the text mining error across different publishers.

We manually verified the accuracy of the proposed mining workflow applied to selected articles published in ACS Central Science[1], Science Advances[2], and Joule[3]. The table summarizes the number of errors for each article. One cell was not identified in the Science Advances article due to insufficient reference cell information in both the result section and the figure captions. In the ACS Central Science article, one C-rate value was not extracted because the information was provided only within the cycle graph and was not accessible through text mining.

1. Ma P, Mirmira P, Amanchukwu CV. Effect of building block connectivity and ion solvation on electrochemical stability and ionic conductivity in novel fluoroether electrolytes. ACS Central Science 7, 1232-1244 (2021).

2. Xiong Q, et al. A practical 4.8-V Li||$LiCoO_2$ battery. Science Advances 11, eadx5020 (2025).

3. Chang Z, et al. A liquid electrolyte with de-solvated lithium ions for lithium-metal battery. Joule 4, 1776-1789 (2020).

[Comment 4]

The workflow assumes coulombic efficiency (CE) "stabilizes around 100% after a few cycles," and uses this assumption to remove CE curves during graph mining. This may not hold for many lithium-metal battery reports where CE can remain below 100% or fluctuate. Please provide a quantitative check or an ablation study showing how CE removal affects mined curve yield and key dataset statistics.

We thank the reviewer for this comment. The purpose of graph mining in this work is to extract cyclability data from published graphs. In some figures, CE curves are plotted together with cycling curves within the same graph, which can lead to their unintended extraction during graph mining. In many cases, CE is displayed as a separate curve positioned in the upper region of the graph, while the cycling capacity curve appears in the lower region. The statement in the original manuscript that CE "stabilizes around 100% after a few cycles" was not intended to imply a physical assumption about CE behavior, but rather to describe the typical graphical placement of CE near the top of the plot. We recognize that this wording was ambiguous and could lead to misunderstanding. We have therefore revised the manuscript to clarify this point. **[Revised Manuscript, page 9]**

When coulombic efficiency (CE) curves were present in the graphs, they were removed using a Python-based algorithm that utilized the characteristic behavior of CE, which is typically positioned at the top of the cycle graph and therefore can be readily identified.

To address this issue, CE curves were excluded during graph mining process. In cases where CE and cycling curves overlap or are not clearly separable, reliable extraction of the cycling curve using MatGD is not possible. This is because CE and cycling curves are often distinguished by marker shape rather than color, whereas MatGD treats curves with the same color as a single data series. To avoid errors, such cases were manually identified and excluded after graph mining. The Methods section has been revised accordingly to provide a clearer description of this procedure.

[Revised Manuscript, page 25]

A threshold was set based on the observation that CE values typically positioned above the cycle graph line. By using this threshold, CE data could be effectively identified and eliminated, allowing for the isolation of the capacity data line.

[Comment 5]

Figs. 4 (overall), 4b, and 5b: Several labels are too light and/or too small, and font type/size are not fully consistent within Fig. 4. Please standardize font family, font size hierarchy, and contrast across these panels. Fig. 5c: Please add a clear legend indicating which element corresponds to each sphere color in the ball-and-stick model. Fig. 5d: Please include a colorbar and specify the isosurface value(s) and explicitly define whether red/blue represent positive/negative charge (or electron accumulation/depletion), to avoid ambiguity. After these issues are satisfactorily addressed, I believe the manuscript will be substantially strengthened and can be recommended for publication in ACS Central Science.

We thank the reviewer for the careful and constructive comments regarding figure clarity and readability. We have revised the figures accordingly to address all points raised. Specifically, we standardized the font family, font size hierarchy, and contrast throughout Figures 4 (overall), 4b, and 5b to ensure visual consistency and improved legibility. Labels that were previously too small or light have been adjusted for clarity. For Figure 5c, we have revised the caption to explicitly indicate the correspondence between sphere colors and their respective chemical elements in the ball-and-stick model.

For Figure 5d, we have specified the isosurface value and explicitly defined the red and blue regions as electron accumulation and depletion (corresponding to negative and positive charge density), thereby eliminating ambiguity. Regarding the color scale, the visualization was performed using Avogadro, which automatically generates charge density difference isosurfaces from Gaussian output files but does not provide explicit numerical values for the color bar or for the charge density corresponding to each color. To address this limitation and improve quantitative transparency, we have additionally provided the DFT calculated atomic charge values for each molecule in the Supporting Information.

[Revised Supplementary Information]

Diethyl carbonate					
**Atom**	**No**	**Atomic charge**	**X (Å)**	**Y (Å)**	**Z (Å)**
O	1	-0.55437	1.083413	-0.406678	-0.000015

O	2	-0.55437	-1.083414	-0.406678	-0.000013
O	3	-0.64478	0	1.586134	0.000013
C	4	-0.03298	2.358872	0.28195	-0.000006
C	5	-0.03298	-2.358869	0.281949	-0.000001
C	6	-0.59434	3.443039	-0.775191	0.000016
C	7	-0.59434	-3.44304	-0.77519	0.000011
C	8	1.02589	0.000001	0.378253	-0.000013
H	9	0.18466	2.410454	0.922232	-0.883544
H	10	0.18466	2.410434	0.92225	0.88352
H	11	0.18466	-2.410435	0.922241	0.88353
H	12	0.18466	-2.410452	0.922239	-0.883533
H	13	0.20753	4.424225	-0.292815	0.000019
H	14	0.20715	3.371411	-1.40904	0.886413
H	15	0.20715	3.371428	-1.409059	-0.88637
H	16	0.20753	-4.424224	-0.29281	0.000015
H	17	0.20714	-3.371428	-1.409054	-0.886377
H	18	0.20714	-3.371419	-1.409044	0.886405

**Ethyl methyl carbonate**

**Atom**	**No**	**Atomic charge**	**X (Å)**	**Y (Å)**	**Z (Å)**
O	1	-0.553	-0.645398	-0.441624	-0.000009
O	2	-0.54212	1.494817	-0.776633	-0.000004
O	3	-0.64299	0.734476	1.359117	0.000002
C	4	-0.03313	-1.800359	0.434878	0.000003
C	5	-0.59452	-3.033799	-0.443394	0.000008
C	6	1.02285	0.54422	0.166767	-0.000019
C	7	-0.20896	2.846619	-0.284957	0.000011
H	8	0.18497	-1.752568	1.075214	0.883658
H	9	0.18497	-1.75258	1.075221	-0.883648
H	10	0.20789	-3.929084	0.184102	0.000016
H	11	0.2075	-3.060467	-1.08072	-0.886378
H	12	0.2075	-3.060455	-1.080727	0.886388
H	13	0.18562	3.035255	0.317013	-0.889908
H	14	0.1878	3.473422	-1.173765	0.000027
H	15	0.18562	3.03523	0.317027	0.889925
**Dimethyl carbonate**					

Atom	No	Atomic charge	X (Å)	Y (Å)	Z (Å)
O	1	-0.54062	1.082852	-0.709587	-0.000074
O	2	-0.54062	-1.082853	-0.709586	-0.000072
O	3	-0.64109	0	1.28266	0.000133
C	4	1.01973	0	0.075672	0.000005
C	5	-0.20913	2.343383	-0.01621	-0.000003
C	6	-0.20913	-2.343383	-0.01621	0.000001
H	7	0.18598	2.437057	0.607365	-0.890055
H	8	0.18848	3.099045	-0.798331	-0.000033
H	9	0.18598	2.437016	0.607258	0.890128
H	10	0.18848	-3.099046	-0.79833	-0.000016
H	11	0.18598	-2.437064	0.607356	-0.890057
H	12	0.18598	-2.43701	0.607269	0.890125

Supplementary Table 12. Natural atomic charges (NPA) and Cartesian coordinates of DEC, EMC, and DMC molecules.

[Revised Manuscript, page 16]

Fig. 5: Prediction of 50th capacity and solvation structure analysis. Result of 50th cycle capacities predicted from the random forest model with the mined database **a** and the **b** SHAP analysis. **c** Solvation structures from MD simulations of NCM811 cells with different solvents; oxygen, carbon, hydrogen, fluorine, phosphorus, and lithium atoms are shown in

red, gray, white, cyan, purple, and violet, respectively. **d** Atomic charges of DEC, EMC, and DMC molecules from OPLS and DFT (red and blue indicate electron accumulation and depletion, respectively; the isosurfaces are shown at an isovalue of 0.05 e$Å^{-3}$).

Reviewer 2

Overall Comment:

This manuscript presents a significant advancement in applying AI agents to the study of lithium-metal batteries. By leveraging a large language model to extract data from both textual and graphical sources, the work not only predicts initial battery capacity but also deepens the understanding of the relationships between electrochemical performance and battery components. The inclusion of experimental validation further strengthens the credibility of the proposed approach. Several key aspects could be clarified or expanded to improve the rigor, interpretability, and generalizability of the findings. Below are specific comments and suggestions intended to strengthen the manuscript.

We thank the reviewer for the positive and thoughtful assessment of our manuscript and appreciate the recognition of the significance of applying AI agents to lithium metal battery research, as well as the value of integrating text and graphical data extraction with experimental validation. In response to the reviewer's comments, we have carefully revised the manuscript to improve methodological rigor, interpretability, and generalizability by clarifying the data curation and extraction workflows, expanding discussions on feature selection and physicochemical relevance, strengthening validation and bias-related analyses, refining the presentation of figures and supplementary materials, and more explicitly defining the scope and limitations of the proposed framework where appropriate.

[Comment 1]

In the graph mining stage, only cyclability data were extracted after removing Coulombic efficiency (CE) curves. Since CE is a critical indicator of the reversibility of Li plating/stripping and a strong predictor of cycle life in lithium-metal batteries, its exclusion may limit the feature set and model performance. Please justify the omission of CE or consider incorporating CErelated metrics (e.g., average CE or cycle number of CE declining rapidly) into the feature extraction process.

We appreciate the reviewer's comment and agree that Coulombic efficiency (CE) is a critical electrochemical metric of battery performance. However, CE was excluded from the input features in this study because it represents a downstream electrochemical outcome rather than an intrinsic material descriptor. The primary objective of our modeling framework is to explore the material-performance space and to identify how material composition and operating parameters give rise to electrochemical behavior. Incorporating CE as an input feature would confound the interpretation of material-performance relationships by embedding performance outcomes directly into the feature space.

Furthermore, in many reported stable LMB systems, CE is typically reported within a very narrow range close to 100%. Compared with the broader variance observed in capacity data, such limited variation is difficult to capture with sufficient numerical reliability. Therefore, incorporating CE would not provide statistically robust information within the current dataset.
For these reasons, CE was not included among the input features.

[Comment 2]

The accuracy of graph mining has not been evaluated (Table 1). Without validation, potential extraction errors could propagate into the model and affect conclusions. The authors should extract cyclability data (such as capacity retention) from the discussion part or tables in the papers and compare the data from graph mining to validate data credibility.

We thank the reviewer for highlighting the importance of validating the accuracy of the graph mining process. We agree that extraction errors could propagate into downstream analyses, and we have clarified and strengthened this aspect in the revised supplementary information.

As described in the manuscript, all graph mining results used for model training were manually inspected after extraction, and only validated data were retained. This step removes obvious extraction errors prior to downstream analysis.

To quantitatively evaluate the accuracy of graph mining, we performed an explicit validation using cyclability data generated in-lab during the LMB experiments reported in this study. For these experiments, the experimentally measured capacity values at selected cycle numbers were directly compared with the values re-extracted from the corresponding cyclability graphs using MatGD. These results demonstrate that MatGD accurately captures both the overall curve shape and the numerical capacity values at individual data points with high fidelity. We note that directly reproducing and publishing

cyclability graphs from previously published articles for validation is constrained by copyright restrictions.

[Revised Supplementary Information]

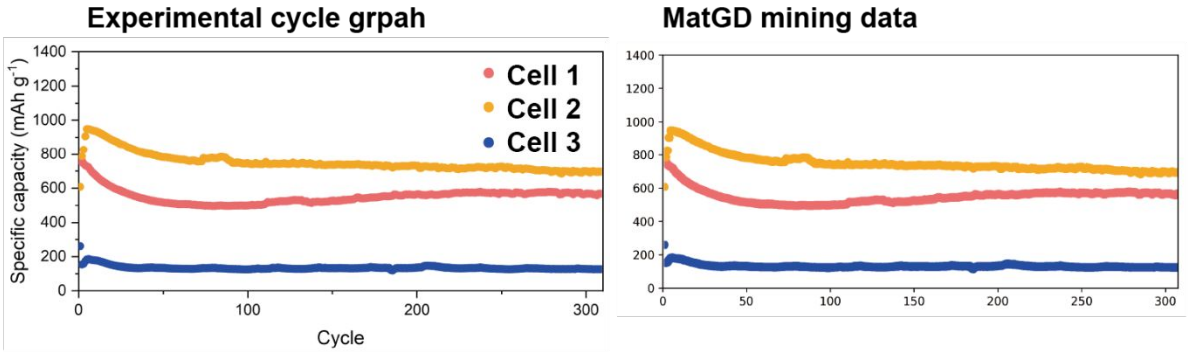

Supplementary Figure 23. Comparison between experimental data and MatGD extracted data

**Cell 1**	**Capacity of 5th cycle**	**Capacity of 50th cycle**	**Capacity of 100th cycle**	**Capacity of 200th cycle**	**Capacity of 300th cycle**
**Experimental Value (mAh/g)**	724.70	512.22	495.07	559.78	562.05
**MatGD Mining value (mAh/g)**	722.50	514.56	497.54	563.40	566.36
**Error (mAh/g)**	-2.2	2.34	2.47	3.62	4.31
**Cell 2**	**Capacity of 5th cycle**	**Capacity of 50th cycle**	**Capacity of 100th cycle**	**Capacity of 200th cycle**	**Capacity of 300th cycle**
**Experimental Value (mAh/g)**	178.36	128.87	119.55	130.72	122.33

**MatGD Mining value (mAh/g)**	175.64	131.24	122.36	133.46	124.58
**Error (mAh/g)**	-2.72	2.37	2.81	2.74	2.25
**Cell 3**	**Capacity of 5th cycle**	**Capacity of 50th cycle**	**Capacity of 100th cycle**	**Capacity of 200th cycle**	**Capacity of 300th cycle**
**Experimental Value (mAh/g)**	946.16	779.80	743.54	728.48	697.16
**MatGD Mining value (mAh/g)**	943.76	781.70	743.96	725.46	698.08
**Error (mAh/g)**	2.40	-1.90	-0.42	3.02	-0.92

Supplementary Table 3. Comparison of values at 5, 50, 100, 200, and 300 cycles between experimental data and MatGD extracted data

Supplementary Note 5. Graph mining details

To quantitatively evaluate the accuracy of the graph mining procedure, we performed a direct validation using cyclability data generated in-house during the lithium metal battery experiments reported in this study. Cyclability graphs obtained from these experiments were processed using the same graph mining workflow (MatGD) applied to the literature data. The extracted values were compared with the corresponding ground-truth experimental capacities at selected cycle numbers. A representative comparison between the experimental curves and the graph mining data is shown in **Supplementary Figure 23**.

For three independent cells, capacity values at the 5th, 50th, 100th, 200th, and 300th cycles were extracted from the graphs and directly compared with the original experimental data.

As summarized in **Supplementary Table 13**, the graph mining data showed excellent agreement with the ground-truth data across all tested cycle numbers and cells, with only minor deviations, typically within a few mAh/g.

[Comment 3]

In Figures 4 and 5, many feature abbreviations remain unexplained beyond EState VSA6 and Kappa3. Additionally, the rationale for selecting these specific molecular or electrochemical descriptors is not discussed. Please provide a supplementary table for all feature abbreviations used in the SHAP plots. A brief discussion on why these features were chosen, linking their core physicochemical significance to battery performance, would greatly enhance the interpretability and domain relevance of the analysis.

We thank the reviewer for pointing out the need to clarify feature abbreviations and the rationale for descriptor selection in the SHAP analysis. We agree that clearer explanation of these features is important for improving interpretability and domain relevance. In response, we have added a comprehensive supplementary table that lists and defines all feature abbreviations used in the SHAP plots (Figures 4 and 5), together with brief descriptions of their physical or chemical meaning.

From the text mining, we were able to extract the identities and relative amounts of individual molecular components constituting each electrolyte. Rather than directly encoding molecular identities as one-hot vectors, which would provide limited physical interpretability and poor generalization, we intentionally selected descriptors that capture underlying physicochemical properties relevant to battery behavior.

Specifically, we incorporated molecular descriptors computed using the RDKit library to reflect intrinsic properties of the molecules, such as topology, electronic characteristics, and molecular complexity. In addition, we included domain informed descriptors such as the inorganic to organic atom ratio and Li^+ cluster features derived from solvation structure analysis.

[Revised Supplementary Information]

Descriptor	Explanation
Ni	The proportion of nickel within the cathode active material.

Mn	The proportion of manganese within the cathode active material.
Co	The proportion of cobalt within the cathode active material.
C rate	A measure of the charge or discharge current relative to the nominal capacity of a battery.
Loading	The amount of electrochemically active material coated on the electrode per unit area (mg/cm^2).
Solvent EState VSA6	MOE-type descriptors based on electrotopological state indices combined with surface area contributions as reported in J. Chem. Inf. Comput. Sci., 31, 76–81 (1991). The Estate VSA6
	descriptor corresponds to the summed surface area of atoms whose EState values fall within the range 1.54 < EState < 1.81.
Solvent Kappa3	A descriptor of molecular shape and branching complexity of solvent molecules, quantified by the Hall-Kier kappa3 value calculated using equations (58), (61), and (62) in Reviews in Computational Chemistry, Vol. 2, pp. 367-422 (1991).
lnOr	The inorganic-to-organic ratio, which quantifies the balance between inorganic species and organic solvent molecules in the solvation structure.
Solvent MinAbsEStateIndex	The minimum value of EState indices for the solvent molecules.
Solvent SMR VSA6	MOE-type descriptor based on molar refractivity and surface area contributions (6th bin, 2.75 < x< 3.05), as reported in J. Mol. Graph. Mod., 18, 464–477 (2000).

Li Clusters	The average number of lithium atoms within lithium clusters in the solvation structure. A lithium cluster is defined as an aggregate of lithium atoms and surrounding anions or solvent molecules connected through Li-X interactions within a distance cutoff of 2.5Å.
Solvent BertzCT	A topological index of solvents that quantifies the structural complexity of solvent molecules. It consists of the sum of two terms: one representing bonding complexity and the other representing the complexity of heteroatom distribution.
Solvent 1.9	The fraction of solvent molecules within a 1.9 Å cutoff distance from a Li atom in the solvation structure.
Solvent Chi0v	A valence molecular connectivity index of solvent molecules calculated using equations (1), (9), and (10) from Reviews in Computational Chemistry, Vol. 2, pp. 367-422 (1991).

Supplementary Table 10. Definitions of descriptors used in the SHAP analysis.

[Comment 4]

The manuscript employs several machine learning models (RF, XGBoost, GBR, etc.), but the rationale for their selection and the impact of this choice on the results is not clarified.

We appreciate the reviewer’s comment regarding the choice of machine learning models. We compared multiple machine learning models using the PyCaret library to ensure a systematic evaluation. The extracted battery property dataset contains a mixture of numerical and categorical features, and their prediction of cyclability performance relies strongly on nonlinear interaction among these variables. As a result, tree-based models such as Random Forest (RF), XGBoost, and Gradient Boosting Regressor (GBR), consistently demonstrated superior performance across our benchmarks.

In addition to predictive accuracy, a key objective of this study is to provide interpretability of the relationships between material features and cyclability performance. Accordingly, we prioritized models for which SHAP analysis can be reliably applied, enabling physically meaningful interpretation of model predictions. The models presented in the manuscript

satisfy these criteria and exhibited strong predictive performance. We have clarified this rationale in the revised manuscript.

[Revised Manuscript, page 27]

Feature Generation and Machine Learning

… Multiple machine learning models were evaluated using the PyCaret library to enable a systematic and fair comparison under identical conditions. Because the dataset contains mixed numerical and categorical features with strong nonlinear interactions, tree-based models consistently demonstrated superior performance. Accordingly, Random Forest (RF), XGBoost, and Gradient Boosting Regressor (GBR) were selected as representative models for this study.

All models were trained with 10-fold cross-validation.

[Comment 5]

Figure 7 provides a prediction and validation of the 200th-cycle capacity for Li–S batteries, but SHAP analysis is absent for this system. We also strongly suggest that the authors consider incorporating the chemical composition and thickness of the Li metal anode into the SHAP analysis, which could reveal important mechanistic differences among all LMBs. One should note that Li metal anodes are case-by-case divergent in existing literature. [https://doi.org/10.1002/adma.202511817.]

We appreciate the reviewer's suggestion. In response, we have added the SHAP analysis of the 200th-cycle capacity for Li-S batteries (LSBs) in the revised SI Figure 27. As shown by the SHAP analysis, the initial capacity is identified as the most important feature for predicting capacity in LSB. Ideally, excluding initial capacity would be favorable for analyzing materialperformance relationships. However, when initial capacity is removed from the input features, the predictive performance of the model decreases significantly.

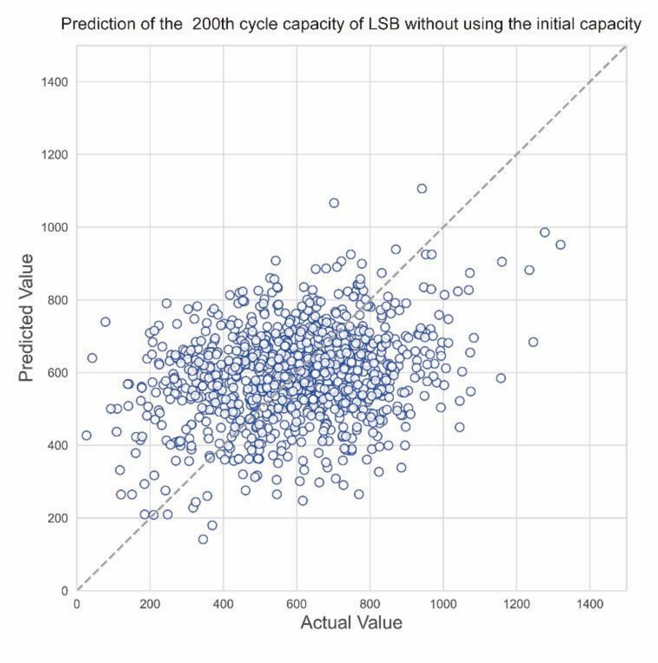

This performance degradation appears because key information related to sulfur host materials, which is related to activation kinetics of sulfur, cannot be incorporated into the machine learning model due to limitations in literature reporting and digitization, as discussed in the manuscript. Therefore, initial capacity was retained to maintain predictive fidelity, while acknowledging its indirect nature. To address these limitations, future progress will require more structured reporting of sulfur host material information in the literature, including structure and synthesis-dependent features. In addition, representing host materials using graph-based or embedding approaches offers a promising direction.

We also agree with the reviewer that the chemical composition and thickness of the Li anode are important in LMB mechanism. In this work, we fixed the anode composition to pure Li metal, as incorporating Li alloy would introduce additional chemical variability. However, the number of Li anode thickness information was sparsely reported in the literature. Even among the collected thickness data (Supplementary Figure 8), only a small subset of cells contained complete accompanying information required for model input. Of the 712 LSB data points used in the model shown in Figure 7a, only 184 included anode thickness information. We further evaluated the relationship between anode thickness and the 200th cycle capacity. Given the limited sample size and the fact that capacity is predicted alongside multiple other features rather than anode thickness alone, the correlation was found to be negligible.

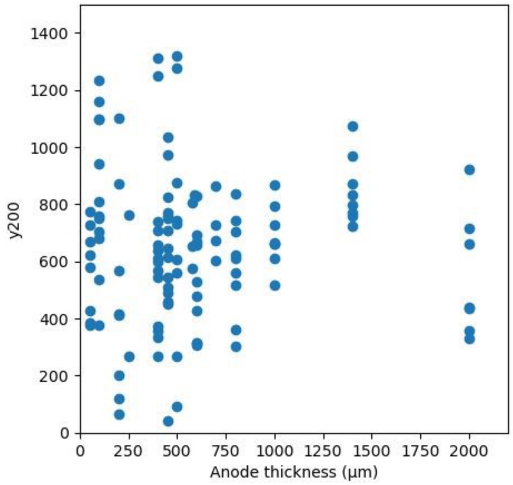

This limitation and its implication are now clarified in the revised manuscript.

[Revised Supplementary Information]

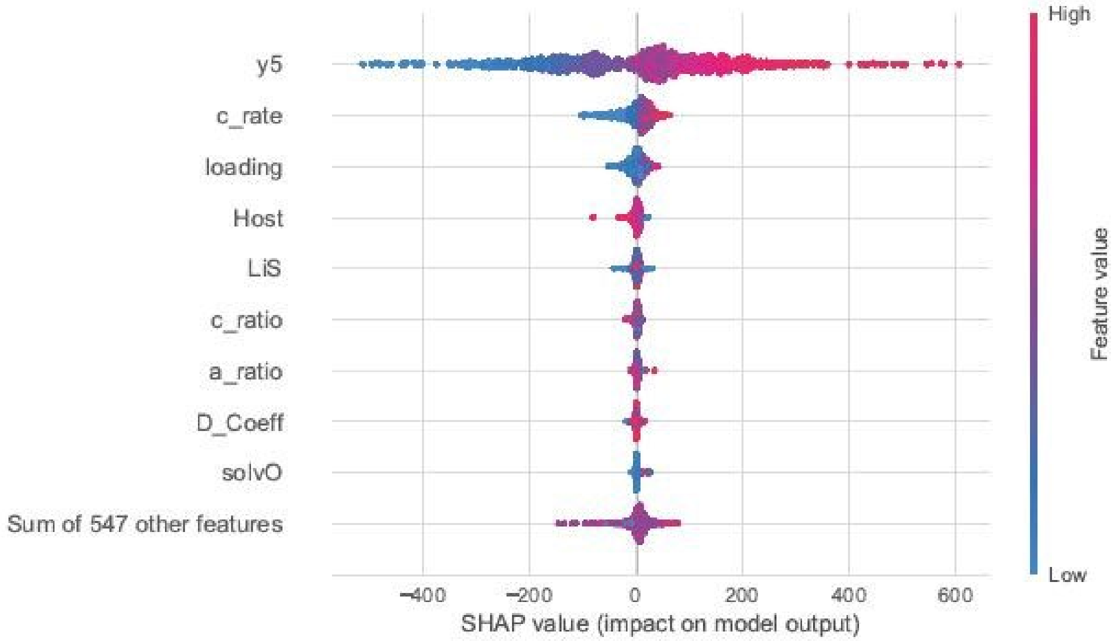

Supplementary Figure 27. SHAP plot of 200th cycle capacity prediction model for lithium sulfur battery

[Revised Manuscript, page 23]

Moreover, the predictive capability of this framework can be further strengthened by increasing the availability of well-curated and comprehensive datasets. Several features extracted by LLMB were too sparsely reported to be incorporated into the machine learning model; for example, Li metal anode-related parameters were identified but were insufficient in number of robust models which are known to critically affect battery performance.[34] More standardized reporting and complementary data generation through self-driving laboratories would enable the accelerate materials innovation across a broad range of scientific domains.

34. Hu Y, *et al*. Review of thin lithium metal battery anode fabrication–microstructure–electrochemistry relations. *Advanced Materials* e11817 (2025).

[Comment 6]

The machine learning models rely entirely on data mined from published papers, where very often only the best-performing results and the performance gap between experimental and control groups were exaggerated. This could introduce bias, especially for predicting cycle life. Please discuss this potential limitation.

We thank the reviewer for raising this important point regarding potential publication bias. We agree that published papers may preferentially reflect only the best performance results and the performance gap between experimental and control groups can be exaggerated. Our prediction results (Figure 4a, 5a, 7) indicate that the model maintains consistent predictive performance across both low- and high-performing systems. We find that the model predictions capture consistent performance ordering and degradation trends across these systems, indicating that relative performance relationships are preserved even when absolute performance values may be biased. This suggests that, while publication bias affects the overall performance distribution, it does not fundamentally alter the comparative trends learned by the model.

Reviewer 3

Overall Comment:

This work reports a multi-modal AI agent integrating LLM-based text mining and MatGDbased graph mining to construct a structured LMB materials-property database from literature, followed by machine learning analysis and experimental validation. This work provides a rich and comprehensive visualization analysis of materials reported in previously published lithium metal battery studies, offering meaningful insights into the global landscape of lithium metal batteries. In addition, the proposed predictive framework innovatively accounts for the coupled effects of both electrode and electrolyte materials on battery performance.

We thank the reviewer for the thoughtful and positive evaluation of our work. We appreciate the recognition of the multi-modal nature of the proposed AI agent, which integrates LLMbased text mining and graph mining tool MatGD to construct a structured materials-property database for lithium metal batteries, as well as the value of combining machine learning analysis with experimental validation.

The reviewer's questions have been helpful in improving the clarity of the scope, limitations, and intent of the proposed framework. In response, we have expanded the manuscript to improve clarity, address potential limitations and sources of bias, and enhance transparency and reproducibility.

[Comment 1]

In LMBs, additives play a pivotal role in electrolyte engineering. Does the database constructed in this work incorporate additive-level information, and can it predict how additives impact cell performance?

We appreciate the reviewer's important question regarding the role of electrolyte additives. The constructed database does incorporate additive-level information; however, two factors limit the extent to which additive effects can be directly isolated and interpreted in the current analysis.

Fundamentally, the effect of additives in lithium metal batteries is inherently interaction driven rather than additive in a single feature sense. Additives almost always function through coupled interactions, such as salt-solvent-additive combinations. In contrast, the SHAP analysis employed in this work primarily quantifies marginal contributions of individual features and does not explicitly resolve higher order interaction effects. As a result, additive contributions are often distributed across correlated solvent or salt features, leading to lower apparent importance for additives. As shown in the table below,

although additives are clearly present in the electrolyte and capacity variations are observed in these cells within our database, individual additives do not appear as dominant features in the SHAP analysis. This serves as evidence that additive effects are not independently captured in the current SHAP interpretation. Below, we present representative examples of cells containing electrolyte additives from our database. We emphasize that this behavior reflects a structural limitation of single feature SHAP interpretation rather than the absence of additive information in the model.

doi	Electrolyte	Initial Capacity	100th cycle Capacity
①	0.1M $LiNO_3$ and 1M LiTFSI in DME/DOL (1:1) + 0.01M TTF	1028.6	771.0
	0.1M $LiNO_3$ and 1M LiTFSI in DME/DOL (1:1)	982.5	446.1
②	1M LiTFSI in DME/DOL (1:1)	122.2	61.9
	1M LiTFSI in DME/DOL (1:1) + 25v% [MEMP][TFSI]	119.7	102.4
③	1M LiTFSI in DME/DOL (1:1)	941.0	800.6
	1M LiTFSI in DME/DOL (1:1) + 2wt% $LiNO_3$	1052.3	890.8
	1M LiTFSI in DME/DOL (1:1) + 2wt% $ZrO(NO_3)_2$	1082.8	957.4
④	1M LiTFSI in DME/DOL (1:1) with 2wt% LiNO3	805.8	647.3
	1M LiTFSI in DME/DOL (1:1) with 2wt% LiNO3 + 1wt% TFMSA	839.2	720.7
⑤	1M $LiPF_6$ in EC/DMC/EMC (1:1:1)	207.2	174.4
	1M $LiPF_6$ in EC/DMC/EMC (1:1:1) + 1.0wt% TMOBX	204.1	190.9
	1M $LiPF_6$ in EC/DMC/EMC (1:1:1) + 1.5wt% TMOBX	206.4	178.4

① 10.1016/j.jpowsour.2022.231482 ② 10.1016/j.electacta.2020.137535 ③ 10.1016/j.jpowsour.2019.227232 ④ 10.1016/j.cej.2022.136489 ⑤ 10.1016/j.esci.2022.05.003

[Comment 2]

As the dataset is limited to Elsevier publications, whether known state-of-the-art electrolytes or electrodes are captured in the database should be checked (especially those from other publishers) to ensure the representativeness.

We thank the reviewer for raising an important concern regarding the representativeness of the dataset and the coverage of state-of-the-art (SOTA) electrolytes and electrodes beyond

Elsevier publications. In response to this comment, we manually identified representative SOTA electrolyte systems reported in non Elsevier journals that can be mapped onto the feature space used in our model and evaluated them using the trained capacity prediction model.

Using our reported machine learning model based on molecular descriptors and one-hot encoding, we evaluated representative SOTA systems that could be mapped onto the predefined feature space of our model. Because the model is trained within this descriptor space, only electrolyte components represented in the dataset were considered. We further tested cells that exhibited higher Ni content and initial capacities beyond the range represented in the collected dataset. Despite these extended conditions, the model demonstrated strong agreement with the experimentally reported initial capacities, as shown below.

Actual Value (Initial Capacity)	Prediction value (Initial Capacity)
215.11 mAh/g	206.39 mAh/g

Wang Z, et al. An intrinsically nonflammable electrolyte for prominent-safety lithium metal batteries with high energy density and cycling stability. Advanced Functional Materials 33, 2215065 (2023). (10.1002/adfm.202215065)

We agree that state-of-the-art (SOTA) materials may be reported in journals beyond the Elsevier publications and that restricting the dataset to a single publisher could potentially limit coverage. The restriction to Elsevier publications was primarily motivated by copyright and xml format full-text accessibility considerations, which are essential for reliable text and graph mining at scale. This constraint was applied to ensure legal and technical feasibility of largescale data extraction. Importantly, we emphasize that the primary objective of the proposed framework is not to directly predict the absolute performance of newly discovered SOTA materials, but rather to elucidate material-performance relationships of LMBs within the design space defined by the training data. In this sense, the model is intended as a knowledge extraction and analysis tool, rather than a discovery model.

To assess applicability beyond the training corpus, we further validated the model using experimental data generated in this study, which was not included in the training dataset. The consistency between model predictions and experimental trends demonstrates that the framework can be extended to previously unseen systems within a chemically meaningful design space.

[Comment 3]

The descriptors used for solvents in this study do not incorporate quantum-level information (such as HOMO, LUMO), which is crucial for electrochemical stability. Could the inclusion of quantum-chemical descriptors lead to more accurate predictions of electrochemical stability?

We thank the reviewer for this insightful suggestion regarding the inclusion of quantumchemical descriptors such as HOMO and LUMO energy levels.

To directly evaluate this point, we incorporated DFT derived HOMO and LUMO values into the capacity prediction model and compared the results with the original model that did not include quantum-level descriptors. For the initial capacity prediction, the predictive performance remained essentially unchanged. The R^2 value changed only marginally from 0.7530 to 0.7536, and the MAE showed no improvement, changing from 10.68 to 10.72 mAh/g. The top-ranked SHAP features were also largely preserved. For the 50th-cycle capacity prediction, the same conclusion holds: adding HOMO and LUMO resulted in only negligible differences in performance, with R^2 remaining effectively the same (0.6866 vs 0.6891) and MAE changing only slightly (12.60 vs 12.48 mAh/g). The dominant SHAP features remained consistent, indicating that the quantum chemical descriptors did not materially affect either accuracy or feature attribution in this setting.

Given these results, we did not adopt DFT derived descriptors in the final descriptor set for two practical and conceptual reasons within the scope of this study. First, electrolytes in our dataset are multi component mixtures containing different solvents and salts across a range of molar ratios. This complexity prevents a physically faithful mapping of single molecule quantum descriptors into an electrolyte level representation. Accordingly, we represent solvent and salt properties using mole fraction weighted arithmetic averages. Applying the same averaging to HOMO and LUMO energies would further weaken physical interpretability because frontier orbital energies are inherently species specific and do not retain a clear chemical meaning when averaged over mixtures. Second, the central objective of this work is to identify correlations between materials information from data mining and electrochemical performance at scale, without requiring additional high-cost quantum calculations for each component.

[Revised Supplementary Information]

Initial Capacity Prediction	Without DFT data	With DFT data
Model	Random Forest	Random Forest

R^2 Score	0.7530	0.7536
MAE(mAh/g)	10.6831	10.7177
Top 10 SHAP features	Ni, Mn, Co, C rate, Loading, Solvent Estate VSA6, Solvent Kappa3, InOr, Solvent MinAbsIndex, Solvent SMR VSA6	Ni, Co, Mn, C rate, Loading ,Solvent Estate VSA6, Solvent Kappa3, Diffusion Coefficient, Solvnet SlogP VSA2, Solvent MinAbsIndex
50th Capacity Prediction	Without DFT data	With DFT data
Model	Random Forest	Random Forest
R^2 Score	0.6866	0.6891
MAE(mAh/g)	12.6029	12.4843
Top 10 SHAP features	Ni, C rate, Loading, Co, Li Clusters, Mn, InOr, Solvent BertzCT, Solvent 1.9, Solvent Chi0v	Ni, C rate, Loading, Co, Li Clusters, Mn, InOr, Solvent BertzCT, ratio of active material

Supplementary Table 14. Performance comparison of predicting models for initial capacity and 50th cycle capacity with and without inclusion of DFT derived descriptors

[Comment 4]

The N/P ratio is an important operating condition. Is this captured in the database, and does it ensure consistency from this standpoint?

We thank the reviewer for this comment and agree that the N/P ratio is an important operating parameter in LMBs. However, explicit reporting of the N/P ratio is very sparse in the literature. Among the 2,549 literature, only 96 papers (3.7%) directly mentioned the N/P ratio. Assuming identical cathode and anode areas, the N/P ratio can be estimated from the cathode loading and the thickness of the Li metal anode. Among the 8,074 collected cells, only 441 cells (5.4%) contained sufficient information on both cathode loading and Li anode thickness to enable such calculation. Furthermore, within the machine learning

dataset (4,065 cells), only 274 cells (6.7%) had complete non-missing features for reliable prediction. Therefore, although N/P ratio is an important feature, the number of samples with sufficient information was too limited to ensure statistical consistency or to incorporate it robustly into the machine learning model. If a sufficient number of N/P ratio data become available, it can be effectively collected and incorporated as an input feature within the LLMB workflow.

[Comment 5]

The search terms for the collection of papers should be provided.

We thank the reviewer for the suggestion that this information should be provided. In Scopus, we used the following query to collect papers related to lithium metal batteries. 'TITLE-ABS-KEY ("Li metal battery" OR "Lithium metal battery" OR "LMB" OR "Lithium metal anode" OR "LSB" OR "Lithium sulfur battery") AND (LIMIT-TO (DOCTYPE , "ar"))'.

The final condition, LIMIT-TO (DOCTYPE , "ar"), was included to restrict the document type to research articles and exclude reviews, letters, and other non-article formats. After this initial crawling, we eliminated papers that did not contain cycle graphs. This step also served to remove papers that share abbreviations such as 'LMB' or 'LSB' but are not actually related to lithium metal batteries. Through this filtering process, the final collection of papers was obtained. We have revised the Methods section of the manuscript as below.

[Revised Manuscript, page 24]

Using the Elsevier Scopus API, we initially retrieved a total of 7,342 lithium metal battery (LMB)related papers through keyword-based searches using 'TITLE-ABS-KEY ("Li metal battery" OR "Lithium metal battery" OR "LMB" OR "Lithium metal anode" OR "LSB" OR "Lithium sulfur battery") AND (LIMIT-TO (DOCTYPE , "ar"))' keyword.
